# Developing a Mixed Neural Network Approach to Forecast the Residential Electricity Consumption Based on Sensor Recorded Data

**DOI:** 10.3390/s18051443

**Published:** 2018-05-05

**Authors:** Simona-Vasilica Oprea, Alexandru Pîrjan, George Căruțașu, Dana-Mihaela Petroșanu, Adela Bâra, Justina-Lavinia Stănică, Cristina Coculescu

**Affiliations:** 1 Department of Economic Informatics and Cybernetics, The Bucharest Academy of Economic Studies, Romana Square 6, Bucharest 010374, Romania; simona.oprea@csie.ase.ro (S.-V.O.); bara.adela@ie.ase.ro (A.B.); 2 Department of Informatics, Statistics and Mathematics, Romanian-American University, Expoziției 1B, Bucharest 012101, Romania; carutasu.george@profesor.rau.ro (G.C.); danap@mathem.pub.ro (D.-M.P.); stanica.lavinia.justina@profesor.rau.ro (J.-L.S.); coculescu.cristina@profesor.rau.ro (C.C.); 3 Department of Mathematics-Informatics, University Politehnica of Bucharest, Splaiul Independenței 313, Bucharest 060042, Romania

**Keywords:** smart homes, forecasting solutions, energy management, home appliances and devices monitored by sensors, residential electricity consumption, Internet of Things (IoT) cloud solution, artificial neural networks (ANNs), non-linear autoregressive with exogenous inputs network (NARX), function fitting neural network (FITNET)

## Abstract

In this paper, we report a study having as a main goal the obtaining of a method that can provide an accurate forecast of the residential electricity consumption, refining it up to the appliance level, using sensor recorded data, for residential smart homes complexes that use renewable energy sources as a part of their consumed electricity, overcoming the limitations of not having available historical meteorological data and the unwillingness of the contractor to acquire such data periodically in the future accurate short-term forecasts from a specialized institute due to the implied costs. In this purpose, we have developed a mixed artificial neural network (ANN) approach using both non-linear autoregressive with exogenous input (NARX) ANNs and function fitting neural networks (FITNETs). We have used a large dataset containing detailed electricity consumption data recorded by sensors, monitoring a series of individual appliances, while in the NARX case we have also used timestamps datasets as exogenous variables. After having developed and validated the forecasting method, we have compiled it in view of incorporating it into a cloud solution, being delivered to the contractor that can provide it as a service for a monthly fee to both the operators and residential consumers.

## 1. Introduction

The ambitious goal of the European Union (EU), to reduce energy consumption by 2050 by 80–95%, compared to 1990, has led to a series of proposals and measures concerning energy consumption reduction, diversification of the energy sources and smart usage of energy [1]. The 2050 Energy Strategy follows the 2020 version, initiated by the European Parliament [2], using the Strategy Energy Technology (SET) plan as line of action. The main measures foreseen by the 2017 updated version of the plan [3], envisage increasing the renewable energies sources quota in total energy production capacity, introduction of a consumer-centric energy business model, with a large scale adoption of Information Technology and Communications (IT&C) adoption, increased energy efficiency, by retrofitting buildings and households, regarding their external envelopes, but also for high consuming industry processes, capturing carbon, safely depositing or using it as a fuel, after further processing, and last, to increase the nuclear energy safety.

Looking at the statistics, the global energy production in 2016, according to [4], was 13,276 million tons of oil equivalent (Mtoe), with the following energy source distribution: oil 32%, coal 27%, gas 21%, biomass 11% and electricity 9%, where fossil sources still have an important share. Furthermore, the biggest world consumer, China, relies on coal (63%), as a primary source of energy production, that reached 3123 Mtoe. Regarding EU, the last data available [5], shows a production level of 1626.7 Mtoe in 2015, divided as consumption mainly between transport 33.1%, household 25.4% and industry 25.3%. The EU declares, in the same document, that 20% of the planned energy reduction has already been achieved.

However, the benefits of implementation of the SET plan, expressed in various assessments, suggest different possible lines of action. In [6], the authors emphasized an expected energy savings of up to 46%, obtained by means of thermal insulation of buildings and households. Regarding the largely IT&C use of energy delivery, in the context of smart cities and smart homes, in [7] a 4.5% energy consumption reduction and 7% for energy cost cuts was appreciated.

The concept of smart city covers a large variety of IT&C systems [8], oriented towards various sectors like energy efficiency, transportation and vehicular traffic, environmental pollution control, water systems, security and surveillance systems, weather, healthcare, governance and pools and social network and entertainment. Moreover, the energy efficiency horizontal refers to different subsystems: smart grids [9], smart lightning, smart metering, forecasting the energy consumption on a city scale or strategies for the development of city energy production facilities.

When deploying IT&C on a city scale, it is foreseen to gain multiple benefits, as highlighted by many case studies. In [10] a Big Data implementation on a city scale for the services described above was depicted. The overall system is based on the Internet of Things, used to collect various data, connected to cloud structures, which then are aggregated according to scope. Another case study is proposed in [11], where a solution for a smart city platform is presented. At the European level, the European Research Cluster on the Internet of Things (IERC) recommends a set of actions regarding implementation of the Internet of Things (IoT) and smart city initiatives [12]. Regarding the functionalities of data collection and management system regarding household consumption, in [13], the authors underline the necessity of energy consumption forecasting, exploring existing modeling techniques including classification and clustering algorithms. However, the optimization function is also debated, offering solutions like mathematical optimization (linear programming, dynamic programing), meta-heuristic optimization (evolutionary and genetic algorithms, particle swarm optimization) or heuristic optimization (ANN, Markov process). The study reveals details regarding Demand Side Management for appliances, using demand response, by scheduling the controlled appliance in time intervals, according to the different PV electricity production tariffs. The smart metering data classification algorithms are analyzed in more depth in [14]. Profiling the consumer behavior is also presented in [15], in the context of space heating or cooling [16]. A smart grid approach regarding PV and demand response implementation is presented in [17] as a literature review. During the last decades, many researchers worldwide have analyzed and developed forecasting methods for electricity consumption. Therefore, in [18], the authors aim to find a machine learning technique to determine the relationship between building occupancy and utilities consumption. Furthermore, in [19] the occupancy aspect has been studied in detail, where authors enhance an energy disaggregation method using ultra-wideband radar and electrical signature profiles. The load disaggregation is employed in [20], where the authors acknowledge a limitation of the proposed method arising from the fact that the required data collection interval of the method is up to 10 min while the legal metering interval in Norway is 1 h, therefore being impossible to detect the electrical signals of the appliances appropriately.

The load disaggregation is also approached in a book chapter [21], where the authors acknowledge the ever-growing importance in recent years of the nonintrusive load monitoring (NILM) technique that targets estimating from the household’s aggregate electricity consumption, the electricity consumption of certain appliances and its undisputable advantages when compared with other intrusive approaches. The authors first analyze various types of appliances within a household and afterwards they present state-of-the-art load signatures as well as their macroscopic and microscopic characteristics. In the book chapter, the most important algorithms that are used for the purpose of load disaggregation, datasets that are freely available to perform experiments, open-source software and many possible benefits of the load disaggregation are analyzed in detail.

Another application of load disaggregation is presented in [22], where the authors use this technique in order to isolate from the aggregate consumption of a smart meter, the air-conditioning load, at each time step, in view of obtaining a short-term prediction of this load by making use of historical weather data.

In reference [23] the authors develop a forecasting method based on ANNs without having used meteorological data, aiming to achieve a 24-h-ahead load forecasting of the aggregated consumption at the level of the entire country of Turkey. In addition, they have also used techniques such as wavelet transform and radial basis function neural networks; wavelet transform and artificial neural networks; empirical mode decomposition and radial basis function neural networks.

The authors of [24] propose an approach in view of developing a short-term hourly forecasting solution, with a time horizon ranging from a few hours up to 1 week, targeting the Greek power system load demand. For this purpose, they have developed several feed-forward artificial neural networks and they have concluded that a training set of 3 years provides the best forecasting results.

Using an ANN approach, the authors of [25] developed a day ahead load forecasting method in the case of the power system in Turkey, taking into account the load variation caused by national or religious holidays. In order to evaluate the obtained performance, the authors take into account the mean absolute percentage error and the hourly absolute percentage error.

In [26], the authors have used the Levenberg-Marquardt training algorithm in order to develop ANNs based on the NAR and NARX models. The authors have targeted in their forecast a set of public buildings over a period of 100 days, using a single prediction data point per day for the aggregated consumption of the respective building. In developing the ANNs, they have tested different settings, by adjusting the network parameters, in order to identify the setting that offers the best forecasting results. The authors concluded that they obtained very good results in terms of forecasting accuracy.

In [27], the authors propose a method based on Deep Neural Networks using two versions of the Long Short-Term Memory (LSTM) algorithms: a standard version and a version that employs a Sequence to Sequence architecture. The authors state that the results obtained through their methods are comparable with the ones obtained using other deep learning methods targeting energy forecasting and conclude that they intend to use their developed solution on real world datasets in order to assess in a reliable way the efficiency of their proposed solution.

The paper [28] presents a prediction model of the residential electricity demand in provincial China, using for this purpose the reduced form of a linear model in order to model the influence that the income has on the consumption of electricity at the residential level. Based on this study, the authors conclude that the developed provinces will experience a decline regarding the growth rate of the electricity demand, and the other provinces will experience growth at a much faster pace.

The authors of [29] have developed an implementation of the NARX model in order to forecast the electric loads in a grid, using the New England electrical load data for the training and validation processes, while the timestamps and meteorological data were used as exogenous variables. They have obtained a 24-h ahead load forecasting, resulting in an hourly dataset. The authors claim that by applying this method they have obtained an improvement of 30% regarding the average error when using feedforward artificial neural networks instead of state space methods and autoregressive-moving average with exogenous terms (ARMAX) model, consequently obtaining large savings that could potentially prevent commissioning of some power plants.

Reviewing the scientific literature and analyzing the current state of art, one can notice that undisputable advances that have been made from the perspective of Information and Communication Technologies (ICTs) that facilitate interoperability down to the appliance level of a household, with appliances that nowadays can be linked together and monitored using advanced sensors and measuring instruments, facilitating the obtaining of more and more detailed information that makes it possible to offer residential consumers new prospects for services that until recently were unattainable.

In the context of the latest breakthroughs in sensor technologies and artificial intelligence algorithms, the modern smart home environment has evolved up to a point where it can oversee and improve the quality of life for its residents, to gain in-depth knowledge of their individual behaviors and adjust in accordance with their everyday life. In order to achieve this, smart homes must be able to manage as efficiently as possible the energy usage at the appliance level, therefore providing an energy-efficient environment for its residents that has the potential to assure and improve their overall being. In this context, being able to forecast the electricity consumption at the appliance level with a high degree of accuracy becomes a very important aspect in the smart home environment of today.

The main aim of our paper is to develop and validate a method that is able to accurately forecast the residential electricity consumption down to the appliance level, using sensor recorded data, for residential smart home complexes that utilize renewable energy sources as a part of their consumed electricity. In order to attain this goal, we have developed a mixed artificial neural network (ANN) approach using both non-linear autoregressive with exogenous input (NARX) ANNs and function fitting neural networks (FITNETs). In this purpose, we have used a large dataset containing detailed electricity consumption data recorded by the sensors, monitoring a series of individual appliances, while in the NARX case we have also used timestamp datasets as exogenous variables. By testing various network architectures and forecasting scenarios, we have identified the NARX-FITNET mix that provided the best forecasting results.

Our article develops a new approach that is linked with the progress of technologies (smart metering, sensors, and actuators) that allow detailed electricity consumption analytics. Nowadays, it is not sufficient to know only the monthly consumption of individual electricity consumers or communities, being more and more necessary to acquire hourly consumption data that can reveal the consumption characteristics of each consumer in order to design the load profile, adequate advanced tariff schemes, monitor, optimize the consumption and identify the energy intensive appliances that can be further replaced to meet energy efficiency targets. Therefore, when such detailed data is available (and there will be more and more available data in the near future as a consequence of the EU strategies), it is recommended to collect, process and use them by extracting valuable information.

The main challenge that we had to overcome in our research consists in developing a method targeting the forecasting of the residential electricity consumption, namely the total electricity consumption coming from the grid, the total electricity consumption of all the individual appliances, refining the forecast up to the electricity consumption at the appliance level for residential smart homes complexes whose houses use renewable energy sources to sustain a part of their consumed electricity, surpassing the limitations of not having historical meteorological data and unwillingness of the contractor to acquire in the future accurate short-term meteorological forecasts from a specialized institute due to the implied costs and deploy the method in a IoT cloud solution with the purpose of delivering it as a service to selected customers for a monthly fee. Although being at an incipient phase and having limitations before it can become a profitable solution in the near future, we consider that the prospects of improving and evolving the method after having put it in a real use environment are promising.

The rest of the paper is organized as follows: Section 2 (Materials and Methods), presents the forecasting method, its stages and steps, providing details about acquiring and processing the data collected from the sensors, developing the non-linear autoregressive with exogenous inputs (NARX) artificial neural network (ANN) forecasting solution for the total electricity consumption coming from the grid (case 1) and the total electricity consumption of all the individual appliances (case 2), developing the function fitting neural network (FITNET) forecasting solution for the electricity consumption dataset for each of the individual appliances (stemming from the total electricity consumption coming from the grid in the first case and similarly, stemming from the total electricity consumption of all the individual appliances, in the second case), obtaining the forecast using the best mix of NARX and FITNET ANNs (in both cases) in view of validating the forecasting solution, compiling the developed method in view of incorporating it into the designed cloud solution. Afterwards, Section 3 describes the results regarding the developed ANN forecasting solution based on the NARX model, the developed FITNET ANN forecasting solution and the validation of the devised method. The Discussion presented in Section 4 analyzes the results, interpreting them in perspective of previous studies and of the working hypotheses, highlighting also future research directions. Finally, Section 5 outstands the main obtained conclusions.

## 2. Materials and Methods

When devising the research methodology, we wanted to verify if the method that we have developed and applied in [30] for commercial center type consumers can be successfully applied in the case of household consumers to have the electricity consumptions recorded for most of the individual appliances. The method that we have proposed in [30] comprises five stages and is based on developing artificial neural network (ANN) forecasting solutions based on the nonlinear autoregressive (NAR) model, ANNs based on the non-linear autoregressive with exogenous inputs (NARX) model using as exogenous variables a meteorological and timestamps dataset and ANNs based on the NARX model that make use for the exogenous variables only of a timestamps dataset.

Therefore, we have first tried to develop artificial neural networks based on the nonlinear autoregressive model so as to forecast the individual electricity consumption for each of the appliances. After having performed a series of experimental tests, varying the number of hidden neurons over a wide range, we recorded performance metrics that depicted a low level of forecasting accuracy. We decided that the forecasting ANNs based on the NAR model were not a viable solution in this case and we moved on trying to achieve an improved forecasting by developing ANNs based on the NARX model.

We had to make adjustments to how the timestamps dataset was built in the case of the household consumer as the time series contained a supplementary information regarding the minute of the hour, the data being recorded by the sensors every 15 min. Therefore, in the case of the residential consumer method the timestamps dataset contains five series of data, namely: the hour of the day, the minute of the hour, the day of the week, the day of the month, the month of the year. In the case of the household consumer, we did not have access to the meteorological data recorded every 15 min (or at least hourly) to be able to train an ANN based on the NARX model that uses among the exogenous variables the meteorological data. Even if we had had an appropriate meteorological dataset available for the training process, we wouldn’t have been able to predict future values using the trained network if we had not had accurately forecasted meteorological data from a specialized institute every 15 min at a refined resolution for the desired forecast timeframe.

Consequently, in this point, we have tried to devise the method relying on forecasting ANNs based on the NARX model, using as time series the individual electricity consumptions for each of the appliances (the forecasting target of our article) and as exogenous variables the timestamps dataset depicted above. Having access from the smart meter to the total electricity consumption that is drawn from the electricity grid, we have also tried to use this information as an exogenous variable along with the timestamps dataset. However, the problem consisted in the fact that the smart home had the possibility to self-sustain a part of its electricity consumption, being equipped with a solar panel system and battery storage.

Therefore, the total consumption from the electricity grid did not help improve the prediction accuracy when compared to using only the timestamps dataset, because we did not know if the recorded consumption for a certain appliance came from the electricity grid or from the solar panel system. After having run a series of comprehensive experimental tests, varying the number of neurons from the hidden layer and the delay parameter, we noticed that the performance metrics, although drastically improved from the NAR’s performance metrics due to the addition of the timestamps dataset, still depicted an unsatisfactory level of accuracy for the forecasting of the individual consumption of the monitored appliances.

After we have made numerous attempts to devise a viable method for our purpose, testing different types of artificial neural networks architectures, we remarked that FITNET ANNs are able to predict with good forecasting accuracy the individual electricity consumption of the appliances when the FITNET ANNs are provided as inputs the total electricity consumption coming from the grid along with the moment of time when it was recorded (the timestamps dataset).

As we wanted to see if we could further improve the prediction accuracy, we have devised and tested a second approach in which the neural networks are provided as inputs the total electricity consumption of all the appliances and the timestamps dataset. Through this approach, we have managed to improve even further the forecasting accuracy and overcome the abovementioned limitation of not knowing if the recorded consumption of a certain appliance came from the electricity grid, from the solar panel system or different parts of the consumption came from different sources (electricity grid or solar panel system).

Consequently, we have studied if an artificial neural network based on the NARX model has the potential to provide an accurate prediction of the total electricity consumption coming from the grid and the total electricity consumption of all the monitored appliances. After extensive testing, we have succeeded in developing ANNs based on the NARX model that use as exogenous variables the timestamps dataset and provide forecasts with a high level of accuracy for the desired purpose.

Therefore, we have devised our forecasting method by structuring it into five consecutive stages. The first stage of the method comprises five steps, consisting in collecting and processing the data recorded by a smart metering system and by the sensors.

During the second stage of the method, consisting in four steps, we have developed an artificial neural network forecasting solution based on the non-linear autoregressive with exogenous inputs model, in order to forecast, in the first case, the total electricity consumption from the grid, and in the second case the total electricity consumption of all the individual appliances dataset, using as exogenous variables the timestamps dataset in both cases, the datasets having being constructed during the first stage of the method.

The third stage of the method obtains the electricity consumption dataset for the appliances of the household during four steps, by developing a series of FITNET artificial neural networks that use as input values the previously developed timestamps dataset along with the total electricity consumption from the grid in the first case, and along with the total electricity consumption of all the appliances in the second case.

In the fourth stage of the devised method, we have obtained the best forecasting solution for all the individual appliances of the residential electricity consumer during four steps, by using the best mix of NARX and FITNET ANNs, in view of validating the forecasting solution of all the individual appliances electricity consumption stemming from the total electricity consumption from the grid along with the second quarter-hourly subset of the timestamps dataset in the first case, and the total electricity consumption of all the individual appliances along with the second quarter-hourly subset of the timestamps dataset, in the second one.

In the fifth stage of our method, we have compiled the devised forecasting method in view of incorporating it into our designed cloud solution, being delivered to the contractor and ready to be used in a production environment.

**Figure 1:**
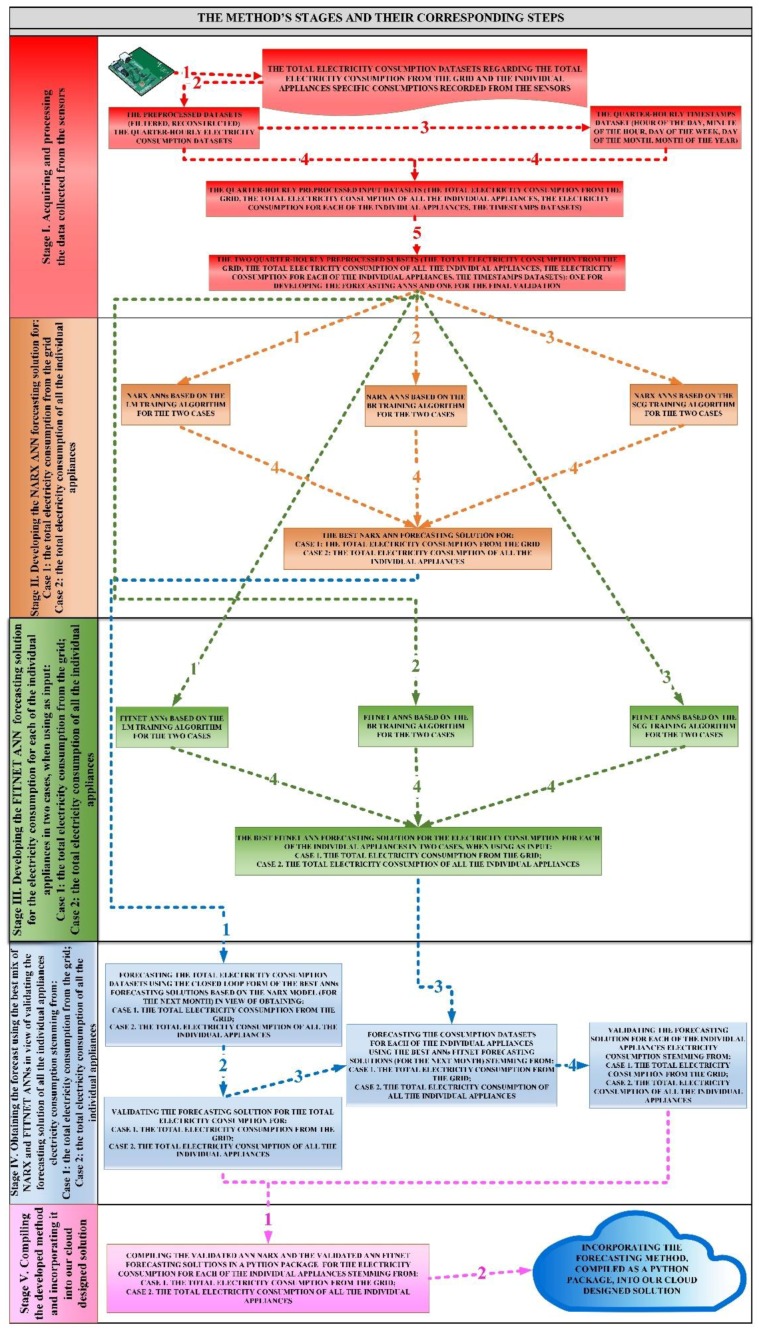
The block diagram of the devised forecasting method.

The mixed neural network approach developed in order to forecast the residential electricity consumption based on sensors recorded data, is synthesized in the block diagram, in which we have depicted the five steps of the first stage, the four steps of the second one, the four steps of the third stage, the four steps of the fourth one, the two steps of the fifth stage, highlighting the relationships between them, the logical flow and their sequence (Figure 1). An overview of the method’s stages and steps can be found in the Supplementary Materials file (Table S1).

We have applied the devised method to a series of eight smart homes equipped with renewable electricity generation systems, smart homes that have the total consumption recorded by a smart metering system and the consumption of different appliances monitored using the sensors described in the following subsection and we have obtained results with a good forecasting accuracy.

In what follows, we present an eloquent but non-limitative case where the devised method is applied to one of smart homes (Smart Home 3) of the eight studied cases, by describing in detail the activities performed during each of the stages and their corresponding steps. We would like to mention that this case study did not provide the best forecasting results, as it was the fifth in the hierarchy regarding the prediction accuracy, but the differences between this case and the other seven ones are insignificant, the performance parameters varying only slightly.

### 2.1. Acquiring and Processing the Data Collected from the Sensors

**Figure 2:**
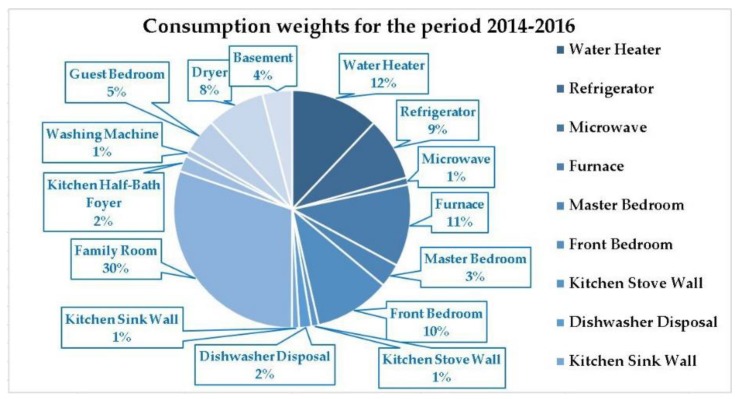
Consumption weights for the period 2014–2016.

The first stage of the devised method consists in acquiring and processing the data collected from the sensors, comprising five steps. During the first step of the first stage, we have acquired a large dataset containing the total electricity consumption coming from the grid and detailed electricity consumption datasets (measured in kWh) recorded every 15 min for a three year period (1 January 2014–31 December 2016) by the sensors installed at a residential consumer, corresponding to 15 individual appliances (water heater, refrigerator, microwave, furnace, master bedroom, front bedroom, kitchen stove wall, dishwasher disposal, kitchen sink wall, family room, kitchen half-bath foyer, washing machine, guest bedroom, dryer, basement). Each of these datasets comprises a number of 105,216 samples. The weights of the appliances are presented in Figure 2.

**Figure 3:**
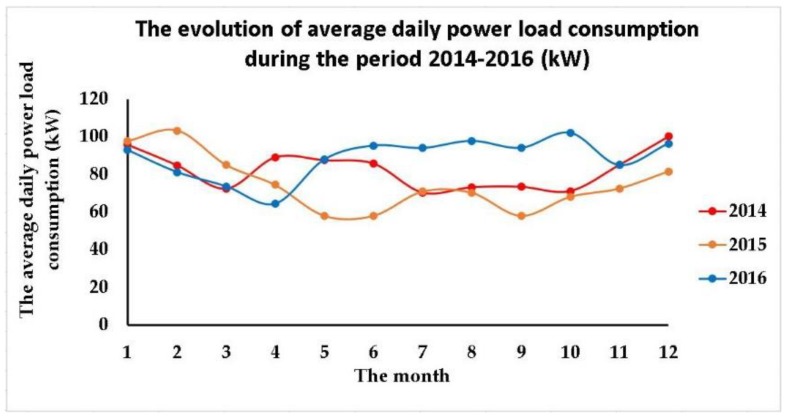
The evolution of average daily power load consumption per month.

The highest four consumptions are family room appliances 30%, water heater 12%, furnace 11% and front bedroom appliances 10%. In order to facilitate the analysis of the data subjected to the modelling process, we have first filtered and grouped the data in annual time series for the period 2014–2016, and within each annual series we have summed the values of the power load consumption of all the appliances per month, computing then the average daily power load consumption per month. The graphical representation of the annual series regarding the average daily power load consumption for the period 2014–2016, in conjunction with the analysis of the main descriptive statistics highlighting the behavior of the analyzed process, reveal the presence of undulatory evolutions over periods of less than a year, indicating the influence of some factors that determine certain seasonal variations to the process. Due to these similarities, but also to the opportunity of having access to a large amount of data, we have considered more relevant to model the electricity consumption throughout the whole observed period of three years (Figure 3).

As the data recorded by the sensors can sometimes be susceptible to errors, therefore causing abnormal or even missing values to be retrieved, in the second step of the first stage, in order to reconstruct the dataset, to obtain a consistent quarter-hourly electricity consumption dataset that will be used in the subsequent steps and stages of the method, we have preprocessed, filtered and applied a gap-filling technique based on the linear interpolation that we have previously developed and applied successfully in [30] for the missing or abnormal values regarding the electricity consumption datasets in the case of commercial center type consumers. The linear interpolation technique is suitable for our research as the involved data is discreet and, due to their collection method, there were very few missing data points regarding the electricity consumption of the appliances.

**Figure 4:**
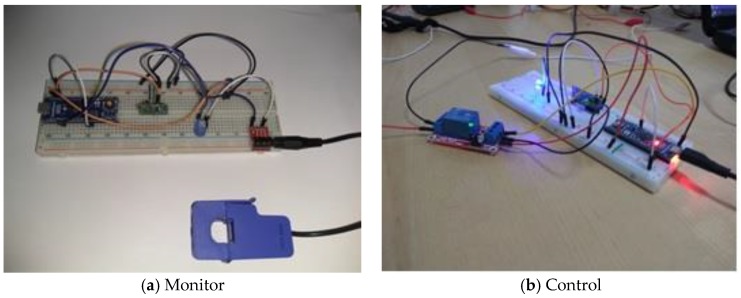
The transmitter breadboards for monitor and control.

The proposed solution for controlling and monitoring the appliances consists in components for transmission and receiving data between each appliance and the main gateway based on Arduino components assembled on two breadboards: transmitter and receiver. For the transmitter (T) we have used an Arduino Micro based on an ATmega32U4 microcontroller with 20 digital pins used for input/output operations. It contains a crystal oscillator, an automatic voltage regulator (AVR), a microUSB connector allowing the power supply of the controller, a serial circuit programming or In-Circuit Serial Programming (ICSP) that provides the facility to be programmed while being part of a complete system, instead of programming it before assembling. For measuring the electricity consumption of the appliances, we have connected to the Arduino Micro an YHDC SCT-013 current transformer based on a non-invasive AC current sensor (100A max). The connection is made through a standard 3.5 mm stereo audio jack. To avoid overloading and burning the Arduino Micro, a 220 μF capacitor and two 330 Ohm resistors are used. In case of power failure, the Arduino Micro is supplied by an external battery with a capacity of 2600 mAh connected to the microUSB charger. For controlling programmable appliances, a relay with an optocoupler is added to the Arduino Micro breadboard. The transmitter breadboards for monitoring the non-programmable appliances (a) and controlling programmable appliances (b) are presented in Figure 4.

**Figure 5:**
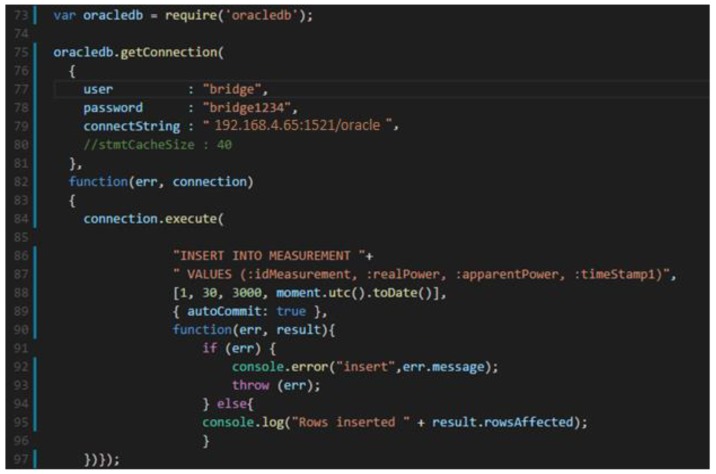
Node.js application for inserting records into the Oracle Database.

In case of the appliances described in Section 2.1, we set up a T controlling component with relay for each programmable appliance and a T monitoring component for non-programmable appliance. For receiver (*R*), a Raspberry Pi is used, and it communicates with the transmitter though RF 433 MHz transmitter-receiver modules. The transmitter sends data to receiver that acts as a gateway. The T-R kit is controlled through an application developed in Node.js. Since Node.js doesn’t buffer the data, the application is passing data into pieces and inserting the records into an Oracle Database (Figure 5).

A function is used to set the connection with the Oracle Database that calls connection.execute routines to insert the records from the receiver into a database table called Measurement, used to store the appliances’ consumption.

Based on the time moments when the 105,216 samples comprising the detailed electricity consumption datasets have been registered, in the third step of the first stage we have built a quarter-hourly timestamps dataset, containing the same number of samples and data regarding the hour of the day (ranging from 1 to 24), the minute of the hour (recorded from 15 to 15 min, namely 0, 15, 30, 45), the day of the week (ranging from 1 to 7, where 1 corresponds to Sunday), the day of the month (ranging from 1 to 28, 29, 30 or 31, according to the number of days of the respective month) and the month of the year (ranging from 1 to 12, where 1 corresponds to the month of January). The construction method of the timestamps dataset ensures the fact that this set is not susceptible to be inconsistently, error-prone or wrongly measured.

Afterwards, in the fourth step, using the preprocessed quarter-hourly electricity consumption datasets from step 2, we have computed the total electricity consumption of all the individual appliances and thus, at the end of this step, the whole quarter-hourly preprocessed input datasets have been obtained (the total electricity consumption coming from the grid, the total electricity consumption of all the individual appliances, the electricity consumption for each of the individual appliances, the timestamps datasets).

During the last step of the first stage, we have divided each of the datasets (the total electricity consumption coming from the grid, the total electricity consumption of all the individual appliances, the electricity consumption for each of the individual appliances and the timestamps), into two subsets. The first subset corresponds to the period 1 January 2014–30 November 2016, contains 102,240 samples and it will be used in the subsequent stages in order to train the forecasting ANNs, while the second subset comprises 2976 samples, corresponding to the last month of the year 2016 and it will be used later in order to obtain a final validation of the developed forecasting solution, through a comparison between the forecasted values for the month of December 2016 and the real ones, from this second data subset.

Consequently, at the end of the first stage, we have obtained two quarter-hourly preprocessed subsets (the total electricity consumption from the grid, the total electricity consumption of all the individual appliances, the electricity consumption for each of the individual appliances, the timestamps datasets): one for developing the forecasting ANNs and one for the final validation of the developed forecasting solution.

In the next two stages of the forecasting method we have developed the ANNs forecasting solutions, based on the NARX and FITNET models. We have developed the NARX ANN forecasting solution covering two cases, the first one for the total electricity consumption from the grid and the second one for the total electricity consumption of all the individual appliances. As for the FITNET ANN forecasting solution for the electricity consumption for each of the individual appliances, we have developed it addressing two cases, using as input in the first case the total electricity consumption from the grid along with the timestamps dataset and in the second one the total electricity consumption of all the individual appliances along with the timestamps dataset. In what concerns the ANNs developed based on the NARX model during the second stage of our method, in order to improve the efficiency of the training process of the networks, we have trained, validated and tested the ANNs in the open loop form. Consequently, we have ensured that in the development process of the artificial neural networks they are provided with the real past values and thus, the current results are correctly generated by the ANNs.

After having finished the artificial neural networks training, validation and testing, we have put the NARX ANNs into the closed loop form, so they are able to perform a multi-step ahead prediction [30]. Otherwise, if the networks had not been put into the closed loop form, we would not have been able to forecast more than one step ahead using the past values of the time series and of the exogenous variables. By configuring the network into the closed loop form, one can make predictions for more steps ahead as besides the recorded values of the time series, the network can also use the forecasted values from the previous steps.

### 2.2. Developing the NARX ANN Forecasting Solution for the Total Electricity Consumption Coming from the Grid and the Total Electricity Consumption of All the Individual Appliances

The second stage of our method comprises four steps and consists in developing the NARX ANN forecasting solution for the total electricity consumption coming from the grid and the total electricity consumption of all the individual appliances. In this purpose, we have used data from the first quarter-hourly subset constructed during the first stage. Therefore, in the first case, we have used the total electricity consumption coming from the grid as the timeseries and the timestamps dataset as exogenous variables, while in the second case we have used the total electricity consumption of all the individual appliances dataset as the timeseries and the timestamps dataset as exogenous variables. In both cases, in order to obtain the best forecasting solution, we have researched various settings in developing the networks, regarding the training algorithm (Levenberg-Marquardt, Bayesian Regularization, Scaled Conjugate Gradient), the hidden number of neurons and the delay parameter. We have thus obtained the optimal mix between these settings and saved it in order to further use the corresponding NARX ANN in forecasting the total electricity consumption coming from the grid (in the first case) and the total electricity consumption of all the individual appliances (in the second case).

Many phenomena and problems are modeled using time series having a dynamic and non-linear behavior, as each of their state depends on previous ones [26]. In these cases, the non-linear autoregressive (NAR) model might help in forecasting the actual value of a time series based only on previous values of the same series.

In contrast to this model, in the case of the non-linear autoregressive with exogenous inputs (NARX) model, the time series forecasting model relates the actual value of the time series both to previous values of the series and to additional external time series (namely the exogenous data). This is possible in cases where the time series that has to be predicted is influenced and correlated with another one (or more). For example, in our research, the forecasting of the total electricity consumption coming from the grid and the one of all the individual appliances are in the above-mentioned situation as the data involved in our study is represented by discrete, non-linear time series that is obviously correlated with the timestamps dataset.

The mathematical formalization of the nonlinear autoregressive with exogenous inputs (NARX) model is based on the equation:

(1) y(t)=F(y(t−1),…,y(t−d),x(t),x(t−1),…,x(t−d))+ϵ(t)

stating that the actual value of the time series of interest y(t), that has to be forecasted by the artificial neural network, depends on d of the previous values of the series (d∈ℕ, d≥1 is the delay parameter) and also on the actual and the previous d values of another time series, the exogenous one, denoted by x(t). The term ϵ(t) from the right side of the Equation (1), represents the approximation error of the actual value of the time series y(t).

In order to obtain the forecasting, one should approximate with the highest level of accuracy the nonlinear function F from the right side of the Equation (1) by testing various settings of the artificial neural network: the number of neurons in each layer, the number of hidden layers, the network’s weights and biases [30]. Based on this method one obtains the settings of the ANN that has the ability to provide the best forecasting accuracy.

A very important fact that must be taken into consideration when using this approach is the fact that the variation of the number of neurons should be carried out with caution: lowering this number too much could have the effect of reducing the neural network’s computational power and restricting its capability of generalization, while using a higher number than needed might increase the complexity of the system.

The Levenberg-Marquardt (LM) training algorithm [31,32,33] combines the performance and features of two optimization methods, namely the gradient descent and the Gauss-Newton one and offers to the programmers and researchers multiple undeniable advantages. Therefore, in the first step of the second stage we have decided to develop a number of 24 artificial neural networks based on the Levenberg-Marquardt (LM) training algorithm and on the non-linear autoregressive with exogenous inputs (NARX) model, 12 neural networks being developed in view of devising a forecasting solution for the total electricity consumption coming from the grid and the other 12 ANNs for predicting the total electricity consumption of all the individual appliances.

In developing the forecasting ANNs we have used various settings in order to identify the best NARX ANN forecasting solution for the total electricity consumption coming from the grid and the one for all the individual appliances, based on the LM training algorithm. Thus, we have used six neurons for the input data (one neuron for the total electricity consumption coming from the grid, one neuron for the total electricity consumption of all the individual appliances dataset and five neurons for the timestamps exogenous data, respectively), n neurons in the hidden layer, where n∈{6, 12, 24} and the delay parameter d∈{8, 16, 24, 48}, one neuron for the output layer and one neuron for the output data (measured in kWh).

For all the datasets we have used the first subset containing 102,240 samples. In developing the ANNs we have divided the input dataset by allocating 70% of it for the training process, 15% for the validation one and the remaining 15% for the testing process, each of these percentages being composed by randomly chosen samples. In order to minimize the influence of the data ranges variations on the final forecasted results, we have set the standard value for the normalization performance parameter. In this manner, we have set the development environment to consider the output elements between −1 and 1 and to compute the errors accordingly.

In developing an artificial neural network, one should pay special attention to the most important factor implied in converting the input in such a manner as to influence the output: the weights, representing numerical parameters that reflect the way how each of the neurons affects the others. Each neuron’s output represents a sum of all the products of the weights and inputs and an additional parameter, the bias that can be used to adjust the neuron’s output [30].

Therefore, another important issue that we have taken into consideration when developing the ANNs based on the Levenberg-Marquardt (LM) training algorithm and on the non-linear autoregressive with exogenous inputs (NARX) model is the fact that the networks’ parameters (weights and biases) are re-initialized when the data within the above-mentioned percentages are randomly re-allocated.

In order to identify and save the network that offered the best forecasting accuracy, for each of the 24 above-mentioned developed ANNs (corresponding to both cases and to all the possible pairs (n,d), where n∈{6, 12, 24} and d∈{8, 16, 24, 48}), we have run 10 iterations, generating performance plots highlighted by the mean squared error, the error histogram, the regressions, the error autocorrelation. Comparing these plots, we have identified and saved for each of the cases the network characterized by the best forecasting accuracy out of the 10 ones, having the same number of neurons in the hidden layer and the same delay parameter, obtaining thus a total number of 24 forecasting artificial neural networks, trained using the LM algorithm and developed based on the NARX model.

During the second step of the second stage we have used the Bayesian Regularization (BR) training algorithm in order to develop a number of 24 artificial neural networks based on the NARX model, 12 of them being designed to forecast the total electricity consumption coming from the grid and the remaining 12 to forecast the total electricity consumption of all the individual appliances. The BR algorithm is based on the LM and on the backward propagation algorithms [30,31,34,35].

In contrast to other training algorithms, the Bayesian Regularization algorithm offers as a main advantage the fact that it does not require a validation step, avoiding thus the necessity of reserving data for validation purposes and therefore, the costs of this process. During the years, the BR training algorithm has proved to be an efficient tool in the development of the artificial neural networks and consequently we have decided to analyze the performance of the ANNs developed based on this algorithm and on the NARX model in forecasting the total electricity consumption coming from the grid and the total electricity consumption of all the individual appliances.

We have used a similar method as in the case of the LM training algorithm when developing the forecasting ANNs based on the BR training algorithm, taking into consideration that the validation process does not occur in this case. Consequently, when allocating the data (70% for training, 15% for the testing one) the rest of 15% had remained unallocated. We have decided to implement this allocation strategy in order to obtain the same amount of data in the training and testing processes for both the LM and BR algorithms, thus creating the premises of obtaining afterwards a relevant comparison between the results provided by the two forecasting ANNs, trained using the two algorithms.

As in the case of the LM training algorithm, the samples that compose the abovementioned percentages have been chosen randomly and the output data has been normalized. We have used the same approach as in the LM case in testing various settings regarding the number of neurons in the hidden layer and the delay parameter, running for each of the cases 10 iterations and saving the ANN that has provided the best forecasting accuracy (highlighted by the performance plots), obtaining thus 24 forecasting artificial neural networks, trained using the BR algorithm and developed based on the NARX model.

Another algorithm that has proved its usefulness during the years in developing artificial forecasting neural networks is the Scaled Conjugate Gradient (SCG) training algorithm, that combines the Levenberg-Marquardt’s model-trust region approach with the conjugate gradient one. Considering its undeniable advantages [33,36] we have chosen to analyze if this algorithm is suitable in developing NARX ANN forecasting solutions for the total electricity consumption coming from the grid and the total electricity consumption of all the individual appliances. In developing the forecasting ANNs, we have used the same approach as in the case of the LM training algorithm in what concerns the data allocation percentages, the normalization of the output data, the execution of 10 iterations per each case in order to identify the (n,d) pair that leads to the ANN that offers the best forecasting results, obtaining thus a number of 24 forecasting ANNs, developed based on the NARX model and trained using the SCG algorithm.

During the final step of the second stage, we have compared for each of the two cases the forecasting accuracy of all the ANNs, trained using the LM, BR and SCG algorithms, obtained by processing the steps 1–3 of the second stage. By analyzing the performance metrics of the 36 NARX ANNs developed in order to forecast the total electricity consumption coming from the grid, we have obtained the best NARX ANN forecasting solution in this case. Similarly, we have identified the best forecasting solution for the case of the total electricity consumption of all the individual appliances.

### 2.3. Developing the FITNET ANN Forecasting Solution for the Electricity Consumption Dataset for Each of the Individual Appliances

During the third stage of our method, comprising four steps, we have developed a function fitting neural networks (FITNET) artificial neural network forecasting solution for the electricity consumption dataset for each of the individual appliances in two cases, as follows: in the first case we have used as input the total electricity consumption coming from the grid along with the timestamps dataset, while in the second one we have used as input the total electricity consumption of all the individual appliances along with the timestamps dataset. The function fitting training process of an ANN is used in those cases when a set of inputs and outputs are known, and one develops an ANN that creates an association between the inputs and their corresponding outputs, after the training process being able to obtain outputs based on new inputs, that were not even in the training set.

In the training process of these networks, we have used data from the first quarter-hourly subset constructed in the fifth step of the first stage, as follows: in the first case, the total electricity consumption coming from the grid and the timestamps dataset as inputs, while in the second case the inputs were represented by the total electricity consumption of all the individual appliances and the timestamps dataset. In both cases, the output is the same and consists in the electricity consumption dataset for each of the individual appliances.

Similar to the previous stage, of particular interest was to research and identify the training algorithm (Levenberg-Marquardt, Bayesian Regularization, Scaled Conjugate Gradient) and the network architecture that have provided the best forecasting accuracy.

For each of the LM, BR and SCG training algorithms, we have divided the input dataset by allocating the same percentages and the same approach as in the case of the NARX ANNs development steps, the same normalization procedure for the output data, a similar testing methodology for the number of neurons in the hidden layer, a similar approach regarding the number of iterations per each considered case.

Thus, in both of the cases, we have tested various architectures, comprising six neurons for the input data (one neuron for the total electricity consumption coming from the grid, one neuron for the total electricity consumption of all the individual appliances dataset and five neurons for the timestamps dataset, respectively), n neurons in the hidden layer, where n∈{1,…,45}, 15 neurons for the output layer and 15 for the output data (the forecasted electricity consumption dataset for each of the 15 individual appliances, measured in kWh).

Therefore, using as input the total electricity consumption coming from the grid dataset, during the steps 1–3 of the third stage, we have obtained 45 forecasting FITNET artificial neural networks, developed using each of the training algorithms, resulting in a total number of 135 ANNs and the same number of FITNET ANNs using as input the total electricity consumption of all the individual appliances dataset.

In the final step of the third stage, we have compared the forecasting accuracy of all the FITNET ANNs, trained using the LM, BR and SCG algorithms, obtained during the previous steps, in each of the considered cases. After having analyzed the performance metrics of the developed FITNET ANNs, we have identified the best FITNET ANN forecasting solution for the electricity consumption dataset for each of the individual appliances, in the two cases: stemming from the total electricity consumption coming from the grid in the first case and similarly, stemming from the total electricity consumption of all the individual appliances, in the second case.

### 2.4. Obtaining the Forecast Using the Best Mix of NARX and FITNET ANNs in View of Validating the Forecasting Solution

In the fourth stage of our devised method four steps are processed as to obtain the forecast using the best mix of NARX and FITNET ANNs in view of validating the forecasting solution of all the individual appliances electricity consumption stemming from the total electricity consumption from the grid (in the first case) and from the total electricity consumption of all the individual appliances (in the second one). In the first step of this stage, we have forecasted using the closed loop form of the best ANN forecasting solution based on the NARX model for the next month the total electricity consumption of all the individual appliances stemming from the total electricity consumption from the grid (in the first case) and from the total electricity consumption of all the individual appliances (in the second one), considering the technical details that we have presented in Section 2.1. In this purpose, in both of the cases, we have used as input the data from the second quarter-hourly subset constructed in the first stage, using as exogenous variables the timestamps dataset for the next month and obtaining as an output the total forecasted electricity consumption of all the individual appliances dataset for the next month consisting in 2976 samples of one value.

During the second step of the fourth stage, we have validated the forecasting solution for the total electricity consumption of all the individual appliances stemming from the total electricity consumption from the grid (in the first case) and from the total electricity consumption of all the individual appliances (in the second one), by comparing in each of the two cases the total forecasted electricity consumption of all the individual appliances dataset obtained as output in the first step with the total electricity consumption of all the individual appliances dataset from the second quarter-hourly subset obtained at the end of the first stage.

In the third step of the fourth stage, we have forecast the consumption dataset for each of the individual appliances for the next month using the best ANN FITNET forecasting solution stemming from the total electricity consumption from the grid (in the first case) and from the total electricity consumption of all the individual appliances (in the second one). In order to achieve this, we have used in the first case as inputs for the trained FITNET ANN the total electricity consumption from the grid and the timestamps dataset from the second quarter-hourly subset obtained in the first stage, for the next month. In the second case, we have used as inputs the total electricity consumption of all the individual appliances and the timestamps dataset. The trained FITNET ANNs output in both cases the forecasted electricity consumption dataset for each of the individual appliances for the next month, stemming from the total electricity consumption from the grid (in the first case) and from the total electricity consumption of all the individual appliances (in the second one), consisting in each case in 2976 forecasted samples of 15 values (corresponding to each of the 15 monitored individual appliances) resulting in a total number of 44,640 values.

During the fourth step of the fourth stage, in order to obtain a final validation of the obtained forecasting solution for each of the individual appliances electricity consumption, after predicting the electricity consumption dataset for each of the individual appliances stemming from the total electricity consumption from the grid (in the first case) and from the total electricity consumption of all the individual appliances (in the second one), we have compared the obtained results with the corresponding real values of the consumption dataset for each of the individual appliances, values contained in the second quarter-hourly subset obtained at the end of the first stage of our devised method.

Using this approach, at the end of the fourth stage, we have obtained a valuable confirmation of the fact that our devised method based on a mix of NARX and FITNET ANNs is able to forecast with a high degree of accuracy the electricity consumption for each of the individual appliances stemming from the total electricity consumption from the grid (in the first case) and from the total electricity consumption of all the individual appliances (in the second one), obtaining thus the validation of our forecasting solution.

### 2.5. Compiling the Developed Method and Incorporating It into the Designed Cloud Solution

In the fifth stage two steps are processed in view of compiling the devised forecasting method and incorporating it into our cloud developed solution. Therefore, in the first step of this stage, we have used the MATLAB Compiler Software Development Kit (SDK) in order to compile both the validated ANN NARX forecasting solution for the total electricity consumption of all the individual appliances and the validated ANN FITNET forecasting solution for the electricity consumption dataset for each of the individual appliances stemming from the total electricity consumption from the grid (in the first case) and from the total electricity consumption of all the individual appliances (in the second one) in a Python package.

Afterwards, during the second step of the stage, we will incorporate the forecasting method, compiled as a Python package into our cloud designed solution, thus achieving the integration of the developed compiled forecasting method for the individual appliances’ electricity consumption of residential consumers based on sensors recorded data. In the following, we present the results obtained after having run the experimental tests.

## 3. Results

When developing the mixed neural network approach in view of forecasting the individual appliances electricity consumption of residential consumers based on sensors recorded data, we have used a system having the following hardware and software configuration: the Central Processing Unit (CPU) Intel i7-5960x from the Haswell family, having a clock frequency of 3.0 GHz, with the standard Turbo Boost feature enabled, the system being equipped with 32 GB Double Data Rate Fourth-Generation (DDR4) Synchronous Dynamic Random-Access Memory (SDRAM), operating in quad channel mode, at a frequency of 2144 MHz; the Graphics Processing Unit (GPU) GeForce GTX 1080 TI NVIDIA based on the Pascal architecture, having a memory of 11 GB of Double Data Rate Type Five Synchronous Graphics Random-Access Memory GDDR5X with a bandwidth of 352-bit; the Windows 10 Redstone 2 Educational Version (10.0.15063.296) operating system; the MATLAB R2017a development environment.

### 3.1. Results Regarding the Developed ANN Forecasting Solution Based on the NARX Model

In accordance with the devised method, we have developed the artificial neural network forecasting solution based on the NARX model, in order to forecast both the total electricity consumption from the grid (in the first case) and the total electricity consumption of all the individual appliances (in the second case), using as exogenous variables the second quarter-hourly subset of the timestamps dataset from the first stage. In view of identifying the best combination between the training algorithm (LM, BR or SCG), the number of neurons from the hidden layer (n) and the delay parameter (d), we have tested in each case 36 networks, modifying these settings, by executing 10 iterations per studied case. We have included in the Supplementary Materials file the detailed experimental results that we have obtained after having applied the devised method in both of the cases (Tables S2–S10 and S20–S28).

In the following we have synthetized the obtained results highlighted by the performance metrices (the Mean Squared Error *MSE* and the correlation coefficient *R*), obtained after having developed the NARX artificial neural networks that forecast the total electricity consumption coming from the grid at the level of the entire household for all the eight smart homes in Table 1.

As we have mentioned before, in Section 2, we present an eloquent but non-limitative case of applying the devised method to Smart Home 3, one of the eight studied ones.

**Figure 6:**
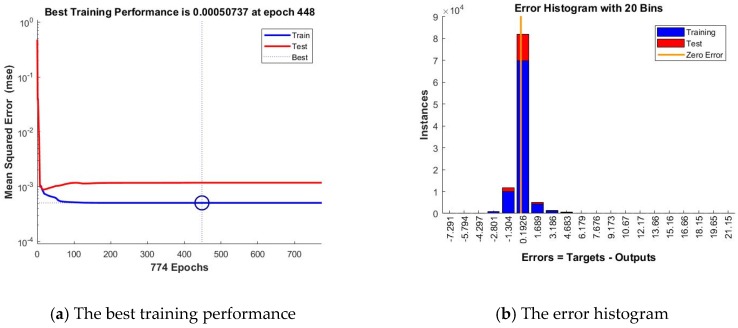

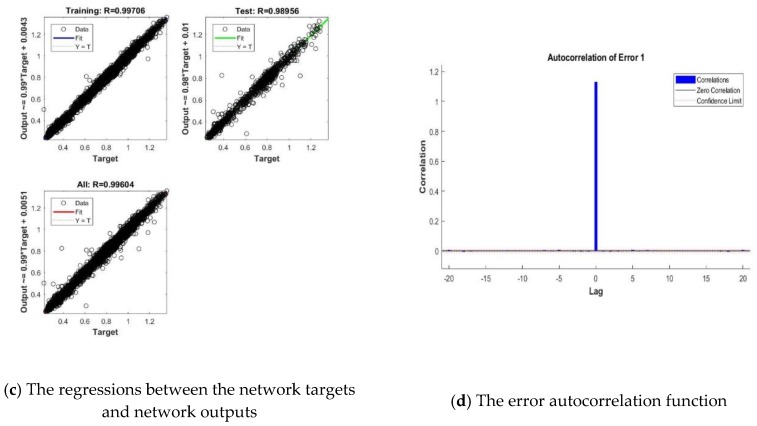
The performance graphs for the artificial neural networks forecasting solution for the total electricity consumption coming from the grid meter, based on the NARX model, in the case of the Smart Home 3.

For this home, we present a synthesis of the experimental results obtained when developing the artificial neural networks forecasting solution based on the NARX model (Table 2) along with the performance graphs that we have obtained for the NARX solution that has provided the best results when forecasting the total electricity consumption coming from the grid at the entire household (Figure 6). Analyzing the performance graphs for the artificial neural networks forecasting solution for the total electricity consumption coming from the grid, one can conclude that the best forecasting accuracy is obtained when using the BR training algorithm, for an artificial neural network with n=24 neurons in the hidden layer and a delay parameter of d=48, case in which has been registered the lowest value of the mean squared error (0.005073) and the closest value to 1 of the correlation coefficient computed for the whole dataset (0.99604).

According to Stage 2, as regarding the ANN NARX forecasting solution for the total electricity consumption of all the individual appliances, using as exogenous variables the second quarter-hourly subset of the timestamps dataset from the first stage, we have used the above-mentioned methodology and synthetized the experimental results (the Mean Squared Error *MSE* and the correlation coefficient *R*) in Table 3.

Examining the obtained results, we have observed that in the case of the Levenberg-Marquardt training algorithm, the best forecasting results are being provided by the ANN whose architecture comprises n=24 neurons in the hidden layer and a delay parameter of d=24, because in this case we have registered the lowest value of the *MSE* (0.0059295) and the closest value to 1 for the correlation coefficient *R* computed for the whole dataset (0.9800476). Contrasting to these results, the ones registered in the case of the forecasting ANN based on the LM training algorithm and the NARX model, using a number of n=12 neurons in the hidden layer and a delay parameter of d=16, have highlighted the worst forecasting accuracy highlighted by the highest value of the Mean Squared Error (0.0066169) and the furthest from 1 value of the correlation coefficient between the network targets and the network outputs for the whole dataset (0.9385478).

Analyzing the results registered in the case of the Bayesian Regularization training algorithm, we have noticed that in this case, the best prediction accuracy is offered by the artificial neural network developed based on an architecture having n=24 neurons in the hidden layer and a delay parameter of d=48. In this case we have obtained the lowest value of *MSE* (0.0051939) and the value of *R* computed for the whole dataset closest to 1 (0.9886501). From the performance forecasting accuracy point of view, the lowest performing network out of the 12 developed ones based on the BR training algorithm, is the one developed using a delay parameter of d=8 and a number of n=6 neurons in the hidden layer, as in this case, we have obtained the highest value of *MSE* (0.0062993) and the lowest value of *R* (0.9586431).

Evaluating the performance metrics registered by the networks developed based on the Scaled Conjugate Gradient (SCG) training algorithm, we have remarked that the network that has been developed using n=6 neurons in the hidden layer and a delay parameter of d=8 has provided the best values for the mean squared error (0.0058914) and the correlation coefficient computed for the whole dataset (0.9811161), while the worst values have been obtained in the case of the network developed using n=12 neurons in the hidden layer and a delay parameter of d=8 (0.0072297 and respectively 0.9301357).

Analyzing the three selected networks, developed based on the LM, BR and SCG training algorithms, we have concluded that the best forecasting accuracy for the total electricity consumption of all the individual appliances is obtained in each case for a different value of the number of neurons in the hidden layer and of the delay parameter. It is worth mentioning that even for the worst networks out of the 36 developed ones, based on the LM, BR or SCG training algorithms, the performance parameters highlight an acceptable forecasting accuracy and the fact that even these networks could have been used in a real-world forecasting scenario. Regarding the training speed, we have noticed that the fastest networks have been the ones developed based on the SCG training algorithm, while the slowest ones have been developed based on the BR training algorithm.

When we have tried to further increase the values of the number of neurons in the hidden layer or of the delay parameter, we have noticed that the training time of the ANNs has increased dramatically (it varies from a few minutes in the case when n=6 and d=8 for the LM training algorithm, up to 20 h in the case when n=24 and d=48 for the BR training algorithm, for a single iteration). In some cases, for a few iterations, neglecting the inconvenience of the extremely long time required for training the networks, while the parameter d has been increasing the forecasting performance has registered a small improvement in terms of accuracy, but the gain was poorly perceptible, practically negligible, and most likely the improvement was not caused by the value of the delay parameter, but by the way in which the dataset has been randomly divided within the 70%, 15% and 15% percentages, as mentioned in the Section 2.2.

**Figure 7:**
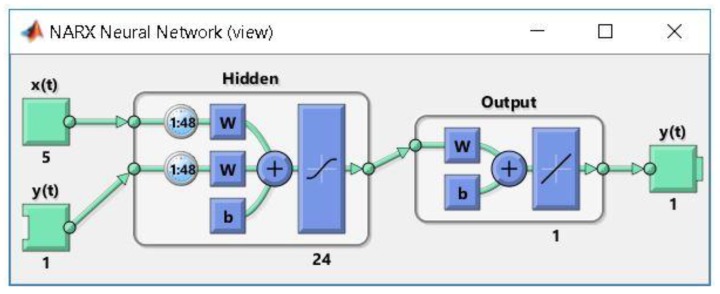
The architecture of the network ANN_NARX_BR.

According to the stages and steps of our devised method, afterwards we have compared the forecasting accuracy of the obtained ANNs, analyzing the performance metrics of the best NARX artificial neural networks, trained based on each of the three algorithms, identifying thus the best NARX ANN forecasting solution for the total electricity consumption of all the individual appliances: the network developed based on the BR training algorithm and on the NARX model, using as exogenous variables the timestamps datasets, with n=24 neurons in the hidden layer and a delay parameter of d=48, entitled ANN_NARX_BR (Figure 7).

In the following, we evaluate this network’s forecasting accuracy, by analyzing the plots representing the performance analysis highlighted by the mean squared error, the error histogram, the regressions, the error autocorrelation. In this purpose, we have first plotted the training and testing curves and, by analyzing the graphic, we have noticed that we have obtained the best training performance at the 1000th epoch, when the mean squared error has the value of 0.0051939.

**Figure 8:**
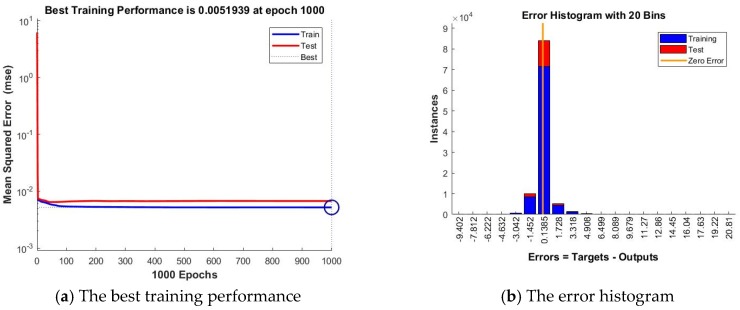

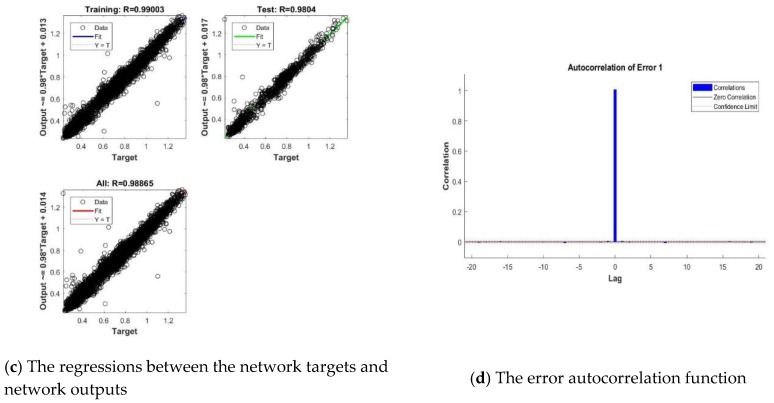
The performance graphs for the artificial neural networks forecasting solution for the total electricity consumption of all the individual appliances, using the ANN_NARX_BR network.

By analyzing the curves represented in this plot, one remarks that they do not increase anymore after their convergence, thus confirming the stability of the devised forecasting solution. Comparing the testing and training curves, we have noticed that the first one does not increase significantly before the second one, highlighting that in this situation the artificial neural network does not overfit the data, due to the fact that the data division has been done in an appropriate manner, while the network’s training has been efficient. Therefore, the chart highlights and confirms the high level of forecasting accuracy and performance of the network ANN_NARX_BR (Figure 8a).

Afterwards, we have represented the error histogram, when forecasting the total electricity consumption of all the individual appliances, using the artificial neural network developed based on the BR training algorithm and on the NARX model, using as exogenous variables the timestamps datasets, with n=24 neurons in the hidden layer and a delay parameter of d=48. The plot highlights the fact that the majority of the errors falls between −1.452 and 1.728, a narrow interval, and only for a very few training points, the errors fall outside this range (most of the errors being around the 0.1385 value). Therefore, the error histogram highlights the fact that Stage 2 of our devised method has output results with a very good forecasting accuracy (Figure 8b).

Subsequently, we have computed and represented the regressions between the network targets and outputs. As all the obtained values of the correlation coefficient R are greater than 0.9804, thus being close to 1, we can conclude that the ANN_NARX_BR network provides a very good fitting between the network targets and outputs (Figure 8c).

Another important plot that we have computed and represented is the one of the error autocorrelation function that reflects the extent to which the forecasting errors are interlinked in time. Analyzing this plot, we have noticed that in this case, excepting the zero-lag correlation, all the other correlations fall within the 95% confidence limits around zero. This result confirms the validity of the forecasting method and the ANN_NARX_BR network’s performance (Figure 8d).

The results obtained for all the eight smart homes are presented in the Supplementary Materials file (Tables S20–S28) and are synthetized below, in Table 4 that contains the comparison of the best experimental results obtained when developing the artificial neural networks forecasting solution for the total electricity consumption of all the individual appliances, based on the NARX model.

Analyzing the best forecasting results from the perspective of the performance metrics, one can observe that the best results have been registered for the BR training algorithm in the cases of the smart homes 1, 2, 3, 5, 6 and 8, for the networks having in most of the cases 24 neurons in the hidden layer and a delay parameter of 48. For the remaining two smart homes, the networks developed based on the LM training algorithm, with 24 neurons in the hidden layer and a delay parameter of 48 have provided the best performance metrics.

As we have stated in Section 2, the differences between the case of Smart Home 3 and the other seven ones are insignificant, the performance parameters having varied slightly, therefore validating the robustness of the proposed method and its potential of generalization.

After having analyzed the performance metrics obtained through the experimental results in the two cases, the first one for predicting of the total electricity consumption from the grid and the second one for predicting the total electricity consumption of all the individual appliances, we have concluded that the forecasting accuracy of the ANN NARX solution is excellent in both cases. The best forecasting accuracy is obtained in both cases when using the BR training algorithm, for artificial neural networks with n=24 neurons in the hidden layer and a delay parameter of d=48, obtaining comparable values of the mean squared error (0.005073 when forecasting the total electricity consumption from the grid, 0.00519 when forecasting the total electricity consumption of all the individual appliances) and of the correlation coefficient computed for the whole dataset (0.996040 in the first case, 0.98865 in the second case). In the following, we present an analysis of the results that we have obtained using the FITNET ANN forecasting solution for the electricity consumption dataset for each of the individual appliances.

### 3.2. Results Regarding the Developed FITNET ANN Forecasting Solution

According to our devised method, in the third stage, we have developed the FITNET ANN forecasting solution for the electricity consumption dataset for each of the individual appliances in two cases, using as input in the first case the total electricity consumption from the grid and in the second case the total electricity consumption of all the individual appliances, along with the timestamps datasets in both cases, while for the output data we have used during the training process the electricity consumption dataset of the individual appliances.

In each case, of particular interest was to pin point the architecture that provided the most accurate forecasting results, therefore we have been researching three training algorithms (LM, BR and SCG) and different values for the configuration parameters by altering the number of neurons from the hidden layer (n) while executing for each size of n a number of 10 iterations in order to determine and save the best FITNET ANN in terms of prediction accuracy. The performance metrics, consisting in the Mean Squared Error (*MSE*) and the correlation coefficient (*R*) computed for the entire dataset by taking into consideration the prediction targets and the network’s outputs, have been recorded and stored for the 135-developed artificial neural networks developed in each case. The comprehensive results for both cases (when forecasting the electricity consumption dataset for each of the individual appliances using in the first case the total electricity consumption from the grid and in the second case the total electricity consumption of all the individual appliances, along with the timestamps datasets in both cases) and for all the eight smart homes (for a number of neurons in the hidden layer varying from 1 to 45) can be found in the Supplementary Materials file (Tables S11–S19 and S29–S37).

In the following we have synthetized the obtained results highlighted by the performance metrices (the Mean Squared Error *MSE* and the correlation coefficient *R*), obtained after having developed the FITNET artificial neural networks that forecast the electricity consumption dataset for each of the individual appliances, using as input the total electricity consumption from the grid and the timestamps datasets at the level of the entire household for all the eight smart homes (Table 5).

As we have mentioned before, in Section 2, we present an eloquent but nonlimitative case of applying the devised method, the one of Smart Home 3 of the eight studied ones. Therefore, a synthesis of the experimental results obtained for Smart Home 3 in the first case, when we have forecasted the electricity consumption dataset for each of the individual appliances, using as input the total electricity consumption from the grid and the timestamps datasets, for a number of neurons (*n*) ranging from 40 to 45, is presented below, in Table 6.

**Figure 9:**
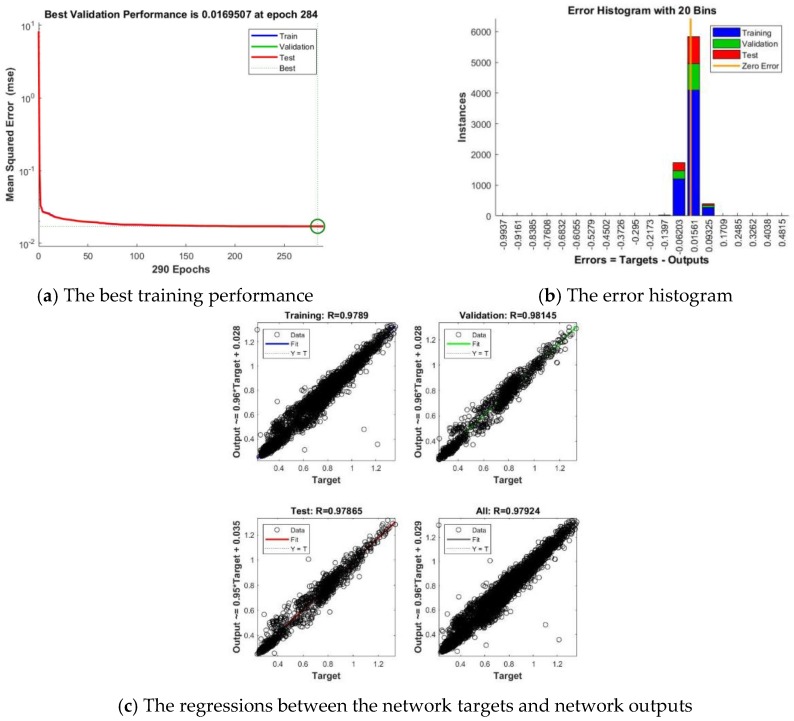
The performance graphs for the artificial neural networks forecasting solution for the electricity consumption dataset for each of the individual appliances, using the total electricity consumption from the grid along with the timestamps dataset.

Analyzing the obtained results, we have remarked that in this case, the best forecasting results (highlighted by the lowest value of *MSE* and the highest value of the correlation coefficient computed for the whole dataset *R*) have been registered in the case when we have trained the FITNET ANN using a number of 44 neurons in the hidden layer and the LM training algorithm. In order to analyze the forecasting accuracy of this network, we have computed and represented the performance plots that depict the *MSE*, the correlation coefficient *R* for the whole dataset as well as the error histogram (Figure 9).

Analyzing the performance plots, we have noticed that the best training performance is achieved at the epoch 284 when the value of the mean square error is 0.0169507 (Figure 9a), the error histogram shows that most of the errors are situated within a narrow range of values, from −0.06203 to 0.9325, most of the errors having a value of around 0.01561 (Figure 9b), while the regressions between the network targets and network outputs highlight the fact that the correlation coefficient *R* for the whole dataset is very close to 1, as *R* = 0.97924 (Figure 9c). As regarding the results obtained in the case when we have developed the FITNET ANNs forecasting solution using the total electricity consumption of all the individual appliances along with the timestamps dataset, we have summarized the obtained experimental results in the case of Smart Home 3, for a number of neurons (*n*) ranging from 40 to 45 in Table 7.

After having examined the experimental results, we have noticed that the artificial neural networks that provide the best forecasting accuracy relative to their corresponding training algorithms have the following architectures and performance metrics: in the situation of the FITNET ANNs trained using the LM algorithm, the best results in terms of forecasting accuracy are offered by the artificial neural network that comprises 44 neurons in the hidden layer, having the lowest value for the performance metric *MSE* (0.016457) while the performance metric *R* corresponding to the entire dataset has the highest value of all the other LM trained networks, being very close to 1 (0.989916); in what concerns the FITNET ANNs developed based on the BR algorithm, the most accurate forecasting is provided by the artificial neural network comprising 43 neurons in the hidden layer, the network being able to record the lowest value for the *MSE* (0.016646) and the highest value closest to 1 for the correlation coefficient *R* (0.989388) computed for the whole dataset; in the case of the FITNET ANNs that were obtained based on the SCG algorithm, the evaluation of the performance metrics reveal that the best forecasting accuracy is obtained when one uses the network that contains 37 neurons in the hidden layer, in this case the *MSE* having the lowest value (0.021458) of all the FITNET ANNs developed based on the SCG algorithm while the correlation coefficient *R* has the closest value to 1 (0.972473).

We have analyzed the three identified FITNET ANNs developed based on the three algorithms (LM, BR, SCG) that have provided the best results in terms of forecasting accuracy and noticed that the most accurate forecasting of the electricity consumption dataset for each of the individual appliances is achieved for each of the cases using a different size of the hidden layer regarding the number of neurons, depending on the algorithm that has been used when developing the network. After having conducted numerous test cases concerning the number of neurons from the hidden layer, we have observed that the most accurate results were achieved for a number of neurons higher than 36 and lower than 45. Even when reaching high numbers of neurons such as 60 in the hidden layer, we still did not record better results without having the problem of overfitting data.

What is worth mentioning is the fact that while the SCG algorithm provided worse results in terms of performance metrics when compared to the LM and BR algorithms, it achieved the results in 1.4% the time BR needed to train the network (for example, 275 min per iteration compared to 4 min per iteration, in the case when *n* = 45) and 2% the time LM needed to provide the trained FITNET ANN (for example, 200 min per iteration compared to 4 min per iteration, in the case when *n* = 45). Depending on the particular case study, whether the networks need frequent retraining when new datasets become available, or simply because the available memory for training the ANNs represents an important constraint, the SCG algorithm might become the algorithm of choice even with worse performance metrics due to increased computational speed and low memory requirements. In the future, we aim to include in the method specific supplementary stages and steps that deal with choosing the best forecasting network by taking into account not only performance metrics but the overall constraints of the contractual involved parties.

**Figure 10:**
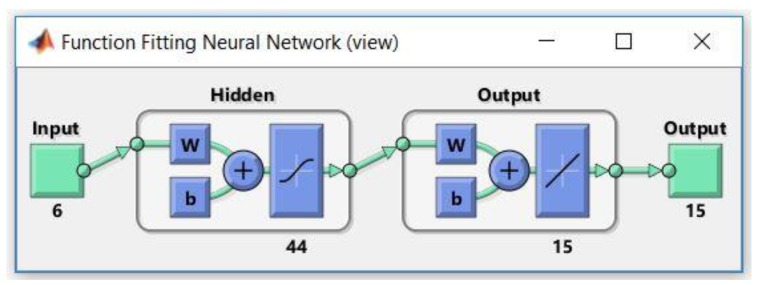
The architecture of the network ANN_FITNET_LM.

Afterwards, according to our developed method, we have compared the prediction accuracy in terms of registered performance metrics of the three identified FITNET ANNs (the best networks, one corresponding to each algorithm) having obtained the best FITNET ANN forecasting solution for the electricity consumption dataset for each of the individual appliances: the fitting neural network developed based on the Levenberg-Marquardt algorithm, having a number of 44 neurons in the hidden layer, taking as inputs the total electricity consumption of all the individual appliances along with the timestamps datasets, entitled ANN_FITNET_LM (Figure 10).

**Figure 11:**
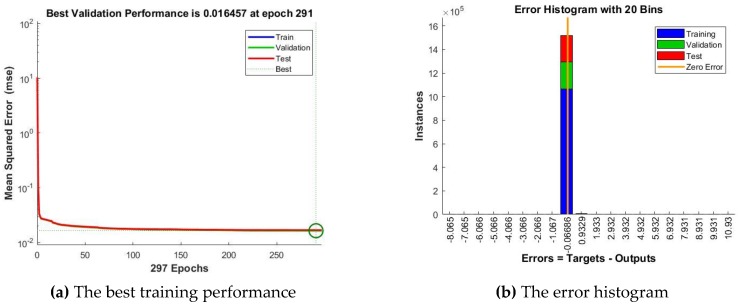

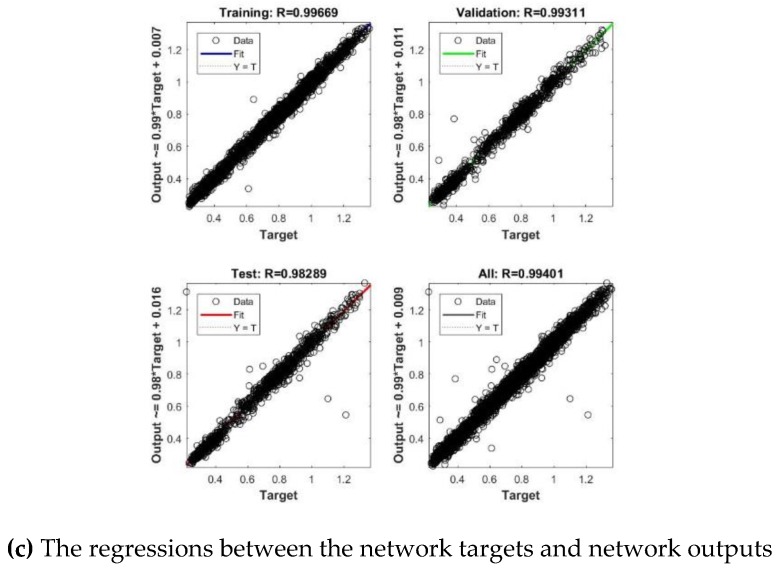
The performance graphs for the artificial neural networks forecasting solution for the electricity consumption dataset for each of the individual appliances, using the ANN_FITNET_LM network.

In the following, we are assessing the ANN_FITNET_LM in terms of prediction accuracy, by performing an analysis of the performance plots that depict the *MSE*, the correlation coefficient *R* for the whole dataset as well as the error histogram. Therefore, we have first plotted the training, validation and testing curves in view of analyzing the best training performance that has been recorded when one forecasts the electricity consumption dataset for each of the individual appliances, using the ANN_FITNET_LM network (Figure 11).

The plot highlights that the best validation performance has been attained at epoch 291 when the mean squared error had recorded a value of 0.016457. We have analyzed the testing curve and we have noticed that it does not increase significantly before the validation one, thus proving the fact that the undesired overfitting process does not take place in this situation.

Consequently, the analysis performed on the plot confirmed that the datasets have been divided accordingly and the training of the FITNET ANN has been performed with utmost efficiency. In addition to this, the analysis of the plot highlights the fact that after having converged, the curves are not increasing any longer, an aspect that once again validates the consistency of the developed FITNET ANN solution in Stage 3 of the devised method, confirming that the network can perform predictions for the electricity consumption dataset for each of the individual appliances with a high degree of accuracy (Figure 11a).

Subsequently, we have computed and plotted the error histogram when the network ANN_FITNET_LM predicts the electricity consumption dataset for each of the individual appliances. Just like the other performance plots did, the error histogram confirms the efficiency and high level of forecasting accuracy of our devised forecasting method as most of the errors are situated within a narrow range of values, from −0.06686 to 0.9329, the preponderance of the errors having a value of around −0.06686 (Figure 11b).

Afterwards, we have plotted the regressions between the network’s targets and its outputs when performing the forecast of the electricity consumption dataset for each of the individual appliances, using the ANN_FITNET_LM network. We have noticed that the value of the correlation coefficient *R* for the whole dataset is very close to 1 (0.99401), therefore confirming that the developed ANN_FITNET_LM network achieves a very good fit and can be successfully used for obtaining quality forecasts (Figure 11c).

The results regarding the forecasting solution for the electricity consumption dataset for each of the individual appliances obtained for the all the eight smart homes are presented in the Supplementary Materials file (Tables S29–S37) and are synthetized in Table 8 below, that contains the comparison of the best obtained experimental results from the perspective of the performance metrics.

The results synthetized in Table 8 show that the best performance metrics have been registered for the BR training algorithm in the cases of the smart homes 1, 4 and 8, for the networks developed based on architectures having a number of 44 or 45 neurons in the hidden layer. For the remaining three smart homes, the networks developed based on the LM training algorithm with 40–45 neurons in the hidden layer have provided the best performance metrics.

As we have mentioned in Section 2, the performance parameters registered for all the eight smart homes are comparable consequently confirming the efficiency, the consistency of the proposed method and its potential of generalization.

Regarding Smart Home 3, the performance metrics obtained through the experimental results in the case when developing the FITNET ANNs forecasting solution using the total electricity consumption from the grid and the FITNET ANNs forecasting solution using the total electricity consumption of all the individual appliances highlight a very good forecasting accuracy in the first case (when stemming from the total electricity consumption from the grid) that improves even further in the second case (when stemming from the total electricity consumption of all the individual appliances). The best forecasting accuracy is obtained in both cases when using the LM training algorithm, for artificial neural networks with n=44 neurons in the hidden layer, obtaining comparable values of the mean squared error (0.016950 when forecasting using the total electricity consumption from the grid, 0.016457 when forecasting using the total electricity consumption of all the individual appliances) and of the correlation coefficient computed for the whole dataset (0.97924101 in the first case, 0.994010 in the second case).

The improvement in the prediction accuracy is determined by the fact that the smart homes self-sustain a part of their consumed electricity by renewable energy generation using photovoltaic panels. As a consequence, the recorded consumption for a certain appliance might have come from the electricity grid, from the solar panel system, its battery storage or even from a mix between the previous sources, therefore influencing the forecasting accuracy of the electricity consumption at the appliance level.

According to the above presented method’s stages and steps, in the following we present the way in which we have obtained the forecast using the best mix of NARX and FITNET ANNs (the networks that have been developed, tested and saved in the Section 3.1 and Section 3.2), thus attaining the final validation of the obtained forecasting solution.

### 3.3. Results Concerning the Validation of the Forecasting Solution

In order to obtain a final validation of our mixt neural network approach in view of forecasting the individual appliances’ electricity consumption of residential consumers based on sensors recorded data, in the next step we have devised a real-world forecasting scenario. Through this scenario, we aim to validate the forecasting solution for the total electricity consumption from the grid (in the first case), for the total electricity consumption of all the individual appliances (in the second case), for each of the individual appliances electricity consumption stemming from for the total electricity consumption from the grid and the timestamps dataset (in the first case), for each of the individual appliances electricity consumption stemming from the total electricity consumption of all the individual appliances and the timestamps dataset (in the second case).

Therefore, in order to validate the forecasting solution for the total electricity consumption from the grid and for the total electricity consumption of all the individual appliances, after forecasting the total electricity consumption from the grid and the total electricity consumption of all the individual appliances dataset using the closed loop form of the best ANNs forecasting solutions based on the NARX model for the next month, we have compared the forecasted results with the total electricity consumption from the grid and the total electricity consumption of all the individual appliances dataset from the second quarter-hourly subset obtained at the end of the first stage.

**Figure 12:**
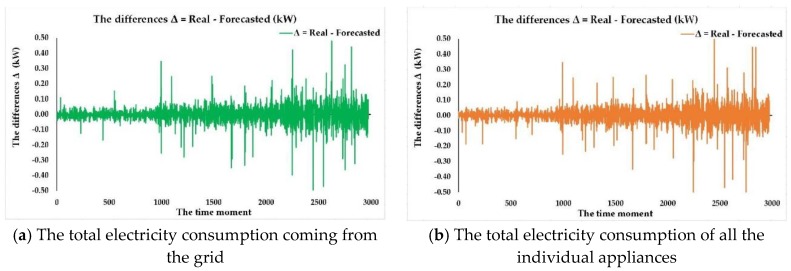
The differences between the real consumption and the forecasted ones when predicting the total electricity consumption, using the best ANNs NARX forecasting solutions.

For obtaining the comparison, we have computed and plotted the values of the differences between the real values of the total electricity consumption and the forecasted ones, for the month of December 2016, consisting in 2976 samples of 1 value. As the differences are very close to the zero value, one can assess that the forecasting results are very close to the real values, confirming thus the accuracy of the developed forecasting solution, based on the NARX model, in both cases: when forecasting the total electricity consumption from the grid (in the first case) and the total electricity consumption of all the individual appliances (in the second case) (Figure 12).

Afterwards, we have forecast the consumption datasets for each of the individual appliances using in each case the best ANN FITNET forecasting solution, therefore obtaining the prediction at the appliance level for the next month. In order to obtain a final validation of the obtained forecasting solution for each of the individual appliances electricity consumption, in the final step of the fourth stage of our devised research method, we have compared the forecasted consumption dataset for each of the individual appliances with the consumption dataset for each of the individual appliances from the second quarter-hourly subset constructed in the first stage, in both cases: stemming from for the total electricity consumption from the grid and the timestamps dataset (in the first case), stemming from the total electricity consumption of all the individual appliances and the timestamps dataset (in the second case).

**Figure 13:**
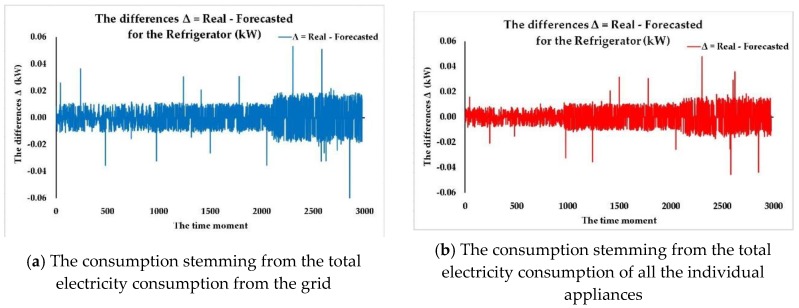
The differences between the real consumption and the forecasted ones when predicting the electricity consumption for a particular appliance (refrigerator), using the best ANNs FITNET forecasting solutions.

In order to obtain a relevant comparison, we have computed and plotted for each individual appliance the values of the differences between the real consumption and the forecasted one for the month of December 2016, consisting in 2976 samples for each of the 15 appliances. The accuracy of the developed forecasting solution, based on the FITNET ANNs, is confirmed by the fact that the forecasting results are close to the real values as the differences are very close to the zero value. In Figure 13 is presented an eloquent but non-limitative example of such a comparison, between the forecast and real electricity consumption of an individual appliance, namely the refrigerator, in both cases: when the appliance’s consumption stems from the total electricity consumption from the grid and when the appliance’s consumption stems from the total electricity consumption of all the individual appliances.

In the following, we analyze the forecasting accuracy for another monitored appliance, one of the rooms (the master bedroom). In this room, the electricity consumption is due to a series of equipment: an air conditioner unit, an electric radiant heating system, lights, a personal computer, a router, a modem, a decoder, an audio system and a TV.

**Figure 14:**
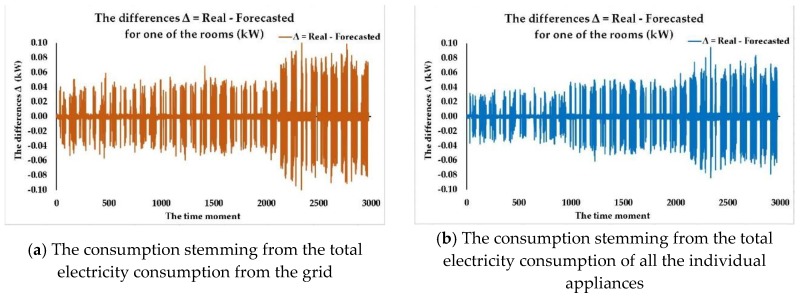
The differences between the real consumption and the forecasted ones when predicting the electricity consumption for one of the rooms, using the best ANNs FITNET forecasting solutions.

In this case, as in the cases of many other home appliances, the consumption has peaks, periods of low consumption and even zero-values, depending on the consumers’ habits and necessities. By analyzing the plot and taking into account the specific electricity consumption of this appliance, one can remark that many values of the differences are very close to the zero value and these zero-differences correspond to those cases where the appliance’s consumption is very low, as these values are forecasted more accurately. The refrigerator operates almost permanently, not being turned off for certain periods of time daily and thus the forecasting method manages to predict its electricity consumption more accurately than in the case of the analyzed room, where the electricity consumption can vary to a large extent due to the consumers’ habits and to the wide range of equipment within the room, being less influenced by the timestamps. The differences between the real consumptions and the forecasted ones when predicting the electricity consumption for one of the rooms, when the appliance’s consumption stems from the total electricity consumption from the grid and when the appliance’s consumption stems from the total electricity consumption of all the individual appliances are highlighted in Figure 14.

**Figure 15:**
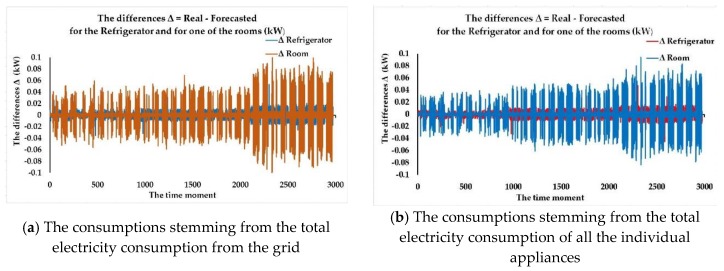
The comparison of the differences between the real consumption and the forecasted ones when predicting the electricity consumption for the refrigerator and for one of the rooms, using the best ANNs FITNET forecasting solutions.

Therefore, except the zero-differences, most of the remaining differences are slightly larger for the master bedroom than in the case of the refrigerator. Therefore, one can conclude that in the case of the bedroom, even if we have obtained a high level of forecasting accuracy, this level is slightly lower than in the case of the refrigerator (Figure 15).

Figure 12 highlights the fact that the differences between the real consumption and the forecasted ones, computed after having used the best ANNs NARX forecasting solutions are comparable in the two cases, when forecasting the total electricity consumption from the grid and the total electricity consumption of all the individual appliances. On the other hand, Figure 13, Figure 14 and Figure 15 highlight the very good accuracy of both approaches and a performance improvement when taking into account the forecasting of the consumption at the appliance level, stemming from the total electricity consumption of all the individual appliances.

We have thus obtained an accurate forecasting of the electricity consumption for each of the individual appliances, along with the validation of our forecasting solution and an important confirmation of the precision, usefulness and reliability of our devised method based on a mix of NARX and FITNET ANNs, in all the analyzed situations: when forecasting the total electricity consumption from the grid, the total electricity consumption of all the individual appliances, the electricity consumption for each of the individual appliances stemming from for the total electricity consumption from the grid, the electricity consumption at the appliances level stemming from the total electricity consumption of all the individual appliances.

## 4. Discussion

We have conducted a study having as a main goal the obtaining of a method that can provide an accurate forecast of the residential electricity consumption at the appliance level, using sensors recorded data, for residential smart homes complexes that sustain a part of their consumed electricity using renewable energy sources, overcoming the limitations of not having historical meteorological data available and unwillingness of the contractor to acquire periodically in the future accurate short-term forecasts from a specialized institute due to the implied costs. As we had access to the individual consumption of different appliances, how to develop a method that was able to forecast the individual electricity consumption for each of the appliances was of particular interest.

In this purpose, we have first developed a series of NARX artificial neural networks, based on the LM, BR and SCG training algorithms, for predicting the total electricity consumption from the grid (in the first case) and the total electricity consumption of all the individual appliances (in the second case), using as input data the total electricity consumption from the grid along with the timestamps dataset (in the first case) and the total electricity consumption of all the individual appliances along with the timestamps dataset (in the second case).

In both cases, we have tested various settings regarding the hidden number of neurons n and the delay parameter d and by comparing the forecasting accuracy of the obtained networks (highlighted by performance metrics) we have selected the best forecasting NARX ANN. Afterwards, we have developed a series of FITNET ANNs based on the LM, BR and SCG training algorithms, for predicting the electricity consumption for each of the individual appliances, using as training input the total electricity consumption from the grid along with the timestamps datasets (in the first case) and the total electricity consumption of all the individual appliances along with the timestamps datasets (in the second case), while we have used as training output the electricity consumption for each of the individual appliances in both cases.

Analyzing the Figure 12, Figure 13, Figure 14 and Figure 15 from the above section, we can notice that all the curves represented in these plots have a common characteristic. Thus, the differences between the real consumptions and the forecasted ones are, in all the analyzed cases, characterized by an oscillatory, generally symmetric trend with respect to the *x*-axis, highlighting a small but non-linear increasing error over the time. Thus, analyzing these plots one can remark that in all the cases the forecasting accuracy is very good for the first 10–12 days of prediction, lowering afterwards for the next 10–12 and again for the remaining days of the forecasted month. The relative symmetry of the charts reflects the fact that our devised method has provided forecasted errors by predicting either greater or lower values than the real ones, alternately, generally to the same extent.

In both of the cases, as it is highlighted in Figure 12 one can notice that the differences between the real consumption and the forecasted ones are comparable when forecasting the total electricity consumption from the grid and when forecasting the total electricity consumption of all the individual appliances. As depicted in Figure 13, Figure 14 and Figure 15, both approaches offer a very good prediction accuracy, while one can notice that the prediction accuracy improves when forecasting the consumption at the appliance level, using the total electricity consumption of all the individual appliances.

In both cases we have conducted a thorough analysis of the results, synthetizing the best forecasting ANNs and their corresponding performance metrics in each of the analyzed cases. In order to analyze the forecasting accuracy of all the 72 NARX ANNs and 270 FITNET ANNs as to obtain a relevant comparison, we have used as a selection criterion the values of the mean squared error (*MSE*) and of the correlation coefficient computed for the whole dataset (*R*), choosing the ANNs for which there has been registered the lowest value of the *MSE* and the value of *R* closest to 1 (Table 9).

Thus, we have noticed that in both cases the best forecasting accuracy of the NARX ANNs developed based on the three different training algorithms is attained for various values of n and d, the best results being the ones provided by the network developed based on the BR training algorithm, for n=24 and d=48. When comparing the performance metrics obtained in the two cases one can observe that the forecasting of the total electricity consumption coming from the grid can be achieved with a slightly higher accuracy, highlighted by both the mean squared error and the correlation coefficient.

Regarding the FITNET ANNs, after testing in both of the cases a wide range of values for the number n of neurons in the hidden layer, we have noticed that the best forecasting results have been provided by the network developed based on the LM training algorithm and a number n=44 of neurons in the hidden layer. Generally, the performance increases along with the values of n and in all the cases the best values of the performance metrics have been obtained for values of 37≤n≤44. In what concerns the performance metrics recorded in the two cases, there can be observed that when forecasting the electricity consumption at the appliance level using the total electricity consumption of all the individual appliances (case 2), one achieves an improved forecasting accuracy compared to the one obtained when using the total electricity consumption coming from the grid (case 1), the improvement being highlighted by the values of the mean squared error and the correlation coefficient.

This improvement regarding the forecasting accuracy can be explained by taking into account the fact that the smart homes have the possibility to sustain a part of their consumed energy using the energy produced from renewable sources, namely PV panels. Therefore, a part of the recorded consumption of a certain appliance is possible to come from the electricity grid, the solar panel system or from its battery storage, all of these factors being able to determine a slightly lower prediction accuracy in the first case (MSE=0.016950, R=0.979241) when compared with the second one (MSE=0.016457, R=0.994010).

In both cases, when we have tried to further increase the values of the number of neurons in the hidden layer or of the delay parameter in the case of the NARX ANNs, or when we have tried to further increase the values of the number of neurons in the hidden layer in the case of the FITNET ANNs, we have noticed that the training time of the ANNs has increased dramatically in the case of the NARX ANNs (it varies from a few minutes in the case when n=6 and d=8 for the LM training algorithm, up to 20 h in the case when n=24 and d=48 for the BR training algorithm, for a single iteration), while in the case of the FITNET ANNs, even if the training time has remained within reasonable limits, either the performance metrics have lowered or the overfitting process has occurred.

In order to highlight the precision and reliability of our devised method based on a mix of NARX and FITNET ANNs, we have synthetized also, in both of the cases, the results regarding the ANNs for which we have registered the highest value of the *MSE* and the value of *R* furthest to 1, values that point out the worst forecasting ANNs (Table 10).

In the first case, the worst prediction accuracy regarding the forecasting of the total electricity consumption coming from the grid using a NARX ANNs solution is recorded in the case of the LM training algorithm, for the neural network having 6 neurons in the hidden layer and the delay parameter of 48. In the second case, the worst prediction forecasting accuracy when predicting the total electricity consumption of all the appliances is recorded by the NARX ANN developed based on the SCG training algorithm, having an architecture that contains 12 hidden neurons and d=8.

In what regards the forecasting of the electricity consumption at the appliance level using the FITNET ANNs, the worst results from the prediction accuracy perspective have been registered in the first case by the network developed based on the SCG training algorithm, having a number of 2 neurons in the hidden layer, and in the second case, by the network developed based on the SCG training algorithm, having one neuron in the hidden layer. By analyzing these results, we have observed that in all the cases, for the FITNET ANNs the worst results have been registered for small values of n (1, 2 or 3). Moreover, even if these networks provide the lowest forecasting accuracy (within a certain model and training algorithm), they still offer a satisfactory forecasting accuracy.

Afterwards, we have analyzed and compared the results presented in Table 9 and Table 10. These results highlight the fact that the *MSE* improvement between the worst and the best forecasting NARX ANNs, ranges in the first case between 12.79% and 18.64%, in the second case between 10.39% and 18.51%, while in the case of the correlation coefficient *R* between the network targets and the network outputs, for the whole dataset, the improvement ranges between 0.87% and 10.77% in the first case and between 3.13% and 5.48% in the second case. In the case of the FITNET ANNs, the improvement of the *MSE* ranges between 20.99% and 36.69% in the first case, between 25.07% and 41.33% in the second case, while in the case of the *R* coefficient, the improvement ranges between 7% and 9.51% in the first case and between 1.27% and 3.52% in the second case (Table 11).

Electricity load forecasting has gained an ever-increasing interest during the decades due to the multiple implications that it poses to both the supplier and the consumer. Being able to provide an accurate forecast of the electricity consumption creates the premises of achieving energy efficiency by minimizing the waste of energy and attaining a balance between the demand and the offer. Most of the case studies from the literature aim to forecast the aggregated consumption of a certain region as this type of forecast is more attainable than a residential electricity consumption forecast, being especially easier to obtain than a detailed electricity consumption of certain appliances in different households. Nowadays, the fact that smart-meters are becoming implemented on a wider scale, makes it possible to have access to more detailed data and devise more refined methods.

When analyzing the literature, one can observe numerous resemblances between the methods used to forecast residential electricity consumptions and the ones applied for aggregated consumptions. One can distinguish between two broad approaches. The first one consists in statistical modelling methods while the second one employs machine learning techniques benefiting most from artificial neural networks and deep-learning implementations. Conventional linear methods such as autoregressive (AR), vector autoregressive (VAR), moving average (MA), autoregressive–moving-average (ARMA), autoregressive integrated moving average (ARIMA) are used nowadays mostly as baselines taking into account that they have been surpassed in terms of prediction accuracy by most of the existing machine learning related methods [37].

In our previous studies [30], we managed to successfully devise a method that was able to predict with high accuracy the total electricity consumption in the case of commercial center type consumers (hypermarkets from a large hypermarket chain in Bucharest, Romania). First, we tried to apply solutions that provided satisfactory results in the case of our previous researches [30,38,39] but also in other researches from the scientific literature [26,27]. The main challenge that we faced this time was that the contractor needed a validated method incorporated in a cloud solution, along with the method that we have developed in [30] for commercial center type consumers, but this time the forecast of the total electricity consumption was of secondary interest, the contractor needed a method that was able to forecast reliably the individual consumption of certain appliances for at least 7 days ahead, taking into consideration the particular aspect that the devised method was about to be applied in a residential complex where all the smart homes were also equipped with photovoltaic systems for self-generating a part of their consumed energy.

Therefore, the method devised in [30] targets commercial center type consumers while the method devised within this paper targets residential consumers. While in the method devised in [30] the forecasting of the total electricity consumption is of primary interest, in this paper’s case the individual consumption of certain appliances within the household is of primary interest. The hypermarkets on which the method from [30] was validated, did not have any electricity self-generating equipment installed to sustain a part of the consumption. In the case of the method presented in this paper, all the eight households have installed photovoltaic systems that sustain a part of the necessary electricity.

Regarding the developed forecasting solutions, the method devised for commercial center type consumers provided good results when developing artificial neural networks (ANNs) forecasting solutions based on the nonlinear autoregressive (NAR) model, ANNs based on the non-linear autoregressive with exogenous inputs (NARX) model using as exogenous variables a meteorological and timestamps dataset and ANNs based on the NARX model that make use for the exogenous variables of a timestamps dataset.

In the case of forecasting the individual appliances consumption of the residential consumers, ANNs based on the NAR model provided poor results in terms of accuracy, while in the case of the NARX model, we were not able to forecast directly the consumption of the individual appliances, having recorded performance metrics that reflected a low forecasting accuracy. However, the ANNs NARX model proved to be very useful in the forecasting of the total electricity consumption coming from the grid and of the total electricity consumption of all the appliances, but in this situation, in contrast with the commercial center type consumer, the smart homes had the ability to self-sustain a part of their electricity consumption.

In the case of the method devised in [30], the data was recorded every hour for the whole year 2016 and a meteorological dataset was available, while in the case of the residential consumer the data was recorded every 15 min for the years 2014–2016, without having access to meteorological data. Therefore, the timestamp dataset had to be constructed differently in the case of the residential consumer, containing the hour of the day, the minute of the hour, the day of the week, the day of the month and the month of the year and we had to devise the method without using meteorological data. Even if we had had available a weather dataset to use during the training process, it would have been of little help when trying to forecast future electricity consumptions using the trained network as we would have needed forecasted weather data with a high degree of accuracy from a specialized institute every 15 min for the respective month at a refined resolution.

Our devised method uses in the last steps of the forecast FITNET ANNs that we developed in order to forecast accurately the individual consumption of the appliances using the forecasted total electricity consumption coming from the grid along with the timestamps dataset in the first case and the forecasted total electricity consumption of all the appliances along with the timestamps dataset. Both the above mentioned total consumptions have been forecasted by the developed and validated NARX solution. When devising stage 3 of our method, in both of the cases, as we wanted to see if we can further improve the prediction accuracy, we have also tried to train the FITNET ANNs using predicted data for the month of December of the NARX ANNs. Therefore, we were faced with several drawbacks: the forecasted data did not have enough samples for the FITNET ANNs to be able to train appropriately; the timestamps were limited only to a single month (as the prediction accuracy of the NARX ANNs is very high initially for the first 10–12 days but afterwards it starts to decrease and is no longer reliable when forecasting more than a month) so the networks could not have been trained for more than a month; the performance metrics reflected a less accurate forecast than when training the FITNET ANNs using the total electricity consumption from the grid (case 1) and the one of all the appliances measured by the sensors (case 2) and forecasting the consumption of each individual appliance.

In our paper, we have devised a mixed neural network approach in order to forecast the residential electricity consumption based on sensors recorded data, using both NARX and FITNET ANNs, obtaining accurate forecasting results that reveal the precision and reliability of our devised method. Many other studies have targeted one or other of these two approaches, for example in [29] the authors have used an implementation of the NARX model in order to forecast the electric loads, using as exogenous variables timestamps and meteorological data. The authors obtain a 24-h ahead load forecasting, resulting in an hourly dataset. Their method provides accurate results but, in order to implement it, one requires an accurate weather forecasting for the next day for obtaining the exogenous data. In our approach, as we targeted to predict the electricity consumption for at least 7 days, every 15 min, we couldn’t rely on obtaining an accurate weather forecast at such a refined resolution. Thus, we have managed to avoid this inconvenience, by using only the timestamp dataset. Meanwhile, our forecasting method targets a wider prediction timeframe, even if its accuracy lowers over time. The method devised in [29] targets the electrical load in a grid, while our method targets the residential consumption, refining it at the appliances level of a household.

As in our paper, the authors of [23] devise in that paper a forecasting method without using meteorological data based on ANNs, but in contrast to our method they propose a 24-h-ahead load forecasting, also based on: wavelet transform and radial basis function neural networks; wavelet transform and artificial neural networks; empirical mode decomposition and radial basis function neural networks. The method developed in [23] aims to forecast the aggregated consumption at the level of the entire country (Turkey), in contrast to our devised method that targets the residential consumption, breaking it down at individual appliances.

As in the previously mentioned paper, Tsekouras et al. [24] analyzes an approach for developing a short-term forecasting solution. The authors target the Greek power system load demand forecasting, and in this purpose, they have developed several feed-forward artificial neural networks while in our paper we have devised a mixed ANNs approach that uses sensors recorded data to predict the residential electricity consumption at the appliances level. Just like in our paper, in [24] the authors remark that the best results have been obtained when using a training set of 3 years. Regarding the time horizon of the prediction, the authors aim to obtain an hourly prediction starting from a few hours up to 1 week while our method aims to obtain a quarter-hourly prediction having a time horizon starting from 1 week up to a whole month.

In our mixed approach using the NARX and FITNET ANNs, we have tested three training algorithms (Levenberg-Marquardt, Bayesian Regularization, Scaled Conjugate Gradient) in order to develop the artificial neural networks, compared the obtained networks, choosing afterwards the algorithm that has offered the best performance metrics, while in paper [26], the authors have used only the LM training algorithm in order to develop ANNs based only on NAR and NARX models. The authors targeted in their forecast a set of public buildings over a period of 100 days, using a single prediction data point per day for the aggregated consumption of the respective building while in our devised method we have targeted the prediction of the electricity consumption of residential consumers and of certain monitored appliances from the respective households, over a period of up to one month, using 96 data points per day for each of the appliances.

Regarding the developed ANNs, both our approach and the one in [26], are based on testing different settings, by adjusting the networks’ parameters, in order to identify the setting that offers the best forecasting results. The main difference between the two testing procedures is the fact that in the paper [26], the authors first test various settings regarding the delay parameter d, choose and set its value, and afterwards they test various settings regarding the number of neurons in the hidden layer. Thus, they use a serial approach, obtaining the values of the two parameters one after the other. In contrast to this method, in our approach, for both of the cases, we have tested various (n,d) pairs, hence developing a parallel approach for obtaining the two parameters simultaneously. Correlating this parallel approach with the testing of the three different training algorithms, we have taken into consideration a wide variety of cases. Both the method described in [26], and our devised method based on a mixed neural network approach provide very good results in terms of forecasting accuracy for the purposes for which they have been developed.

Based on an artificial neural network approach, in [25] the authors develop a day ahead load forecasting method in the case of the power system in Turkey, taking into account the load variation caused by national or religious holidays, in contrast to our research in which we have considered the timestamps datasets as exogenous variables that influence the residential electricity consumption. In order to evaluate the obtained performance, we have used as performance metrics the mean squared error, the error histogram, the regressions, the error autocorrelation, while in [25] the authors take into account the mean absolute percentage error and the hourly absolute percentage error.

In the paper [27], the authors propose a method based on Deep Neural Networks using two versions of the Long Short-Term Memory (LSTM) algorithms: a standard version and a version that employs a Sequence to Sequence architecture. The method is tested only on a single residential consumer, using data at a one-minute resolution and one-hour resolution that has been computed using the average of the one-minute dataset. The authors state that the standard LSTM model failed to predict more steps ahead in the future, being able to be used only for one-step ahead accurate predictions. The authors tried to improve the method by providing multiple steps back as inputs to the network, obtaining a method that was suitable for forecasting data accurately every hour for a number of hours corresponding to how many previous real recorded values were used from the past as inputs. In this purpose, the authors gave an example, which highlights that using 60 real values of previously recorded hourly data as inputs makes the LSTM Neural Network able to provide exactly 60 h ahead of forecasting with good accuracy only to fail afterwards. In the case of the dataset having a resolution of 1 min, the network failed to provide the desired output for more than one step ahead, no matter how many previous steps had been used. The second version of the network that made use of a Sequence to Sequence architecture offered good prediction accuracy in both the situations and the authors concluded that they intend to use their developed solution on real world datasets in order to assess in a reliable way the efficiency of their proposed solution.

When compared to the method from [27], one can notice several differences between the implementations: when developing our method, we have used different real-world datasets with a resolution of 15 min concerning residential households and some of their monitored appliances. Our method relies on different types of neural networks being used in different stages of the method, ANNs based on the NARX model and FITNET ANNs in order to forecast the consumption of certain appliances within the households with a very good prediction accuracy for a time horizon of 10–12 days and good prediction accuracy for up to 31 days.

In recent years, forecasting the electricity consumption accurately has been at the forefront of numerous researches, there have been developed and employed thousands of different forecasting methods targeting numerous case studies, each method trying to improve on certain aspects and ultimately provide a more accurate, reliable forecast in the context of the respective case studies. Notwithstanding that researchers are developing and applying even as we are writing this article, for numerous patents based on electricity forecasting methods and equipment, it is very hard to make a 100% relevant comparison between the wide variety of forecasting methods from the scientific literature, as the discussed papers refer to different datasets and case studies.

A very important factor that should be taken into consideration when comparing the forecasting methods is their effectiveness, that highlights their capacity to be applied to many other cases and situations, by generalization. From this point of view, our developed method has the ability to be tested and adapted to other datasets recorded by sensors from residential consumers and their appliances. This ability has been proven by the fact that we have implemented the same method to 8 smart homes and we have obtained similar results, as the variations of the performance parameters have been insignificant.

In the analyzed situation, we considered that the consumers had the same energy class, the same brand of appliances. In the future, we aim to refine the method as to take into consideration the energy class of the appliances, the number of inhabitants, their age and certain behavioral characteristics. To this purpose we aim to acquire a future version of the development environment that offers extended support for developing long short-term memory (LSTM) networks, in order to employ deep learning for labeling the households according to certain characteristics therefore refining the forecasting method even more. Another aspect that we intend to improve in our future work consists in optimizing the retraining process, taking into account that at this moment the forecasting networks must be retrained at the end of each forecasted month, using the real values recorded by the sensors, in order to at least preserve and even improve the forecasting accuracy.

As in the case of many other scientific papers, our developed forecasting method does not guarantee an optimal solution, but just the fact that we have chosen the best available solution of those corresponding to this methodology, by choosing the most appropriate combination of the various parameters involved. However, by validating the method, as well as by comparing the obtained results with others from the literature, we can conclude that our developed method represents a viable, accurate alternative to those already existing in the literature and it can be compiled, incorporated into the cloud solution, delivered to the contractor and used in a production environment.

## 5. Conclusions

Our devised method offers very good prediction results for a time horizon of 10–12 days and good results for up to 31 days, being a useful tool for the contractor, having the prospects of being incorporated in a cloud solution that is useful to both the consumers and the operators.

The compiled method incorporated in the cloud solution is useful to the consumers in terms of optimizing their electricity consumption, choosing the best billing plan or receiving notifications in real time that can help them in the decisional process or even altering their consumption behavior in a beneficial way.

In what concerns the operators, they can provide to the consumers the necessary equipment to monitor the appliances and credentials to access the cloud solution for a certain monthly fee, while being able to offer more and more flexible services to their customers and store with the consent of their customers, anonymized data that can provide valuable insight when tailoring their market offers to the customers behavioral needs.

## Figures and Tables

**Table 1:** The comparison of the best experimental results recorded for Smart Homes 1–8 when developing the artificial neural networks forecasting solution for the total electricity consumption coming from the grid meter, based on the NARX model.

The Best Forecasting Results								
**Smart Home number**	1	2	3	4	5	6	7	8
**Training algorithm**	BR	BR	BR	BR	BR	BR	BR	BR
**Number of neurons in the hidden layer *n***	12	24	24	24	24	24	24	24
**Delay parameter *d***	48	48	48	48	48	48	48	48
***MSE***	0.004934	0.005245	0.005073	0.004986	0.005401	0.004986	0.005401	0.005141
***R***	0.993635	0.972497	0.996040	0.999661	0.961846	0.998536	0.968877	0.999894

**Table 2:** The experimental results for the Smart Home 3, when developing the artificial neural networks forecasting solution for the total electricity consumption coming from the grid meter, based on the NARX model.

**The Levenberg-Marquardt Training Algorithm**					
*n*	*d*	8	16	24	48
6	*MSE*	0.00605157	0.00632257	0.00621917	0.006476481
	*R*	0.98266076	0.93192179	0.92554297	0.901322016
12	*MSE*	0.00599985	0.00641839	0.00635471	0.005923155
	*R*	0.99099007	0.98547519	0.99048373	0.997892168
24	*MSE*	0.00613578	0.00575786	0.0057937	0.005633025
	*R*	0.98754	0.99164337	0.99192495	0.998358915
**The Bayesian Regularization Training Algorithm**					
*n*	*d*	8	16	24	48
6	*MSE*	0.00623631	0.00600871	0.00601187	0.005692526
	*R*	0.98740239	0.99599301	0.99137728	0.990196752
12	*MSE*	0.00595851	0.00593909	0.00566544	0.005480209
	*R*	0.99111431	0.99073475	0.99195002	0.993635819
24	*MSE*	0.00583027	0.00596158	0.0054246	0.0050737
	*R*	0.99032837	0.99099293	0.99643307	0.99604010
**The Scaled Conjugate Gradient Training Algorithm**					
*n*	*d*	8	16	24	48
6	*MSE*	0.00617735	0.00617295	0.0061248	0.006461376
	*R*	0.99007384	0.96872312	0.98194432	0.984417105
12	*MSE*	0.00686822	0.00640441	0.00668772	0.006339564
	*R*	0.95803977	0.97438288	0.95322142	0.96202096
24	*MSE*	0.00638039	0.00672022	0.0068212	0.005832486
	*R*	0.97581848	0.95323304	0.9678163	0.99399508

**Table 3:** The experimental results for the Smart Home 3, when developing the artificial neural networks forecasting solution for the total electricity consumption of all the individual appliances, based on the NARX model.

**The Levenberg-Marquardt Training Algorithm**						
*n*	*d*	8	16	24	48	Trend
6	*MSE*	0.0061127	0.0064516	0.0064783	0.0065419	↑↑↑
	*R*	0.9633929	0.9509406	0.9444316	0.9388771	↓↓↓
12	*MSE*	0.0061223	0.0066169	0.0064189	0.0062349	↑↓↓
	*R*	0.9618102	0.9385478	0.9523882	0.9595117	↓↑↑
24	*MSE*	0.006261	0.0060609	0.0059295	0.0060351	↓↓↑
	*R*	0.9587767	0.9680321	0.9800476	0.9692805	↑↑↓
**The Bayesian Regularization Training Algorithm**						
*n*	*d*	8	16	24	48	Trend
6	*MSE*	0.0062993	0.0060694	0.0060726	0.0058087	↓↑↓
	*R*	0.9586431	0.9669835	0.9654979	0.9823211	↑↓↑
12	*MSE*	0.0060187	0.0060603	0.0059015	0.0056497	↑↓↓
	*R*	0.9716807	0.9686034	0.9802887	0.9870078	↓↑↑
24	*MSE*	0.0060732	0.0060218	0.0057101	0.0051939	↓↓↓
	*R*	0.9646959	0.9715617	0.9865674	0.9886501	↑↑↑
**The Scaled Conjugate Gradient Training Algorithm**						
*n*	*d*	8	16	24	48	Trend
6	*MSE*	0.0058914	0.0062353	0.00638	0.0067306	↑↑↑
	*R*	0.9811161	0.9591318	0.953344	0.9375401	↓↓↓
12	*MSE*	0.0072297	0.0064691	0.0068242	0.0064036	↓↑↓
	*R*	0.9301357	0.9460028	0.9345308	0.952496	↑↓↑
24	*MSE*	0.0067162	0.0067881	0.0068901	0.0063684	↑↑↓
	*R*	0.938287	0.9345422	0.9305926	0.9557645	↓↓↑

**Table 4:** The comparison of the best experimental results recorded for Smart Homes 1–8 when developing the artificial neural networks forecasting solution for the total electricity consumption of all the individual appliances, based on the NARX model.

The Best Forecasting Results								
**Smart Home number**	1	2	3	4	5	6	7	8
**Training algorithm**	BR	BR	BR	LM	BR	BR	LM	BR
**Number of neurons in the hidden layer *n***	24	6	24	24	24	24	24	24
**Delay parameter *d***	48	48	48	48	48	24	48	48
***MSE***	0.004986	0.005566	0.00519	0.005038	0.005297	0.005053	0.005401	0.005090
***R***	0.998536	0.972497	0.98865	0.996988	0.968877	0.999358	0.978763	0.999183

**Table 5:** The comparison of the best experimental results recorded for Smart Homes 1–8 when developing the FITNET ANN forecasting solution using the total electricity consumption from the grid along with the timestamps dataset.

The Best Forecasting Results								
**The Smart Home number**	1	2	3	4	5	6	7	8
**The training algorithm**	BR	BR	LM	LM	BR	BR	BR	LM
**The number of neurons in the hidden layer *n***	43	45	44	44	45	44	43	45
***MSE***	0.0168124	0.0169730	0.016950	0.01662157	0.0168801	0.0169599	0.0168124	0.0166215
***R***	0.9794941	0.9791961	0.974322	0.97957431	0.9795743	0.9789337	0.9795743	0.9791634

**Table 6:** The experimental results for Smart Home 3, when developing the FITNET ANNs forecasting solution using the total electricity consumption from the grid along with the timestamps dataset.

*n*	The Training Algorithm	The Levenberg-Marquardt Training Algorithm	The Bayesian Regularization Training Algorithm	The Scaled Conjugate Gradient Training Algorithm
40	*MSE*	0.0181737	0.01779952	0.02454112
	*R*	0.9360958	0.92975682	0.9185132
41	*MSE*	0.01705486	0.0171498	0.0225784
	*R*	0.95790604	0.96671806	0.94268383
42	*MSE*	0.01763424	0.0178133	0.02409264
	*R*	0.91767006	0.94926912	0.93778921
43	*MSE*	0.01783238	0.01714538	0.0222309
	*R*	0.96833996	0.94981248	0.96087024
44	*MSE*	0.01695071	0.01784974	0.02405416
	*R*	0.97924101	0.949464	0.95729733
45	*MSE*	0.01793427	0.01771578	0.02328641
	*R*	0.92971076	0.95941439	0.94260623

**Table 7:** The experimental results for Smart Home 3, when developing the FITNET ANNs forecasting solution using the total electricity consumption of all the individual appliances along with the timestamps dataset.

*n*	The Training Algorithm	The evenberg-Marquardt Training Algorithm	The Bayesian Regularization Training Algorithm	The Scaled Conjugate Gradient Training Algorithm
40	*MSE*	0.017145	0.016792	0.023152
	*R*	0.985364	0.989025	0.966856
41	*MSE*	0.016886	0.016980	0.021710
	*R*	0.987532	0.986447	0.971839
42	*MSE*	0.016956	0.016805	0.023166
	*R*	0.986742	0.988822	0.966793
43	*MSE*	0.016823	0.016646	0.023083
	*R*	0.988102	0.989388	0.967575
44	*MSE*	0.016457	0.016682	0.023129
	*R*	0.994010	0.989103	0.966967
45	*MSE*	0.016761	0.016713	0.021763
	*R*	0.989054	0.989087	0.971759

**Table 8:** The comparison of the best experimental results recorded for Smart Homes 1–8 when developing the FITNET ANNs forecasting solution using the total electricity consumption of all the individual appliances.

The Best Forecasting Results								
**Smart Home number**	1	2	3	4	5	6	7	8
**Training algorithm**	BR	LM	LM	BR	LM	LM	LM	BR
**Number of neurons in the hidden layer *n***	45	40	44	45	44	45	45	44
***MSE***	0.015477	0.016809	0.016457	0.015877	0.016621	0.015922	0.016621	0.016181
***R***	0.993854	0.975510	0.994010	0.995865	0.977166	0.995861	0.970163	0.998994

**Table 9:** The best forecasting ANNs and their corresponding performance metrics.

The Training Algorithm	Case 1: Forecasting the Total Electricity Consumption from the Grid and, Based on It, the Electricity Consumption at the Appliance Level		Case 2: Forecasting the Total Electricity Consumption of All the Appliances and, Based on It, the Electricity Consumption at the Appliance Level	
	NARX ANNs	FITNET ANNs	NARX ANNs	FITNET ANNs
LM	n=24, d=48	n=44	n=24, d=24	n=44
	MSE=0.005633	MSE=0.016950	MSE=0.0059295	MSE=0.016457
	R=0.998358	R=0.979241	R=0.9800476	R=0.994010
BR	n=24, d=48	n=41	n=24, d=48	n=43
	MSE=0.0050737	MSE=0.017149	MSE=0.0051939	MSE=0.016646
	R=0.996040	R=0.966718	R=0.9886501	R=0.989388
SCG	n=6, d=48	n=43	n=6, d=8	n=37
	MSE=0.005832	MSE=0.022309	MSE=0.0058914	MSE=0.021458
	R=0.993995	R=0.960870	R=0.9811161	R=0.972473

**Table 10:** The worst forecasting ANNs and their corresponding performance metrics.

The Training Algorithm	Case 1: Forecasting the Total Electricity Consumption from the Grid and, Based on It, the Electricity Consumption at the Appliance Level		Case 2: Forecasting the Total Electricity Consumption of All the Appliances and, Based on It, the Electricity Consumption at the Appliance Level	
	NARX ANNs	FITNET ANNs	NARX ANNs	FITNET ANNs
LM	n=6, d=48	n=3	n=12, d=16	n=1
	MSE=0.006476	MSE=0.026117	MSE=0.0066169	MSE=0.028051
	R=0.901322	R=0.894174	R=0.9385478	R=0.960242
BR	n=6, d=8	n=2	n=6, d=8	n=1
	MSE=0.006236	MSE=0.027088	MSE=0.0062993	MSE=0.027318
	R=0.987402	R=0.903465	R=0.9586431	R=0.961006
SCG	n=12, d=24	n=2	n=12, d=8	n=1
	MSE=0.006687	MSE=0.028236	MSE=0.0072297	MSE=0.028638
	R=0.953221	R=0.893135	R=0.9301357	R=0.960234

**Table 11:** The performance metrics improvement when comparing the best with the worst forecasting ANNs.

The Training Algorithm	Case 1: Forecasting the Total Electricity Consumption from the Grid and, Based on It, the Electricity Consumption at the Appliance Level		Case 2: Forecasting the Total Electricity Consumption of All the Appliances and, Based on It, the Electricity Consumption at the Appliance Level	
	The Best NARX ANNs Versus the Worst One	The Best FITNET ANNs Versus the Worst One	The Best NARX ANNs Versus the Worst One	The Best FITNET ANNs Versus the Worst One
LM	13.02% for the *MSE*	35.1% for the *MSE*	10.39% for the *MSE*	41.33% for the *MSE*
	10.77% for *R*	9.51% for *R*	4.42% for *R*	3.52% for *R*
BR	18.64% for the *MSE*	36.69% for the *MSE*	17.55% for the *MSE*	39.07% for the *MSE*
	0.87% for *R*	7.00% for *R*	3.13% for *R*	2.95% for *R*
SCG	12.79% for the *MSE*	20.99% for the *MSE*	18.51% for the *MSE*	25.07% for the *MSE*
	4.28% for *R*	7.58% for *R*	5.48% for *R*	1.27% for *R*

## Supplementary Materials

The following are available online at http://www.mdpi.com/1424-8220/18/5/1443/s1. Table S1: An overview of the method’s stages and steps; Tables S2–S9: The experimental results for the Smart Homes 1–8, when developing the artificial neural networks forecasting solution for the total electricity consumption coming from the grid meter, based on the NARX model; Table S10: The comparison of the best experimental results recorded for Smart Homes 1–8 when developing the artificial neural networks forecasting solution for the total electricity consumption coming from the grid meter, based on the NARX model; Tables S11–S18: The experimental results for the Smart Homes 1–8, when developing the FITNET ANNs forecasting solution using the total electricity consumption from the grid; Table S19: The comparison of the best experimental results recorded for Smart Homes 1–8 when developing the FITNET ANNs forecasting solution using the total electricity consumption from the grid; Tables S20–S27: The experimental results for the Smart Homes 1–8, when developing the artificial neural networks forecasting solution for the total electricity consumption of all the individual appliances, based on the NARX model; Table S28: The comparison of the best experimental results recorded for Smart Homes 1–8 when developing the artificial neural networks forecasting solution for the total electricity consumption of all the individual appliances, based on the NARX model; Tables S29–S36: The experimental results for the Smart Homes 1–8, when developing the FITNET ANNs forecasting solution using the total electricity consumption of all the individual appliances; Table S37: The comparison of the best experimental results recorded for Smart Homes 1–8 when developing the FITNET ANNs forecasting solution using the total electricity consumption of all the individual appliances.
