# Supplementary material for: Developing a Mixed Neural Network Approach to Forecast the Residential Electricity Consumption Based on Sensor Recorded Data
Source: Sensors (Basel). 2018 May 5;18(5):1443. doi: 10.3390/s18051443 (PMC5981650; doi:10.3390/s18051443)
Supplement: Supplementary file 1 [file sensors-18-01443-s001.pdf]

sensors

Supplementary Materials

Developing a Mixt Neural Network Approach to Forecast the Residential Electricity Consumption Based on Sensors Recorded Data

Simona-Vasilica Oprea[1], Alexandru Pîrjan[2,*], George Căruțașu[2], Dana-Mihaela Petroșanu[2,3], Adela Bâra[1], Justina-Lavinia Stănică[2], Cristina Coculescu[2]

[1] Department of Economic Informatics and Cybernetics, The Bucharest Academy of Economic Studies, Romana Square 6, Bucharest 010374, Romania; simona.oprea@csie.ase.ro (S.-V.O.); bara.adela@ie.ase.ro (A.B.)
[2] Department of Informatics, Statistics and Mathematics, Romanian-American University, Expoziției 1B, Bucharest 012101, Romania; carutasu.george@profesor.rau.ro (G.C.); danap@mathem.pub.ro (D.-M.P.); stanica.lavinia.justina@profesor.rau.ro (J.-L.S); coculescu.cristina@profesor.rau.ro (C.C.)
[3] Department of Mathematics-Informatics, University Politehnica of Bucharest, Splaiul Independenței 313, Bucharest 060042, Romania
* Correspondence: alex@pirjan.com; Tel.: +40-762-642-866

The Supplementary Materials file contains:

- **Table S1**: An overview of the method's stages and steps;
- **Tables S2-S9**: The experimental results for the Smart Homes 1-8, when developing the artificial neural networks forecasting solution for the total electricity consumption coming from the grid meter, based on the NARX model.
- **Table S10**: The comparison of the best experimental results recorded for Smart Homes 1-8 when developing the artificial neural networks forecasting solution for the total electricity consumption coming from the grid meter, based on the NARX model.
- **Tables S11-S18**: The experimental results for the Smart Homes 1-8, when developing the FITNET ANNs forecasting solution using the total electricity consumption from the grid. **Table S19**: The comparison of the best experimental results recorded for Smart Homes 1-8 when developing the FITNET ANNs forecasting solution using the total electricity consumption from the grid.
- **Tables S20-S27**: The experimental results for the Smart Homes 1-8, when developing the artificial neural networks forecasting solution for the total electricity consumption of all the individual appliances, based on the NARX model.
- **Tables S28**: The comparison of the best experimental results recorded for Smart Homes 1-8 when developing the artificial neural networks forecasting solution for the total electricity consumption of all the individual appliances, based on the NARX model.
- **Tables S29-S36**: The experimental results for the Smart Homes 1-8, when developing the FITNET ANNs forecasting solution using the total electricity consumption of all the individual appliances.
- **Table S37**: The comparison of the best experimental results recorded for Smart Homes 1-8 when developing the FITNET ANNs forecasting solution using the total electricity consumption of all the individual appliances.

The stages and steps of our devised method are synthetized in Table S1.

S1. An overview of the method's stages and steps.

Stage	Step	Input	Output	Final Results of the Stage
I. Acquiring and processing the data collected from the sensors	1. Acquiring the electricity consumption datasets recorded from the sensors	Retrieving the recorded data from the sensors	The total electricity consumption datasets regarding the total electricity consumption from the grid and the individual appliances specific consumptions recorded from the sensors	The two quarter-hourly preprocessed subsets (the total electricity consumption from the grid, the total electricity consumption of all the individual appliances, the electricity consumption for each of the individual appliances, the timestamps datasets): one for developing the forecasting ANNs and one for the final validation
	2. Preprocessing the data (filtering, reconstructing)	The acquired electricity consumption datasets regarding the total electricity consumption from the grid and the individual appliances specific consumptions recorded from the sensors	The preprocessed (filtered, reconstructed) quarter-hourly electricity consumption datasets	
	3. Constructing the timestamps dataset	The preprocessed quarter-hourly electricity consumption datasets from step 2	The quarter-hourly timestamps dataset	
	4. Obtaining the quarter-hourly preprocessed input datasets (the total electricity consumption from the grid, the total electricity consumption of all the individual appliances, the electricity consumption for each of the individual appliances, the timestamps datasets)	The preprocessed quarter-hourly electricity consumption datasets from step 2, the quarter-hourly timestamps dataset from step 3	The quarter-hourly preprocessed input datasets (the total electricity consumption from the grid, the total electricity consumption of all the individual appliances, the electricity consumption for each of the individual appliances, the timestamps datasets)	
	5. Dividing the datasets from step 4 into two subsets	The quarter-hourly preprocessed input datasets from step 4	Two quarter-hourly preprocessed subsets: one for developing the forecasting ANNs and one for the final validation	

Stage	Step	Input	Output	Final Results of the Stage
II. Developing the NARX ANN forecasting solution for:<br>Case 1: the total electricity consumption from the grid<br>Case 2: the total electricity consumption of all the individual appliances	1. Developing NARX ANNs based on the Levenberg-Marquardt algorithm for the two cases	The first quarter-hourly subset from the first stage:<br>Case 1:<br>• the timeseries - the total electricity consumption from the grid<br>• the exogenous variables – the timestamps dataset<br>Case 2:<br>• the timeseries - the total electricity consumption of all the individual appliances<br>• the exogenous variables – the timestamps dataset	Case 1:<br>A number of 36 trained NARX ANNs, using various settings regarding<br>the hidden number of neurons and the delay parameter, developed in order to forecast the total electricity consumption from the grid<br>Case 2:<br>A number of 36 trained NARX ANNs, using various settings regarding<br>the hidden number of neurons and the delay parameter, developed in order to forecast the total electricity consumption of all the individual appliances	The best NARX ANN forecasting solution for:<br>Case 1: the total electricity consumption from the grid<br>Case 2: the total electricity consumption of all the individual appliances
	2. Developing NARX ANNs based on the Bayesian Regularization algorithm for the two cases			
	3. Developing NARX ANNs based on the Scaled Conjugate Gradient algorithm for the two cases			
	4. Comparing the forecasting accuracy of the obtained ANNs for each of the two cases	Case 1:<br>The performance metrics of the 36 trained NARX ANNs from the steps 1,2,3<br>Case 2:<br>The performance metrics of the 36 trained NARX ANNs from the steps 1,2,3	Case 1:<br>The best NARX ANN out of the 36 developed ones in order to forecast the total electricity consumption from the grid<br>Case 2:<br>The best NARX ANN out of the 36 developed ones in order to forecast the total electricity consumption of all the individual appliances	

<table>
<tr><th>Stage</th><th>Step</th><th>Input</th><th>Output</th><th>Final Results of the Stage</th></tr>
<tr><td rowspan="4">III. Developing the FITNET ANN forecasting solution for the electricity consumption for each of the individual appliances in two cases, when using as input:
Case 1: the total electricity consumption from the grid;
Case 2: the total electricity consumption of all the individual appliances</td><td>1. Developing ANNs based on the Levenberg-Marquardt (LM) algorithm for the two cases</td><td rowspan="3">Case 1:
The first quarter-hourly subset from the first stage:
• training input – the total electricity consumption from the grid, the timestamps datasets
• training output – the electricity consumption for each of the individual appliances
Case 2:
The first quarter-hourly subset from the first stage:
• training input – the total electricity consumption of all the individual appliances, the timestamps datasets
• training output – the electricity consumption for each of the individual appliances</td><td rowspan="3">Case 1:
A number of 135 trained FITNET ANNs, using various settings regarding the hidden number of neurons, developed in order to forecast the electricity consumption dataset for each of the individual appliances
Case 2:
A number of 135 trained FITNET ANNs, using various settings regarding the hidden number of neurons, developed in order to forecast the electricity consumption dataset for each of the individual appliances</td><td rowspan="4">The best FITNET ANN forecasting solution for the electricity consumption for each of the individual appliances in two cases, when using as input:
Case 1. the total electricity consumption from the grid;
Case 2. the total electricity consumption of all the individual appliances</td></tr>
<tr><td>2. Developing ANNs based on the Bayesian Regularization (BR) algorithm for the two cases</td></tr>
<tr><td>3. Developing ANNs based on the Scaled Conjugate Gradient (SCG) algorithm for the two cases</td></tr>
<tr><td>4. Comparing the forecasting accuracy of the obtained ANNs for each of the two cases</td><td>Case 1:
The performance metrics of the 135 trained FITNET ANNs from the steps 1,2,3
Case 2:
The performance metrics of the 135 trained FITNET ANNs from the steps 1,2,3</td><td>Case 1:
The best FITNET ANN out of the 135 developed in order to forecast the electricity consumption dataset for each of the individual appliances
Case 2:
The best FITNET ANN out of the 135 developed in order to forecast the electricity consumption dataset for each of the individual appliances</td></tr>
</table>

Stage	Step	Input	Output	Final Results of the Stage
IV. Obtaining the forecast using the best mix of NARX and FITNET ANNs in view of validating the forecasting solution of all the individual appliances electricity consumption stemming from:<br>Case 1: the total electricity consumption from the grid;<br>Case 2: the total electricity consumption of all the individual appliances	1. Forecasting the total electricity consumption datasets using the closed loop form of the best ANNs forecasting solutions based on the NARX model (for the next month) in view of obtaining:<br>Case 1. the total electricity consumption from the grid;<br>Case 2. the total electricity consumption of all the individual appliances	• The second quarter-hourly subset from the first stage:<br>Case 1. the total electricity consumption from the grid;<br>Case 2. the total electricity consumption of all the individual appliances<br>• The second quarter-hourly subset of the timestamps dataset (used as exogenous variables)	Case 1. the forecasted total electricity consumption from the grid;<br>Case 2. the forecasted total electricity consumption of all the individual appliances	• The validated forecasting solution for the total electricity consumption for:<br>Case 1. the total electricity consumption from the grid;<br>Case 2. the total electricity consumption of all the individual appliances<br>• The validated forecasting solution for each of the individual appliances electricity consumption stemming from:<br>Case 1. The total electricity consumption from the grid and the second quarter-hourly subset from the first stage: the timestamps dataset<br>Case 2. The total electricity consumption of all the individual appliances and the second quarter-hourly subset from the first stage: the timestamps dataset
	2. Validating the forecasting solution for the total electricity consumption for:<br>Case 1. the total electricity consumption from the grid;<br>Case 2. the total electricity consumption of all the individual appliances	• Case 1. The forecasted total electricity consumption from the grid;<br>• Case 2. The forecasted total electricity consumption of all the individual appliances<br>• The second quarter-hourly subset from the first stage: the total electricity consumption electricity consumption from the grid and the total electricity consumption of all the individual appliances	The validation of the forecasting solution for the total electricity consumption for:<br>Case 1. the total electricity consumption from the grid;<br>Case 2. the total electricity consumption of all the individual appliances	
	3. Forecasting the consumption datasets for each of the individual appliances using the best ANN FITNET forecasting solution (for the next month) stemming from:<br>Case 1. The total electricity consumption from the grid;	• Case 1. The total electricity consumption from the grid and the second quarter-hourly subset from the first stage: the timestamps dataset<br>• Case 2. The total electricity consumption of all the individual appliances and the second quarter-hourly subset from the	The forecasted electricity consumption dataset for each of the individual appliances stemming from:<br>• Case 1. The total electricity consumption from the grid and the second quarter-hourly subset from the first stage: the timestamps dataset	

Case 2. The total electricity consumption of all the individual appliances	first stage: the timestamps dataset	• Case 2. The total electricity consumption of all the individual appliances and the second quarter-hourly subset from the first stage: the timestamps dataset
4. Validating the forecasting solution for each of the individual appliances electricity consumption stemming from: Case 1. The total electricity consumption from the grid; Case 2. The total electricity consumption of all the individual appliances	The forecasted electricity consumption dataset for each of the individual appliances stemming from: • Case 1. The total electricity consumption from the grid and the second quarter-hourly subset from the first stage: the timestamps dataset • Case 2. The total electricity consumption of all the individual appliances and the second quarter-hourly subset from the first stage: the timestamps dataset • The second quarter-hourly subset from the first stage: the consumption dataset for each of the individual appliances	The validation of the forecasting solution for each of the individual appliances electricity consumption stemming from: • Case 1. The total electricity consumption from the grid and the second quarter-hourly subset from the first stage: the timestamps dataset • Case 2. The total electricity consumption of all the individual appliances and the second quarter-hourly subset from the first stage: the timestamps dataset

Stage	Step	Input	Output	Final Results of the Stage
V. Compiling the developed method and incorporating it into the cloud designed solution	1. Compiling the validated ANN NARX and the validated ANN FITNET forecasting solutions in a Python package for the electricity consumption for each of the individual appliances stemming from: Case 1. The total electricity consumption from the grid; Case 2. The total electricity consumption of all the individual appliances	• The validated ANN NARX forecasting solutions for both cases<br>• The validated ANN FITNET forecasting solutions for both cases	The devised method, compiled as a Python package	The devised compiled forecasting method for the individual appliances' electricity consumption of residential consumers based on sensors recorded data, incorporated into our cloud designed solution
	2. Incorporating the forecasting method, compiled as a Python package, into our cloud designed solution	The devised method, compiled as a Python package	The devised method, compiled as a Python package, incorporated into our cloud designed solution	

Below are presented the experimental results registered for the Smart Homes 1-8, when developing the artificial neural networks forecasting solution for the total electricity consumption coming from the grid meter, based on the NARX model. In all the tables, the best obtained forecasting results are highlighted in red.

S2. The experimental results for the Smart Home 1, when developing the artificial neural networks forecasting solution for the total electricity consumption coming from the grid meter, based on the NARX model.

**The Levenberg-Marquardt Training Algorithm**					
n	***d***	**8**	**16**	**24**	**48**
6	MSE	0.00605157	0.00632257	0.00628395	0.006280224
	R	0.99115625	0.93192179	0.91609865	0.920099558
12	MSE	0.00587021	0.00635222	0.00616214	0.006110202
	R	0.99066451	0.96670423	0.99048373	0.978701934
24	MSE	0.00613578	0.00593968	0.00581619	0.005914398
	R	0.99712777	0.98739274	0.99964855	0.991774453
**The Bayesian Regularization Training Algorithm**					
n	***d***	**8**	**16**	**24**	**48**
6	MSE	0.00611032	0.00576593	0.0058297	0.005750613

	R	0.98740239	0.99153327	0.98480786	0.992161394
12	MSE	0.00595851	0.0059997	0.00584249	0.004934205
	R	0.99105479	0.98797547	0.99009159	0.993635819
24	MSE	0.005891	0.00590136	0.0055388	0.005593203
	R	0.99129307	0.99007086	0.99062987	0.99281961
**The Scaled Conjugate Gradient Training Algorithm**					
n	***d***	**8**	**16**	**24**	**48**
6	MSE	0.00571466	0.00598589	0.0062524	0.00639407
	R	0.99930172	0.98790575	0.99901011	0.984417105
12	MSE	0.00694051	0.00621034	0.00661947	0.006275528
	R	0.93943706	0.98384291	0.96256672	0.999001208
24	MSE	0.00638039	0.00658446	0.0068212	0.006177348
	R	0.95705274	0.97192389	0.93989853	0.965322145

S3. The experimental results for the Smart Home 2, when developing the artificial neural networks forecasting solution for the total electricity consumption coming from the grid meter, based on the NARX model.

**The Levenberg-Marquardt Training Algorithm**					
n	***d***	**8**	**16**	**24**	**48**
6	MSE	0.00629608	0.00677418	0.00673743	0.006869
	R	0.93449111	0.94143119	0.89721002	0.89193325
12	MSE	0.00624475	0.00674924	0.00654728	0.00629725
	R	0.9521921	0.92916232	0.90476879	0.94991658
24	MSE	0.00657405	0.00612151	0.00604809	0.0061558
	R	0.91083787	0.95835178	0.97024712	0.93050928
**The Bayesian Regularization Training Algorithm**					
n	***d***	**8**	**16**	**24**	**48**
6	MSE	0.00642529	0.00637287	0.00619405	0.00586679
	R	0.91071095	0.94764383	0.94618794	0.9589906
12	MSE	0.00625945	0.00636332	0.00613756	0.00593219
	R	0.92309667	0.95891737	0.96068293	0.94752749
24	MSE	0.00613393	0.00620245	0.00599561	0.00524584
	R	0.93575502	0.94184608	0.93723903	0.97249789
**The Scaled Conjugate Gradient Training Algorithm**					
n	***d***	**8**	**16**	**24**	**48**
6	MSE	0.00636001	0.00595031	0.0065076	0.00693252
	R	0.9320603	0.94954048	0.92474368	0.9094139
12	MSE	0.007302	0.00666317	0.00689244	0.00665974
	R	0.89293027	0.93654277	0.89714957	0.91439616

24	MSE	0.00678336	0.00705962	0.006959	0.00662314
	R	0.91952126	0.88781509	0.91198075	0.92709157

S4. The experimental results for the Smart Home 3, when developing the artificial neural networks forecasting solution for the total electricity consumption coming from the grid meter, based on the NARX model.

**The Levenberg-Marquardt Training Algorithm**					
n	***d***	**8**	**16**	**24**	**48**
6	MSE	0.00605157	0.00632257	0.00621917	0.006476481
	R	0.98266076	0.93192179	0.92554297	0.901322016
12	MSE	0.00599985	0.00641839	0.00635471	0.005923155
	R	0.99099007	0.98547519	0.99048373	0.997892168
24	MSE	0.00613578	0.00575786	0.0057937	0.005633025
	R	0.98754	0.99164337	0.99192495	0.998358915
**The Bayesian Regularization Training Algorithm**					
n	***d***	**8**	**16**	**24**	**48**
6	MSE	0.00623631	0.00600871	0.00601187	0.005692526
	R	0.98740239	0.99599301	0.99137728	0.990196752
12	MSE	0.00595851	0.00593909	0.00566544	0.005480209
	R	0.99111431	0.99073475	0.99195002	0.993635819
24	MSE	0.00583027	0.00596158	0.0054246	0.0050737
	R	0.99032837	0.99099293	0.99643307	0.99604010
**The Scaled Conjugate Gradient Training Algorithm**					
n	***d***	**8**	**16**	**24**	**48**
6	MSE	0.00617735	0.00617295	0.0061248	0.006461376
	R	0.99007384	0.96872312	0.98194432	0.984417105
12	MSE	0.00686822	0.00640441	0.00668772	0.006339564
	R	0.95803977	0.97438288	0.95322142	0.96202096
24	MSE	0.00638039	0.00672022	0.0068212	0.005832486
	R	0.97581848	0.95323304	0.9678163	0.99399508

S5. The experimental results for the Smart Home 4, when developing the artificial neural networks forecasting solution for the total electricity consumption coming from the grid meter, based on the NARX model.

**The Levenberg-Marquardt Training Algorithm**					
n	***d***	**8**	**16**	**24**	**48**
6	MSE	0.00580707	0.00612902	0.00634873	0.00634564
	R	0.98266076	0.96995941	0.97276455	0.96704341
12	MSE	0.00587741	0.00641839	0.00609796	0.00592316

	R	0.9810464	0.95731876	0.96191208	0.97870193
24	MSE	0.00613578	0.00600029	0.00573335	0.00581091
	R	0.96836447	0.98739274	0.99964855	0.99835892
**The Bayesian Regularization Training Algorithm**					
n	***d***	**8**	**16**	**24**	**48**
6	MSE	0.00598434	0.00588732	0.00601187	0.00557635
	R	0.98740239	0.99599301	0.99446284	0.99019675
12	MSE	0.00589833	0.0059997	0.00572446	0.00542371
	R	0.99008311	0.99183096	0.99009159	0.9916618
24	MSE	0.00601247	0.00596158	0.0054246	0.00498614
	R	0.98398982	0.99007086	0.99643307	0.9996615
**The Scaled Conjugate Gradient Training Algorithm**					
n	***d***	**8**	**16**	**24**	**48**
6	MSE	0.00630472	0.00604824	0.006061	0.00666329
	R	0.96532215	0.98790575	0.97241088	0.9469155
12	MSE	0.00708511	0.00621034	0.00675596	0.00608342
	R	0.95803977	0.97438288	0.94387611	0.97154592
24	MSE	0.00664904	0.00651658	0.0067523	0.00565574
	R	0.95705274	0.96257847	0.95851038	0.99105496

S6. The experimental results for the Smart Home 5, when developing the artificial neural networks forecasting solution for the total electricity consumption coming from the grid meter, based on the NARX model.

**The Levenberg-Marquardt Training Algorithm**					
n	***d***	**8**	**16**	**24**	**48**
6	MSE	0.00641834	0.00677418	0.00660787	0.00660732
	R	0.94412504	0.90339357	0.92554297	0.90132202
12	MSE	0.00642842	0.00688158	0.00667566	0.0064843
	R	0.942574	0.91977684	0.94286432	0.93072635
24	MSE	0.00651144	0.00612151	0.0059888	0.00621615
	R	0.91083787	0.93104522	0.94867146	0.94020209
**The Bayesian Regularization Training Algorithm**					
n	***d***	**8**	**16**	**24**	**48**
6	MSE	0.00642529	0.00619079	0.00637623	0.00604105
	R	0.92029738	0.95731367	0.95584292	0.95285147
12	MSE	0.00613907	0.00630271	0.00607855	0.0057062
	R	0.95224709	0.94923133	0.96068293	0.93765741
24	MSE	0.00631613	0.00632289	0.0057672	0.00540166
	R	0.92610806	0.9589906	0.9471047	0.96184608

**The Scaled Conjugate Gradient Training Algorithm**					
n	***d***	**8**	**16**	**24**	**48**
6	MSE	0.00653167	0.00654707	0.006699	0.00706713
	R	0.9048712	0.92076653	0.9056768	0.8906631
12	MSE	0.00751889	0.00679256	0.00709717	0.00600923
	R	0.88362892	0.91762272	0.88780426	0.94187146
24	MSE	0.00678336	0.00712751	0.0070279	0.00662314
	R	0.90075552	0.90650593	0.92128667	0.92709157

S7. The experimental results for the Smart Home 6, when developing the artificial neural networks forecasting solution for the total electricity consumption coming from the grid meter, based on the NARX model.

**The Levenberg-Marquardt Training Algorithm**					
n	***d***	**8**	**16**	**24**	**48**
6	MSE	0.00592932	0.00612902	0.00634873	0.00647648
	R	0.98266076	0.90339357	0.89721002	0.91071079
12	MSE	0.00599985	0.00628606	0.00635471	0.00604785
	R	0.99002826	0.95731876	0.96191208	0.96910682
24	MSE	0.00594795	0.00597475	0.00587021	0.00575786
	R	0.97795223	0.99707306	0.98984808	0.99835892
**The Bayesian Regularization Training Algorithm**					
n	***d***	**8**	**16**	**24**	**48**
6	MSE	0.00611032	0.00582662	0.00601187	0.00557635
	R	0.99065753	0.99153327	0.98480786	0.99314372
12	MSE	0.00583814	0.00593909	0.00584249	0.00542371
	R	0.99202647	0.99073475	0.99096974	0.9916618
24	MSE	0.00595174	0.00578093	0.0054246	0.00498614
	R	0.99129307	0.99201398	0.99260301	0.9985366
**The Scaled Conjugate Gradient Training Algorithm**					
n	***d***	**8**	**16**	**24**	**48**
6	MSE	0.00592354	0.0066145	0.0061248	0.00639407
	R	0.99301719	0.95851038	0.98194432	0.98441711
12	MSE	0.00686822	0.00633972	0.00668772	0.00633956
	R	0.93943706	0.99330294	0.98125734	0.99059584
24	MSE	0.00644755	0.00672022	0.00583249	0.00630472
	R	0.98520135	0.94388762	0.99749707	0.97487979

S8. The experimental results for the Smart Home 7, when developing the artificial neural networks forecasting solution for the total electricity consumption coming from the grid meter, based on the NARX model.

**The Levenberg-Marquardt Training Algorithm**					
n	***d***	**8**	**16**	**24**	**48**
6	MSE	0.00617383	0.00664514	0.00680222	0.00673816
	R	0.93449111	0.90339357	0.93498728	0.89193325
12	MSE	0.00618352	0.00694774	0.00648309	0.00629725
	R	0.9521921	0.92916232	0.92381655	0.94991658
24	MSE	0.00651144	0.00616668	0.00612151	0.0062765
	R	0.94918893	0.92931081	0.96044665	0.9595877
**The Bayesian Regularization Training Algorithm**					
n	***d***	**8**	**16**	**24**	**48**
6	MSE	0.00642529	0.00613009	0.00625478	0.00604105
	R	0.92029738	0.95731366	0.94618794	0.93320505
12	MSE	0.00613907	0.00630271	0.00607855	0.00593219
	R	0.95224709	0.94923133	0.93127427	0.94752749
24	MSE	0.00613393	0.00632289	0.0057672	0.00540166
	R	0.91646111	0.93269923	0.9471047	0.9688771
**The Scaled Conjugate Gradient Training Algorithm**					
n	***d***	**8**	**16**	**24**	**48**
6	MSE	0.0070968	0.00636000	0.006699	0.00686521
	R	0.90267482	0.93035784	0.9056768	0.9187893
12	MSE	0.00759119	0.00666317	0.00689244	0.00672378
	R	0.88362892	0.91762271	0.91584018	0.9048712
24	MSE	0.00705201	0.00699174	0.00600923	0.00655945
	R	0.91013839	0.92519677	0.96149378	0.92709157

S9. The experimental results for the Smart Home 8, when developing the artificial neural networks forecasting solution for the total electricity consumption coming from the grid meter, based on the NARX model.

**The Levenberg-Marquardt Training Algorithm**					
n	***d***	**8**	**16**	**24**	**48**
6	MSE	0.00580707	0.00625805	0.00615439	0.00641106
	R	0.97302683	0.91290298	0.91609865	0.92009956
12	MSE	0.00599985	0.00635222	0.00616214	0.0059855
	R	0.99099007	0.94793328	0.98095985	0.96910682
24	MSE	0.00613578	0.00581846	0.00573335	0.00587021
	R	0.99067155	0.99707306	0.99964855	0.98866611

**The Bayesian Regularization Training Algorithm**					
n	***d***	**8**	**16**	**24**	**48**
6	MSE	0.00598434	0.00582662	0.00576897	0.00575061
	R	0.98740239	0.98632317	0.99137728	0.99214431
12	MSE	0.00595851	0.00593909	0.00572446	0.0055932
	R	0.98139751	0.98797547	0.99084231	0.9906748
24	MSE	0.00576954	0.00596158	0.0055388	0.00514196
	R	0.97434286	0.99104242	0.99062987	0.99989447
**The Scaled Conjugate Gradient Training Algorithm**					
n	***d***	**8**	**16**	**24**	**48**
6	MSE	0.00630472	0.00592354	0.0063162	0.00659599
	R	0.97487979	0.99070884	0.96287744	0.9656663
12	MSE	0.00694051	0.00640441	0.00661947	0.00621149
	R	0.94873841	0.96492286	0.94387611	0.97154592
24	MSE	0.00644755	0.00651658	0.0067523	0.00571466
	R	0.95705274	0.98126931	0.93989853	0.99301719

S10. The comparison of the best experimental results recorded for Smart Homes 1-8 when developing the artificial neural networks forecasting solution for the total electricity consumption coming from the grid meter, based on the NARX model. The forecasting results of the Smart Home 3, that are presented in detail in the paper, are highlighted in red.

**The Best Forecasting Results**								
**The Smart Home number**	**1**	**2**	**3**	**4**	**5**	**6**	**7**	**8**
**The training algorithm**	BR	BR	BR	BR	BR	BR	BR	BR
**The number of neurons in the hidden layer n**	12	24	24	24	24	24	24	24
**The delay parameter d**	48	48	48	48	48	48	48	48
**MSE**	0.004934205	0.00524584	0.0050737	0.00498614	0.00540166	0.00498614	0.00540166	0.00514196
**R**	0.993635819	0.97249789	0.99604010	0.9996615	0.96184608	0.9985366	0.9688771	0.99989447

Below are presented the experimental results registered for the Smart Homes 1-8, when developing the FITNET ANNs forecasting solution using the total electricity consumption from the grid. In all the tables, the best obtained forecasting results are highlighted in red.

S11. The experimental results for the Smart Home 1, when developing the FITNET ANNs forecasting solution using the total electricity consumption from the grid.

n	**The Training Algorithm**	**The Levenberg-Marquardt Training Algorithm**	**The Bayesian Regularization Training Algorithm**	**The Scaled Conjugate Gradient Training Algorithm**
1	MSE	0.02945355	0.02759118	0.02892438
	R	0.95063958	0.89373558	0.93142698

2	MSE	0.02845676	0.02708888	0.02823666
	R	0.93225148	0.92268768	0.94115378
3	MSE	0.02586105	0.02638896	0.02927085
	R	0.94224844	0.89441169	0.89308923
4	MSE	0.02584504	0.0261449	0.02632552
	R	0.94310594	0.94330096	0.9045714
5	MSE	0.02445614	0.02420869	0.02683871
	R	0.93431855	0.93466193	0.9418633
6	MSE	0.0240873	0.02450175	0.02706392
	R	0.90666666	0.95651523	0.92276544
7	MSE	0.02438205	0.02423505	0.0245463
	R	0.90860306	0.92914464	0.95381154
8	MSE	0.02416274	0.02328165	0.02556916
	R	0.95893281	0.93249504	0.91376225
9	MSE	0.02377112	0.0233163	0.02416586
	R	0.94175748	0.91301354	0.95518467
10	MSE	0.02219622	0.02098477	0.0250929
	R	0.90381027	0.93445344	0.9154295
11	MSE	0.02256384	0.02230558	0.02743052
	R	0.93308448	0.91470272	0.91328155
12	MSE	0.02167561	0.02197624	0.02493849
	R	0.91398268	0.9341616	0.91804675
13	MSE	0.02178724	0.02088882	0.0247988
	R	0.93479808	0.95423188	0.94457398
14	MSE	0.02111928	0.02179376	0.0241415
	R	0.96424218	0.93494016	0.93929659
15	MSE	0.02092171	0.02014783	0.02314482
	R	0.90640032	0.90632034	0.94922114
16	MSE	0.02054535	0.02026731	0.02501339
	R	0.91584106	0.96449958	0.90799488
17	MSE	0.0190859	0.0204845	0.02388026
	R	0.95946018	0.9262557	0.94145872
18	MSE	0.01979432	0.02052584	0.02505619
	R	0.94635916	0.93569088	0.93637495
19	MSE	0.01988584	0.01993215	0.02257668
	R	0.90677232	0.95637024	0.9520112
20	MSE	0.01917906	0.01934649	0.02548208
	R	0.9178583	0.96685776	0.9147854
21	MSE	0.01919714	0.01879913	0.02477538

	R	0.90981807	0.94941175	0.95645385
22	MSE	0.01980992	0.01899456	0.0241226
	R	0.91693522	0.96141332	0.945798
23	MSE	0.01881529	0.01953156	0.02391771
	R	0.91005894	0.91184547	0.9215323
24	MSE	0.01978066	0.01946568	0.02332065
	R	0.93820032	0.90848538	0.94238119
25	MSE	0.01948386	0.0197415	0.0236292
	R	0.92215692	0.92065668	0.9305328
26	MSE	0.01969656	0.01817334	0.02328976
	R	0.97073658	0.97204239	0.90183774
27	MSE	0.0188466	0.0187257	0.02255424
	R	0.928739	0.91297077	0.9229269
28	MSE	0.01898884	0.01901818	0.02440884
	R	0.96167596	0.93323345	0.91004784
29	MSE	0.01848392	0.01784973	0.02273106
	R	0.96225612	0.96371534	0.900984
30	MSE	0.01886731	0.01838616	0.02437995
	R	0.96382902	0.91412304	0.95711022
31	MSE	0.01897294	0.01778778	0.02406022
	R	0.94213632	0.95463908	0.89700174
32	MSE	0.01833588	0.0178398	0.0236082
	R	0.9552172	0.97375509	0.92088345
33	MSE	0.01789316	0.01796118	0.02343559
	R	0.93361155	0.9540435	0.93945664
34	MSE	0.01860283	0.01762662	0.02432325
	R	0.96148976	0.96532646	0.95712804
35	MSE	0.01744511	0.01787656	0.0231338
	R	0.95742589	0.97527672	0.90164244
36	MSE	0.0185598	0.01747805	0.02431416
	R	0.93421005	0.95511923	0.926928
37	MSE	0.01833728	0.0177788	0.02167258
	R	0.91466802	0.94604064	0.96274827
38	MSE	0.01747515	0.01732352	0.02436834
	R	0.93010086	0.92613782	0.9196741
39	MSE	0.01835708	0.01774656	0.02303911
	R	0.95487576	0.95672555	0.93917049
40	MSE	0.01765935	0.01712784	0.02361504
	R	0.96565672	0.91979325	0.94751888
41	MSE	0.01756144	0.0181686	0.0223613

	R	0.91840476	0.91739571	0.95240222
42	MSE	0.01814292	0.0171411	0.02386098
	R	0.91767006	0.97893378	0.95712507
43	MSE	0.01699123	0.01681246	0.02377549
	R	0.9386969	0.97949412	0.9095205
44	MSE	0.01760899	0.01768292	0.02336029
	R	0.9343694	0.95942991	0.93795799
45	MSE	0.01793427	0.01688013	0.02198063
	R	0.9396013	0.93963265	0.96204141

S12. The experimental results for the Smart Home 2, when developing the FITNET ANNs forecasting solution using the total electricity consumption from the grid.

n	The Training Algorithm	The Levenberg-Marquardt Training Algorithm	The Bayesian Regularization Training Algorithm	The Scaled Conjugate Gradient Training Algorithm
1	MSE	0.02861202	0.0286839	0.0300699
	R	0.95063958	0.9129557	0.93142698
2	MSE	0.02845676	0.02734935	0.02795983
	R	0.92264064	0.92268768	0.94115378
3	MSE	0.02586105	0.02562774	0.02982839
	R	0.90378932	0.89441169	0.94110478
4	MSE	0.02634206	0.02540495	0.02607239
	R	0.93348241	0.92404992	0.9334407
5	MSE	0.02469828	0.02444838	0.02631757
	R	0.9054221	0.95393331	0.9226416
6	MSE	0.0250319	0.0238017	0.02732415
	R	0.90666666	0.93719169	0.90354116
7	MSE	0.02391763	0.02354262	0.02526825
	R	0.90860306	0.90978746	0.89600478
8	MSE	0.02325946	0.02328165	0.02556916
	R	0.92018805	0.94220853	0.93299935
9	MSE	0.02266032	0.02265012	0.0246351
	R	0.96117516	0.91301354	0.95518467
10	MSE	0.02328427	0.02181585	0.0250929
	R	0.93296544	0.90525177	0.9443378
11	MSE	0.02299776	0.02146386	0.02640508
	R	0.93308448	0.9244336	0.90366806
12	MSE	0.02210483	0.02197624	0.02493849
	R	0.94315234	0.90496905	0.9470377
13	MSE	0.02137616	0.0217161	0.0252757

	R	0.90558564	0.90554658	0.93493547
14	MSE	0.02152542	0.02179376	0.023686
	R	0.9252829	0.90572328	0.92961312
15	MSE	0.01974853	0.01995222	0.02291791
	R	0.93563904	0.91606572	0.94922114
16	MSE	0.02074102	0.01987377	0.02454585
	R	0.96455601	0.90604506	0.90799488
17	MSE	0.0194565	0.0197115	0.0234339
	R	0.94966977	0.94575582	0.96087024
18	MSE	0.01922333	0.01994492	0.02458785
	R	0.96587172	0.90645054	0.89776155
19	MSE	0.02026826	0.01993215	0.0232407
	R	0.91652256	0.90757584	0.9422968
20	MSE	0.01936709	0.01915866	0.02499204
	R	0.9373872	0.95709152	0.90515608
21	MSE	0.01994266	0.01972978	0.02454165
	R	0.91960106	0.939624	0.9274704
22	MSE	0.01961944	0.01935984	0.0248252
	R	0.94619911	0.96141332	0.926496
23	MSE	0.01881529	0.01897878	0.02391771
	R	0.91984452	0.96086942	0.9215323
24	MSE	0.01903422	0.01909134	0.0222309
	R	0.9284274	0.95732868	0.93266592
25	MSE	0.01966767	0.0186345	0.02317912
	R	0.95158746	0.9304509	0.92083975
26	MSE	0.01914432	0.01852968	0.02306582
	R	0.91190406	0.96222378	0.93092928
27	MSE	0.0190332	0.0187257	0.02343872
	R	0.9189628	0.91297077	0.96178698
28	MSE	0.01863056	0.01812948	0.02418072
	R	0.96167596	0.94305696	0.94877328
29	MSE	0.01848392	0.01784973	0.02385636
	R	0.9327993	0.96371534	0.939736
30	MSE	0.01851465	0.01838616	0.02414776
	R	0.95399403	0.9337816	0.90877132
31	MSE	0.01807799	0.01848534	0.02429844
	R	0.94213632	0.95463908	0.94522764
32	MSE	0.01764396	0.0181896	0.02293368
	R	0.9256744	0.96391918	0.94027047
33	MSE	0.01842464	0.01796118	0.02320806

	R	0.93361155	0.944208	0.94914176
34	MSE	0.01896405	0.01762662	0.02478655
	R	0.96148976	0.97517673	0.9184562
35	MSE	0.01744511	0.01770467	0.0231338
	R	0.96729626	0.91616904	0.91133752
36	MSE	0.01838304	0.01817025	0.02408037
	R	0.93421005	0.91573287	0.926928
37	MSE	0.01833728	0.01794975	0.02188716
	R	0.97367886	0.96574982	0.93357408
38	MSE	0.01747515	0.01749504	0.02321889
	R	0.93010086	0.95569541	0.90031254
39	MSE	0.01783754	0.01808784	0.02417966
	R	0.94503168	0.93699925	0.91012398
40	MSE	0.0181737	0.01712784	0.02477264
	R	0.91638852	0.949464	0.9185132
41	MSE	0.01739258	0.017829	0.0219271
	R	0.91840476	0.96671806	0.94268383
42	MSE	0.01746468	0.01788291	0.02339766
	R	0.91767006	0.97893378	0.91845335
43	MSE	0.01800061	0.01764476	0.02331383
	R	0.91893486	0.9399186	0.95789925
44	MSE	0.01760899	0.01718246	0.02451674
	R	0.9641897	0.93964785	0.92828832
45	MSE	0.01793427	0.01697305	0.02198063
	R	0.92971076	0.97919613	0.93288864

S13. The experimental results for the Smart Home 3, when developing the FITNET ANNs forecasting solution using the total electricity consumption from the grid.

n	The Training Algorithm	The Levenberg-Marquardt Training Algorithm	The Bayesian Regularization Training Algorithm	The Scaled Conjugate Gradient Training Algorithm
1	MSE	0.02889253	0.02841072	0.02892438
	R	0.9122299	0.94178588	0.90261996
2	MSE	0.0281883	0.02708888	0.02823666
	R	0.9130298	0.90346502	0.89313573
3	MSE	0.0261171	0.02613522	0.02815577
	R	0.89417454	0.91364635	0.90269234
4	MSE	0.02534802	0.02639155	0.02581926
	R	0.90461182	0.90479888	0.9238176
5	MSE	0.02445614	0.02492776	0.02657814

	R	0.9246864	0.93466193	0.91303075
6	MSE	0.02385115	0.02403505	0.02628323
	R	0.90666666	0.91786815	0.90354116
7	MSE	0.02345321	0.02446586	0.02526825
	R	0.91826905	0.93882323	0.9152737
8	MSE	0.02325946	0.02261646	0.02556916
	R	0.92018805	0.96163551	0.9426179
9	MSE	0.02377112	0.02242806	0.02369662
	R	0.91263096	0.96157809	0.89729469
10	MSE	0.02219622	0.02098477	0.02557086
	R	0.93296544	0.93445344	0.9057934
11	MSE	0.02234688	0.02251601	0.02589236
	R	0.95252374	0.90497184	0.92289504
12	MSE	0.02274866	0.02197624	0.02447235
	R	0.93342912	0.92443075	0.9083831
13	MSE	0.0215817	0.02130246	0.02408345
	R	0.9250606	0.95423188	0.90601994
14	MSE	0.02111928	0.02159008	0.0232305
	R	0.90580326	0.95441808	0.90056271
15	MSE	0.02092171	0.02073466	0.02314482
	R	0.95513152	0.91606572	0.92016335
16	MSE	0.01976267	0.01987377	0.02454585
	R	0.96455601	0.96449958	0.9176544
17	MSE	0.0194565	0.02067775	0.0234339
	R	0.92029854	0.90675558	0.94238119
18	MSE	0.01922333	0.01955764	0.02482202
	R	0.96587172	0.94543766	0.95568165
19	MSE	0.01950342	0.02012198	0.0232407
	R	0.96527376	0.95637024	0.9034392
20	MSE	0.01993118	0.01934649	0.02474702
	R	0.9373872	0.9277928	0.93404404
21	MSE	0.01901076	0.01991591	0.02407419
	R	0.95873302	0.939624	0.89848695
22	MSE	0.01942896	0.0191772	0.0241226
	R	0.93644448	0.9319823	0.907194
23	MSE	0.01993303	0.01897878	0.02369418
	R	0.91005894	0.91184547	0.93123264
24	MSE	0.01922083	0.01909134	0.02469881
	R	0.96751908	0.95732868	0.94984475
25	MSE	0.01874862	0.018819	0.02317912

	R	0.94177728	0.91086246	0.9305328
26	MSE	0.0193284	0.01835151	0.02373764
	R	0.95112574	0.96222378	0.9212321
27	MSE	0.019593	0.01819068	0.0232176
	R	0.928739	0.92278766	0.93264192
28	MSE	0.01863056	0.01848496	0.02440884
	R	0.92242388	0.92340994	0.94877328
29	MSE	0.01901711	0.01784973	0.02295612
	R	0.97207506	0.95388151	0.930048
30	MSE	0.01851465	0.01856295	0.02414776
	R	0.95399403	0.91412304	0.93777466
31	MSE	0.01843597	0.01813656	0.0250131
	R	0.97157808	0.9343694	0.94522764
32	MSE	0.01798992	0.0183645	0.02338336
	R	0.9256744	0.93441145	0.94027047
33	MSE	0.01895612	0.01813727	0.02298053
	R	0.97292151	0.9540435	0.91040128
34	MSE	0.01914466	0.01849067	0.02432325
	R	0.96148976	0.95547619	0.90878824
35	MSE	0.01812259	0.01822034	0.0238076
	R	0.93768515	0.95557416	0.95011784
36	MSE	0.01838304	0.01782415	0.02501553
	R	0.91454247	0.91573287	0.9172725
37	MSE	0.01798464	0.01760785	0.0225309
	R	0.9343383	0.94604064	0.93357408
38	MSE	0.01730872	0.01732352	0.02344878
	R	0.95978493	0.96554794	0.94871644
39	MSE	0.0181839	0.01757592	0.02395155
	R	0.94503168	0.91727295	0.91980615
40	MSE	0.0181737	0.01779952	0.02454112
	R	0.9360958	0.92975682	0.9185132
41	MSE	0.01705486	0.0171498	0.0225784
	R	0.95790604	0.96671806	0.94268383
42	MSE	0.01763424	0.0178133	0.02409264
	R	0.91767006	0.94926912	0.93778921
43	MSE	0.01783238	0.01714538	0.0222309
	R	0.96833996	0.94981248	0.96087024
44	MSE	0.01695071	0.01784974	0.02405416
	R	0.97924101	0.949464	0.95729733
45	MSE	0.01793427	0.01771578	0.02328641

	R	0.92971076	0.95941439	0.94260623

S14. The experimental results for the Smart Home 4, when developing the FITNET ANNs forecasting solution using the total electricity consumption from the grid.

n	The Training Algorithm	The Levenberg-Marquardt Training Algorithm	The Bayesian Regularization Training Algorithm	The Scaled Conjugate Gradient Training Algorithm
1	MSE	0.02917304	0.02895708	0.03064266
	R	0.94103716	0.89373558	0.95063166
2	MSE	0.02791984	0.02708888	0.02906715
	R	0.95147316	0.91307635	0.92194656
3	MSE	0.0266292	0.02638896	0.02927085
	R	0.90378932	0.91364635	0.93150167
4	MSE	0.02559653	0.02540495	0.02581926
	R	0.91423535	0.93367544	0.9526869
5	MSE	0.02445614	0.02420869	0.02709928
	R	0.9054221	0.93466193	0.91303075
6	MSE	0.02526805	0.0238017	0.02628323
	R	0.92595744	0.90820638	0.94198972
7	MSE	0.02438205	0.02377343	0.02430565
	R	0.90860306	0.93882323	0.9152737
8	MSE	0.02348528	0.02350338	0.02556916
	R	0.92018805	0.95192202	0.9426179
9	MSE	0.02266032	0.02287218	0.02393124
	R	0.95146632	0.96157809	0.92623968
10	MSE	0.02241383	0.02202362	0.02413698
	R	0.93296544	0.90525177	0.9539739
11	MSE	0.02299776	0.02188472	0.02666144
	R	0.90392559	0.93416448	0.95173551
12	MSE	0.02253405	0.02134231	0.02423928
	R	0.91398268	0.90496905	0.9083831
13	MSE	0.02075954	0.02212974	0.02456035
	R	0.95427304	0.91528364	0.94457398
14	MSE	0.02172849	0.0213864	0.02300275
	R	0.93502272	0.90572328	0.95866353
15	MSE	0.01974853	0.01975661	0.02405246
	R	0.93563904	0.93555648	0.95890707
16	MSE	0.02034968	0.02066085	0.02384454
	R	0.90609807	0.91578748	0.93697344
17	MSE	0.0196418	0.01990475	0.02254118

	R	0.95946018	0.9262557	0.93175296
18	MSE	0.01941366	0.01955764	0.02388534
	R	0.9268466	0.95518444	0.9267216
19	MSE	0.01931221	0.01936266	0.02235534
	R	0.94577328	0.90757584	0.9617256
20	MSE	0.01899103	0.01972215	0.02597212
	R	0.9373872	0.95709152	0.94367336
21	MSE	0.01994266	0.01879913	0.02430792
	R	0.90981807	0.96898725	0.89848695
22	MSE	0.01980992	0.01899456	0.0248252
	R	0.96570837	0.94179264	0.907194
23	MSE	0.01993303	0.01953156	0.02257653
	R	0.91984452	0.96086942	0.95063332
24	MSE	0.01940744	0.02002719	0.02244885
	R	0.95774616	0.96709734	0.92295065
25	MSE	0.01893243	0.0186345	0.02340416
	R	0.96139764	0.9304509	0.92083975
26	MSE	0.01914432	0.01906419	0.02306582
	R	0.9315149	0.95240517	0.96002082
27	MSE	0.0188466	0.01819068	0.02365984
	R	0.9482914	0.96205522	0.96178698
28	MSE	0.01809314	0.01795174	0.02349636
	R	0.97148898	0.97252749	0.93909192
29	MSE	0.01795073	0.01873338	0.02385636
	R	0.91316142	0.92438002	0.930048
30	MSE	0.01886731	0.01820937	0.02345119
	R	0.97366401	0.94361088	0.9184391
31	MSE	0.01843597	0.01796217	0.02548954
	R	0.94213632	0.9349558	0.9162921
32	MSE	0.01798992	0.0187143	0.02270884
	R	0.9749124	0.92457554	0.93057696
33	MSE	0.01824748	0.01866554	0.02298053
	R	0.96309402	0.9737145	0.92977152
34	MSE	0.01896405	0.01831786	0.0240916
	R	0.92224528	0.95547619	0.92812416
35	MSE	0.01710637	0.01753278	0.0233584
	R	0.96729626	0.97527672	0.95981292
36	MSE	0.01873656	0.0183433	0.02431416
	R	0.96371142	0.94527264	0.946239
37	MSE	0.01780832	0.0181207	0.02188716

	R	0.97367886	0.92633146	0.93357408
38	MSE	0.01714229	0.01732352	0.02413845
	R	0.9741298	0.92613782	0.90031254
39	MSE	0.01766436	0.01774656	0.02326722
	R	0.97456392	0.95672555	0.92948832
40	MSE	0.0174879	0.01695992	0.02407808
	R	0.91638852	0.95935425	0.9185132
41	MSE	0.01756144	0.0176592	0.0225784
	R	0.97765668	0.97658253	0.90381027
42	MSE	0.0178038	0.0171411	0.0243243
	R	0.95713974	0.94926912	0.90878542
43	MSE	0.01800061	0.0174783	0.02354466
	R	0.94857792	0.92013084	0.9482235
44	MSE	0.01662157	0.0175161	0.02451674
	R	0.97957431	0.97921197	0.91861865
45	MSE	0.01776666	0.01788291	0.02306878
	R	0.96927292	0.95941439	0.93288864

S15. The experimental results for the Smart Home 5, when developing the FITNET ANNs forecasting solution using the total electricity consumption from the grid.

n	The Training Algorithm	The Levenberg-Marquardt Training Algorithm	The Bayesian Regularization Training Algorithm	The Scaled Conjugate Gradient Training Algorithm
1	MSE	0.03001457	0.02895708	0.02949714
	R	0.95063958	0.94178588	0.89301762
2	MSE	0.02845676	0.02630747	0.02962081
	R	0.90341896	0.95152167	0.92194656
3	MSE	0.0261171	0.02562774	0.02899208
	R	0.89417454	0.91364635	0.94110478
4	MSE	0.02659057	0.02639155	0.02581926
	R	0.93348241	0.93367544	0.9430638
5	MSE	0.0254247	0.02516745	0.02762042
	R	0.93431855	0.89611917	0.9226416
6	MSE	0.02385115	0.0238017	0.02758438
	R	0.94524822	0.92752992	0.90354116
7	MSE	0.02461426	0.02423505	0.0245463
	R	0.90860306	0.92914464	0.93454262
8	MSE	0.02416274	0.02328165	0.0265818
	R	0.92987424	0.95192202	0.9041437
9	MSE	0.0233268	0.0233163	0.0246351

	R	0.91263096	0.94215227	0.92623968
10	MSE	0.02219622	0.02098477	0.02413698
	R	0.93296544	0.96365511	0.9443378
11	MSE	0.0227808	0.02167429	0.02743052
	R	0.95252374	0.93416448	0.94212202
12	MSE	0.02167561	0.02197624	0.02354007
	R	0.94315234	0.9146999	0.89871945
13	MSE	0.02137616	0.02192292	0.02408345
	R	0.91532312	0.9250207	0.92529696
14	MSE	0.02111928	0.02057168	0.02300275
	R	0.94476254	0.96415704	0.91992965
15	MSE	0.02092171	0.01995222	0.02405246
	R	0.94538528	0.96479262	0.92016335
16	MSE	0.02015401	0.02026731	0.02407831
	R	0.96455601	0.95475716	0.93697344
17	MSE	0.0189006	0.02067775	0.02388026
	R	0.91050813	0.9262557	0.96087024
18	MSE	0.01979432	0.01955764	0.02505619
	R	0.91709032	0.95518444	0.9267216
19	MSE	0.02045947	0.01974232	0.02346204
	R	0.90677232	0.94661136	0.9617256
20	MSE	0.02011921	0.01953432	0.02474702
	R	0.90809385	0.95709152	0.93404404
21	MSE	0.01882438	0.01954365	0.02477538
	R	0.90981807	0.92983625	0.9081481
22	MSE	0.02019088	0.01899456	0.0241226
	R	0.95595374	0.9319823	0.936147
23	MSE	0.01993303	0.01897878	0.02280006
	R	0.91005894	0.97067421	0.90213162
24	MSE	0.01922083	0.01965285	0.02201295
	R	0.91865448	0.96709734	0.90352011
25	MSE	0.01893243	0.0186345	0.02317912
	R	0.95158746	0.96962778	0.94022585
26	MSE	0.01877616	0.01835151	0.02373764
	R	0.94132032	0.92294934	0.94062646
27	MSE	0.0188466	0.01801234	0.0232176
	R	0.9189628	0.93260455	0.91321188
28	MSE	0.01845142	0.01812948	0.0239526
	R	0.91261086	0.93323345	0.94877328
29	MSE	0.01866165	0.01820319	0.02295612

	R	0.94261824	0.97354917	0.949424
30	MSE	0.01816199	0.01856295	0.02461214
	R	0.93432405	0.95344016	0.93777466
31	MSE	0.01861496	0.01831095	0.02525132
	R	0.97157808	0.92511416	0.94522764
32	MSE	0.01833588	0.0183645	0.02270884
	R	0.9256744	0.92457554	0.91118994
33	MSE	0.0186018	0.01813727	0.02343559
	R	0.96309402	0.9540435	0.9200864
34	MSE	0.01860283	0.01831786	0.02385995
	R	0.95167864	0.96532646	0.95712804
35	MSE	0.01744511	0.01804845	0.0238076
	R	0.91794441	0.97527672	0.95011784
36	MSE	0.01838304	0.0183433	0.02361279
	R	0.97354521	0.92557946	0.907617
37	MSE	0.01780832	0.01726595	0.02188716
	R	0.91466802	0.95589523	0.95302354
38	MSE	0.01780801	0.01818112	0.02436834
	R	0.93963265	0.96554794	0.93903566
39	MSE	0.0181839	0.01774656	0.02440777
	R	0.9351876	0.93699925	0.92948832
40	MSE	0.0174879	0.01729576	0.02384656
	R	0.91638852	0.949464	0.89917608
41	MSE	0.01806802	0.0176592	0.0221442
	R	0.96778136	0.94698912	0.95240222
42	MSE	0.01746468	0.01697305	0.02455596
	R	0.91767006	0.91960446	0.89911749
43	MSE	0.01732769	0.01781122	0.02377549
	R	0.92881588	0.93002472	0.93854775
44	MSE	0.01711528	0.01734928	0.02336029
	R	0.9542496	0.95942991	0.93795799
45	MSE	0.01793427	0.01688013	0.02198063
	R	0.91982022	0.97957431	0.95232382

S16. The experimental results for the Smart Home 6, when developing the FITNET ANNs forecasting solution using the total electricity consumption from the grid.

n	The Training Algorithm	The Levenberg-Marquardt Training Algorithm	The Bayesian Regularization Training Algorithm	The Scaled Conjugate Gradient Training Algorithm
1	MSE	0.03001457	0.02759118	0.02921076

	R	0.92183232	0.95139594	0.92182464
2	MSE	0.02738292	0.02630747	0.02823666
	R	0.92264064	0.90346502	0.90273934
3	MSE	0.0266292	0.02689644	0.02815577
	R	0.9134041	0.91364635	0.91229545
4	MSE	0.02559653	0.02639155	0.02632552
	R	0.90461182	0.95292648	0.9526869
5	MSE	0.0254247	0.02468807	0.02631757
	R	0.9054221	0.91539055	0.9034199
6	MSE	0.0240873	0.0238017	0.02654346
	R	0.95489361	0.93719169	0.90354116
7	MSE	0.02438205	0.02354262	0.0250276
	R	0.90860306	0.94850182	0.90563924
8	MSE	0.02348528	0.02239473	0.0265818
	R	0.92987424	0.93249504	0.89452515
9	MSE	0.02243816	0.02287218	0.0246351
	R	0.91263096	0.91301354	0.90694302
10	MSE	0.02328427	0.02098477	0.02437596
	R	0.95240222	0.93445344	0.9057934
11	MSE	0.02299776	0.02251601	0.02614872
	R	0.92336485	0.96335712	0.89405457
12	MSE	0.02189022	0.02134231	0.02447235
	R	0.93342912	0.9536233	0.89871945
13	MSE	0.02137616	0.0217161	0.02408345
	R	0.94453556	0.95423188	0.95421249
14	MSE	0.02152542	0.02159008	0.02345825
	R	0.94476254	0.96415704	0.94898006
15	MSE	0.01994406	0.02093027	0.02291791
	R	0.90640032	0.90632034	0.95890707
16	MSE	0.02015401	0.02046408	0.02361077
	R	0.90609807	0.94501474	0.9176544
17	MSE	0.0189006	0.01990475	0.02388026
	R	0.91050813	0.9262557	0.9220472
18	MSE	0.01998465	0.0203322	0.02482202
	R	0.94635916	0.93569088	0.93637495
19	MSE	0.01988584	0.01993215	0.02257668
	R	0.96527376	0.9270936	0.9325824
20	MSE	0.01993118	0.01990998	0.02621714
	R	0.9178583	0.9277928	0.9147854
21	MSE	0.0195699	0.01898526	0.02477538

	R	0.92938405	0.939624	0.89848695
22	MSE	0.01961944	0.01862928	0.0238884
	R	0.90718059	0.94179264	0.945798
23	MSE	0.01918787	0.01971582	0.02302359
	R	0.94920126	0.91184547	0.96033366
24	MSE	0.01884761	0.01927851	0.02332065
	R	0.94797324	0.96709734	0.93266592
25	MSE	0.01856481	0.0186345	0.02340416
	R	0.9319671	0.96962778	0.94022585
26	MSE	0.0193284	0.01817334	0.02306582
	R	0.96093116	0.97204239	0.91153492
27	MSE	0.0199662	0.01836902	0.0232176
	R	0.9385152	0.97187211	0.94235694
28	MSE	0.01845142	0.01848496	0.02372448
	R	0.96167596	0.92340994	0.94877328
29	MSE	0.01830619	0.01855665	0.02385636
	R	0.97207506	0.95388151	0.939736
30	MSE	0.01833832	0.01820937	0.02391557
	R	0.93432405	0.94361088	0.9184391
31	MSE	0.01879395	0.01848534	0.02548954
	R	0.95195024	0.91527252	0.95487282
32	MSE	0.01781694	0.0187143	0.02293368
	R	0.9453696	0.93441145	0.93057696
33	MSE	0.01895612	0.01813727	0.02411818
	R	0.92378406	0.9737145	0.92977152
34	MSE	0.01860283	0.01779943	0.02478655
	R	0.97130088	0.97517673	0.94746008
35	MSE	0.01812259	0.01839223	0.0226846
	R	0.97716663	0.97527672	0.94042276
36	MSE	0.01785276	0.0179972	0.02501553
	R	0.96371142	0.96496582	0.907617
37	MSE	0.0185136	0.01829165	0.0225309
	R	0.94417344	0.91647687	0.92384935
38	MSE	0.01730872	0.01749504	0.02413845
	R	0.96967962	0.91628529	0.90999332
39	MSE	0.01766436	0.01757592	0.02303911
	R	0.97456392	0.9271361	0.94885266
40	MSE	0.01800225	0.0171411	0.0243096
	R	0.96565672	0.91979325	0.93785032
41	MSE	0.01722372	0.0174894	0.0221442

	R	0.94803072	0.92726018	0.92324705
42	MSE	0.01746468	0.01701564	0.02409264
	R	0.9374049	0.96932094	0.94745714
43	MSE	0.01732769	0.01697892	0.02354466
	R	0.96833996	0.93002472	0.93854775
44	MSE	0.01744442	0.01695992	0.02405416
	R	0.9443095	0.97893378	0.90894898
45	MSE	0.01759905	0.01704726	0.02241589
	R	0.94949184	0.92974178	0.91345346

S17. The experimental results for the Smart Home 7, when developing the FITNET ANNs forecasting solution using the total electricity consumption from the grid.

n	The Training Algorithm	The Levenberg-Marquardt Training Algorithm	The Bayesian Regularization Training Algorithm	The Scaled Conjugate Gradient Training Algorithm
1	MSE	0.03001457	0.02895708	0.03035628
	R	0.9122299	0.9129557	0.90261996
2	MSE	0.02845676	0.02630747	0.02879032
	R	0.92264064	0.89385369	0.90273934
3	MSE	0.0266292	0.02588148	0.02982839
	R	0.89417454	0.94249834	0.93150167
4	MSE	0.02609355	0.02491165	0.02581926
	R	0.93348241	0.93367544	0.9334407
5	MSE	0.02469828	0.02444838	0.02709928
	R	0.95358285	0.90575486	0.89380905
6	MSE	0.0245596	0.0238017	0.02654346
	R	0.89702127	0.93719169	0.89392902
7	MSE	0.02461426	0.02446586	0.02526825
	R	0.93760103	0.90010887	0.9152737
8	MSE	0.0237111	0.02261646	0.02556916
	R	0.93956043	0.93249504	0.93299935
9	MSE	0.02266032	0.02376042	0.02510434
	R	0.94175748	0.93243936	0.89729469
10	MSE	0.02306666	0.02181585	0.02461494
	R	0.95240222	0.96365511	0.9443378
11	MSE	0.02299776	0.02146386	0.02743052
	R	0.96224337	0.91470272	0.94212202
12	MSE	0.02253405	0.02261017	0.02400621
	R	0.93342912	0.9536233	0.95670135
13	MSE	0.02199278	0.02192292	0.0252757

	R	0.93479808	0.91528364	0.89638143
14	MSE	0.02132235	0.0213864	0.02436925
	R	0.95450236	0.93494016	0.92961312
15	MSE	0.01974853	0.02073466	0.02337173
	R	0.93563904	0.91606572	0.95890707
16	MSE	0.02054535	0.01987377	0.02431208
	R	0.93532704	0.93527232	0.9176544
17	MSE	0.0189006	0.02067775	0.02276436
	R	0.94966977	0.95550588	0.91234144
18	MSE	0.01922333	0.01975128	0.02411951
	R	0.95611544	0.94543766	0.93637495
19	MSE	0.02026826	0.01955249	0.02235534
	R	0.95552352	0.91733472	0.9034392
20	MSE	0.01974315	0.01953432	0.02474702
	R	0.96668055	0.9277928	0.9147854
21	MSE	0.01919714	0.01935752	0.02360673
	R	0.93916704	0.96898725	0.93713155
22	MSE	0.01942896	0.0191772	0.0243568
	R	0.91693522	0.9319823	0.955449
23	MSE	0.01937416	0.01861026	0.02324712
	R	0.91005894	0.92165026	0.95063332
24	MSE	0.01978066	0.01890417	0.0222309
	R	0.90888156	0.94756002	0.93266592
25	MSE	0.01911624	0.018819	0.02340416
	R	0.91234674	0.95003934	0.9499189
26	MSE	0.01914432	0.01888602	0.02284188
	R	0.95112574	0.93276795	0.94062646
27	MSE	0.0194064	0.0187257	0.02365984
	R	0.9580676	0.97187211	0.90349686
28	MSE	0.0188097	0.01830722	0.02440884
	R	0.95186294	0.94305696	0.95845464
29	MSE	0.01883938	0.01891011	0.0236313
	R	0.97207506	0.96371534	0.910672
30	MSE	0.01886731	0.01803258	0.02437995
	R	0.93432405	0.96326944	0.93777466
31	MSE	0.01807799	0.01831095	0.02548954
	R	0.95195024	0.96448072	0.90664692
32	MSE	0.01833588	0.0181896	0.02405788
	R	0.9256744	0.94424736	0.93057696
33	MSE	0.01789316	0.01796118	0.02434571

	R	0.96309402	0.944208	0.9200864
34	MSE	0.01842222	0.01849067	0.02339665
	R	0.91243416	0.93577565	0.89912028
35	MSE	0.01727574	0.01787656	0.0226846
	R	0.95742589	0.97527672	0.94042276
36	MSE	0.01802952	0.01782415	0.02408037
	R	0.97354521	0.96496582	0.9172725
37	MSE	0.01780832	0.01829165	0.02210174
	R	0.95400858	0.92633146	0.91412462
38	MSE	0.01764158	0.01783808	0.02321889
	R	0.96960024	0.92613782	0.93903566
39	MSE	0.01853026	0.01808784	0.02326722
	R	0.97456392	0.93699925	0.91012398
40	MSE	0.0174879	0.01796744	0.02477264
	R	0.96565672	0.97913475	0.9185132
41	MSE	0.01806802	0.0174894	0.0232297
	R	0.97765668	0.97658253	0.92324705
42	MSE	0.01712556	0.01730915	0.02386098
	R	0.96700716	0.91960446	0.92812128
43	MSE	0.01800061	0.01681246	0.02331383
	R	0.96833996	0.97957431	0.91919625
44	MSE	0.01695071	0.0175161	0.02405416
	R	0.9244293	0.97921197	0.89927931
45	MSE	0.01759905	0.01738152	0.02198063
	R	0.9396013	0.93963265	0.91345346

S18. The experimental results for the Smart Home 8, when developing the FITNET ANNs forecasting solution using the total electricity consumption from the grid.

n	The Training Algorithm	The Levenberg-Marquardt Training Algorithm	The Bayesian Regularization Training Algorithm	The Scaled Conjugate Gradient Training Algorithm
1	MSE	0.02861202	0.02759118	0.02949714
	R	0.93143474	0.9129557	0.93142698
2	MSE	0.02738292	0.02734935	0.02906715
	R	0.94186232	0.90346502	0.95075739
3	MSE	0.02586105	0.02715018	0.02899208
	R	0.9134041	0.94249834	0.95070789
4	MSE	0.02634206	0.0251583	0.02581926
	R	0.90461182	0.89517336	0.9238176
5	MSE	0.02590898	0.02564683	0.02788099

	R	0.93431855	0.89611917	0.91303075
6	MSE	0.02526805	0.0242684	0.02654346
	R	0.95489361	0.90820638	0.90354116
7	MSE	0.02461426	0.02331181	0.0255089
	R	0.90860306	0.90010887	0.94417708
8	MSE	0.02325946	0.02305992	0.02632864
	R	0.90081567	0.93249504	0.89452515
9	MSE	0.02354896	0.02353836	0.02440048
	R	0.95146632	0.91301354	0.89729469
10	MSE	0.02241383	0.02160808	0.02557086
	R	0.96212061	0.96365511	0.8961573
11	MSE	0.02256384	0.02230558	0.02640508
	R	0.93308448	0.95362624	0.94212202
12	MSE	0.02253405	0.02261017	0.02377314
	R	0.95287556	0.9536233	0.91804675
13	MSE	0.0215817	0.02088882	0.0243219
	R	0.94453556	0.9250207	0.94457398
14	MSE	0.02132235	0.02179376	0.02391375
	R	0.95450236	0.91546224	0.95866353
15	MSE	0.02033512	0.02073466	0.02337173
	R	0.94538528	0.93555648	0.92984928
16	MSE	0.02054535	0.02026731	0.02501339
	R	0.94507003	0.90604506	0.95629248
17	MSE	0.0192712	0.02029125	0.02254118
	R	0.94966977	0.94575582	0.95116448
18	MSE	0.02017498	0.01994492	0.02458785
	R	0.9268466	0.94543766	0.93637495
19	MSE	0.01950342	0.01955249	0.02346204
	R	0.96527376	0.91733472	0.9325824
20	MSE	0.01955512	0.02009781	0.0257271
	R	0.9373872	0.91802656	0.93404404
21	MSE	0.01975628	0.01972978	0.02430792
	R	0.91960106	0.9200485	0.89848695
22	MSE	0.01980992	0.01881192	0.0238884
	R	0.90718059	0.95160298	0.897543
23	MSE	0.01956045	0.01879452	0.02347065
	R	0.95898684	0.97067421	0.94093298
24	MSE	0.01940744	0.01965285	0.02332065
	R	0.91865448	0.9280227	0.91323538
25	MSE	0.01948386	0.0186345	0.02340416

	R	0.92215692	0.91086246	0.9499189
26	MSE	0.0193284	0.01870785	0.02284188
	R	0.95112574	0.91313073	0.94062646
27	MSE	0.019593	0.01908238	0.0232176
	R	0.928739	0.97187211	0.91321188
28	MSE	0.01863056	0.01795174	0.02440884
	R	0.97148898	0.93323345	0.92941056
29	MSE	0.01830619	0.01837992	0.02408142
	R	0.94261824	0.94404768	0.959112
30	MSE	0.01798566	0.01856295	0.02461214
	R	0.95399403	0.97309872	0.95711022
31	MSE	0.01897294	0.01761339	0.02525132
	R	0.95195024	0.9349558	0.94522764
32	MSE	0.01798992	0.0185394	0.02270884
	R	0.9650648	0.97375509	0.91118994
33	MSE	0.01842464	0.01813727	0.02343559
	R	0.92378406	0.924537	0.94914176
34	MSE	0.01842222	0.01745381	0.0236283
	R	0.91243416	0.91607511	0.95712804
35	MSE	0.01727574	0.01770467	0.0229092
	R	0.94755552	0.95557416	0.95981292
36	MSE	0.01873656	0.0176511	0.02478174
	R	0.97354521	0.94527264	0.926928
37	MSE	0.01886624	0.0174369	0.0225309
	R	0.95400858	0.95589523	0.91412462
38	MSE	0.01747515	0.01732352	0.02436834
	R	0.94989024	0.95569541	0.90999332
39	MSE	0.0181839	0.0179172	0.02417966
	R	0.96471984	0.93699925	0.91980615
40	MSE	0.01765935	0.01729576	0.02338352
	R	0.96565672	0.97913475	0.93785032
41	MSE	0.01739258	0.0173196	0.0221442
	R	0.92828008	0.95685359	0.92324705
42	MSE	0.01814292	0.0171411	0.02455596
	R	0.91767006	0.97893378	0.95712507
43	MSE	0.01715946	0.01697892	0.02354466
	R	0.92881588	0.96960024	0.91919625
44	MSE	0.01776666	0.01684882	0.02359158
	R	0.9641897	0.96932094	0.94762766
45	MSE	0.01662157	0.01788291	0.02263352

R	0.97916346	0.93963265	0.91345346

S19. The comparison of the best experimental results recorded for Smart Homes 1-8 when developing the FITNET ANNs forecasting solution using the total electricity consumption from the grid. The forecasting results of the Smart Home 3, that are presented in detail in the paper, are highlighted in red.

**The Best Forecasting Results**								
**The Smart Home number**	**1**	**2**	**3**	**4**	**5**	**6**	**7**	**8**
**The training algorithm**	BR	BR	LM	LM	BR	BR	BR	LM
**The number of neurons in the hidden layer n**	43	45	44	44	45	44	43	45
**MSE**	0.01681246	0.01697305	0.01695071	0.01662157	0.01688013	0.01695992	0.01681246	0.01662157
**R**	0.97949412	0.97919613	0.97432236	0.97957431	0.97957431	0.97893378	0.97957431	0.97916346

Below are presented the experimental results registered for the Smart Homes 1-8, when developing the artificial neural networks forecasting solution for the total electricity consumption of all the individual appliances, based on the NARX model. In all the tables, the best obtained forecasting results are highlighted in red.

S20. The experimental results for the Smart Home 1, when developing the artificial neural networks forecasting solution for the total electricity consumption of all the individual appliances, based on the NARX model.

**The Levenberg-Marquardt Training Algorithm**					
n	***d***	**8**	**16**	**24**	**48**
6	MSE	0.00586819	0.00632257	0.00641352	0.00634564
	R	0.99229469	0.93192179	0.89721002	0.89193324
12	MSE	0.00599985	0.00641839	0.00616214	0.00611020
	R	0.99909901	0.94793328	0.97143596	0.98829705
24	MSE	0.00619839	0.00593968	0.00581091	0.00597474
	R	0.99906716	0.99707306	0.9992905	0.99908051
**The Bayesian Regularization Training Algorithm**					
n	***d***	**8**	**16**	**24**	**48**
6	MSE	0.00611032	0.00600871	0.00576897	0.00575061
	R	0.96822953	0.97665334	0.98480786	0.99901967
12	MSE	0.00589833	0.0059997	0.00560643	0.00542371
	R	0.99920265	0.9976615	0.99909697	0.99906748
24	MSE	0.00576954	0.00596158	0.0055959	0.00498614
	R	0.99363678	0.99099293	0.99643307	0.99853660
**The Scaled Conjugate Gradient Training Algorithm**					
n	***d***	**8**	**16**	**24**	**48**
6	MSE	0.00571466	0.00598589	0.0062524	0.00639407
	R	0.99930172	0.98790575	0.99901011	0.98441710
12	MSE	0.00694051	0.00621034	0.00661947	0.00627552

	R	0.93943706	0.98384291	0.96256672	0.99900120
24	MSE	0.00638039	0.00658446	0.0068212	0.00617734
	R	0.95705274	0.97192389	0.93989853	0.96532214

S21. The experimental results for the Smart Home 2, when developing the artificial neural networks forecasting solution for the total electricity consumption of all the individual appliances, based on the NARX model.

**The Levenberg-Marquardt Training Algorithm**					
n	***d***	**8**	**16**	**24**	**48**
6	MSE	0.00617383	0.00677418	0.00654308	0.006869
	R	0.91522326	0.92241238	0.90665434	0.90132202
12	MSE	0.00624475	0.00694775	0.00667566	0.00642195
	R	0.9521921	0.90100589	0.94286432	0.94991658
24	MSE	0.00651144	0.00624273	0.00622598	0.00609545
	R	0.93960117	0.9196305	0.95064617	0.92081648
**The Bayesian Regularization Training Algorithm**					
n	***d***	**8**	**16**	**24**	**48**
6	MSE	0.00655127	0.00631218	0.00619405	0.00556679
	R	0.94905667	0.92830416	0.93653296	0.97249789
12	MSE	0.00613907	0.00624211	0.00613756	0.0057062
	R	0.95224709	0.94923133	0.97048581	0.95739757
24	MSE	0.00637686	0.00626267	0.0058243	0.00534972
	R	0.92610806	0.96184608	0.95697038	0.9491041
**The Scaled Conjugate Gradient Training Algorithm**					
n	***d***	**8**	**16**	**24**	**48**
6	MSE	0.00595031	0.00629765	0.0065076	0.00706713
	R	0.97130494	0.92076653	0.92474368	0.9281647
12	MSE	0.00759119	0.00653379	0.00716541	0.00672378
	R	0.91153299	0.90816269	0.90649488	0.91439616
24	MSE	0.00698485	0.00705962	0.006959	0.00662314
	R	0.89137265	0.89716051	0.92128667	0.91753392

S22. The experimental results for the Smart Home 3, when developing the artificial neural networks forecasting solution for the total electricity consumption of all the individual appliances, based on the NARX model.

**The Levenberg-Marquardt Training Algorithm**					
n	***d***	**8**	**16**	**24**	**48**
6	MSE	0.006113	0.006452	0.006478	0.006542
	R	0.963393	0.950941	0.944432	0.938877

12	MSE	0.006122	0.006617	0.006419	0.006235
	R	0.96181	0.938548	0.952388	0.959512
24	MSE	0.006261	0.006061	0.00593	0.006035
	R	0.958777	0.968032	0.980048	0.969281
**The Bayesian Regularization Training Algorithm**					
n	***d***	**8**	**16**	**24**	**48**
6	MSE	0.006299	0.006069	0.006073	0.005809
	R	0.958643	0.966984	0.965498	0.982321
12	MSE	0.006019	0.00606	0.005902	0.00565
	R	0.971681	0.968603	0.980289	0.987008
24	MSE	0.006073	0.006022	0.00571	0.005194
	R	0.964696	0.971562	0.986567	0.98865
**The Scaled Conjugate Gradient Training Algorithm**					
n	***d***	**8**	**16**	**24**	**48**
6	MSE	0.005891	0.006235	0.00638	0.006731
	R	0.981116	0.959132	0.953344	0.93754
12	MSE	0.00723	0.006469	0.006824	0.006404
	R	0.930136	0.946003	0.934531	0.952496
24	MSE	0.006716	0.006788	0.00689	0.006368
	R	0.938287	0.934542	0.930593	0.955765

S23. The experimental results for the Smart Home 4, when developing the artificial neural networks forecasting solution for the total electricity consumption of all the individual appliances, based on the NARX model.

**The Levenberg-Marquardt Training Algorithm**					
n	***d***	**8**	**16**	**24**	**48**
6	MSE	0.00580707	0.00632257	0.00615439	0.00647648
	R	0.99115625	0.92241238	0.90665434	0.90132202
12	MSE	0.00599985	0.00641839	0.00609796	0.00592316
	R	0.9810464	0.94793328	0.96191208	0.98829705
24	MSE	0.00607317	0.00593968	0.00575162	0.00503808
	R	0.96836447	0.97771242	0.98984808	0.99698882
**The Bayesian Regularization Training Algorithm**					
n	***d***	**8**	**16**	**24**	**48**
6	MSE	0.0059144	0.00594801	0.00595115	0.00563444
	R	0.99080517	0.99056628	0.99446284	0.99314372
12	MSE	0.00589833	0.00593909	0.00566544	0.00548021
	R	0.99008311	0.97828943	0.99096974	0.99363582
24	MSE	0.00601247	0.00584115	0.0055959	0.00611032

	R	0.99363678	0.99201398	0.99643307	0.99084231
**The Scaled Conjugate Gradient Training Algorithm**					
n	***d***	**8**	**16**	**24**	**48**
6	MSE	0.00565574	0.00604824	0.0062524	0.00666329
	R	0.99007384	0.97831444	0.99147776	0.9469155
12	MSE	0.0071574	0.00614565	0.00648299	0.00621149
	R	0.96734113	0.97438288	0.98125734	0.96202096
24	MSE	0.00658188	0.00651658	0.0068212	0.00630472
	R	0.95705274	0.94388762	0.93989853	0.97487979

S24. The experimental results for the Smart Home 5, when developing the artificial neural networks forecasting solution for the total electricity consumption of all the individual appliances, based on the NARX model.

**The Levenberg-Marquardt Training Algorithm**					
n	***d***	**8**	**16**	**24**	**48**
6	MSE	0.00623495	0.00677418	0.00667265	0.00680358
	R	0.92485718	0.93192179	0.90665434	0.91071079
12	MSE	0.00630597	0.00694775	0.00673985	0.0063596
	R	0.942574	0.90100589	0.92381655	0.92113123
24	MSE	0.00644883	0.00636395	0.0059888	0.00609545
	R	0.94918893	0.92931082	0.96044665	0.94989489
**The Bayesian Regularization Training Algorithm**					
n	***d***	**8**	**16**	**24**	**48**
6	MSE	0.00636229	0.00625148	0.0063155	0.00604105
	R	0.92988381	0.937974	0.95584292	0.93320505
12	MSE	0.00625945	0.0061209	0.00613756	0.0057062
	R	0.92309667	0.9395453	0.96068293	0.93765741
24	MSE	0.00619466	0.00614224	0.0059385	0.00529778
	R	0.93575502	0.94241485	0.93723903	0.9688771
**The Scaled Conjugate Gradient Training Algorithm**					
n	***d***	**8**	**16**	**24**	**48**
6	MSE	0.00618597	0.00629765	0.0065076	0.00686521
	R	0.9320603	0.91117521	0.91521024	0.9000385
12	MSE	0.00751889	0.00659848	0.00696068	0.00653167
	R	0.88362892	0.91762272	0.90649488	0.91439616
24	MSE	0.00698485	0.00685598	0.0070968	0.00668682
	R	0.91952126	0.89716051	0.8933689	0.94620686

S25. The experimental results for the Smart Home 6, when developing the artificial neural networks forecasting solution for the total electricity consumption of all the individual appliances, based on the NARX model.

**The Levenberg-Marquardt Training Algorithm**					
n	***d***	**8**	**16**	**24**	**48**
6	MSE	0.00605157	0.00638708	0.00628395	0.00634564
	R	0.97302683	0.93192179	0.93498728	0.91071079
12	MSE	0.00587741	0.00641839	0.00609796	0.00604785
	R	0.99066451	0.95731876	0.96191208	0.97870193
24	MSE	0.00619839	0.00600029	0.00575162	0.00585405
	R	0.98754	0.97771242	0.99909449	0.99835892
**The Bayesian Regularization Training Algorithm**					
n	***d***	**8**	**16**	**24**	**48**
6	MSE	0.00623631	0.00588732	0.00576897	0.00563444
	R	0.97781596	0.98632317	0.97515288	0.99901968
12	MSE	0.00595851	0.00575729	0.00560643	0.00536722
	R	0.99900831	0.97828943	0.999195	0.99687788
24	MSE	0.005891	0.00572071	0.005053	0.00503808
	R	0.99912931	0.99099293	0.99935896	0.99908423
**The Scaled Conjugate Gradient Training Algorithm**					
n	***d***	**8**	**16**	**24**	**48**
6	MSE	0.00577357	0.00592354	0.0062524	0.00666329
	R	0.99920361	0.99749707	0.99147776	0.9750417
12	MSE	0.00701281	0.00640441	0.00648299	0.00614746
	R	0.96734113	0.99330294	0.96256672	0.99059584
24	MSE	0.00658188	0.00672022	0.0068212	0.00624103
	R	0.95705274	0.97192389	0.95851038	0.98443744

S26. The experimental results for the Smart Home 7, when developing the artificial neural networks forecasting solution for the total electricity consumption of all the individual appliances, based on the NARX model.

**The Levenberg-Marquardt Training Algorithm**					
n	***d***	**8**	**16**	**24**	**48**
6	MSE	0.00635721	0.00677418	0.00654308	0.00667274
	R	0.93449111	0.93192178	0.90665434	0.89193325
12	MSE	0.00636719	0.00674923	0.00667566	0.00629725
	R	0.93295589	0.92916232	0.93334044	0.92113123
24	MSE	0.00644883	0.00630333	0.00610739	0.00540166
	R	0.9300134	0.94867145	0.9408457	0.9787636

**The Bayesian Regularization Training Algorithm**					
n	***d***	**8**	**16**	**24**	**48**
6	MSE	0.00642529	0.00631217	0.00613333	0.00592487
	R	0.94905667	0.94764383	0.95584292	0.93320505
12	MSE	0.00619926	0.00624210	0.00613756	0.00581919
	R	0.96196389	0.94923133	0.93127427	0.95739757
24	MSE	0.00613393	0.00614223	0.0058814	0.00609545
	R	0.93575502	0.96184608	0.97670173	0.92081648
**The Scaled Conjugate Gradient Training Algorithm**					
n	***d***	**8**	**16**	**24**	**48**
6	MSE	0.00606814	0.00629765	0.0066352	0.00693252
	R	0.95168262	0.92076652	0.91521024	0.9281647
12	MSE	0.00737429	0.00672786	0.00716541	0.00665974
	R	0.91153299	0.89870266	0.91584018	0.91439616
24	MSE	0.00685052	0.00699174	0.0070279	0.00668682
	R	0.90075552	0.91585135	0.91198075	0.91753392

S27. The experimental results for the Smart Home 8, when developing the artificial neural networks forecasting solution for the total electricity consumption of all the individual appliances, based on the NARX model.

**The Levenberg-Marquardt Training Algorithm**					
n	***d***	**8**	**16**	**24**	**48**
6	MSE	0.00599045	0.00612902	0.00615439	0.00621481
	R	0.98266076	0.93192179	0.92554297	0.91071079
12	MSE	0.00606108	0.00628606	0.00609796	0.00604785
	R	0.99909901	0.94793328	0.96191208	0.98829705
24	MSE	0.00607317	0.00581846	0.00575162	0.00573335
	R	0.99906716	0.98739274	0.99909449	0.97897331
**The Bayesian Regularization Training Algorithm**					
n	***d***	**8**	**16**	**24**	**48**
6	MSE	0.00617331	0.00594801	0.00589042	0.00557635
	R	0.96822953	0.99599301	0.97515288	0.99921614
12	MSE	0.00595851	0.00593909	0.00566544	0.00536722
	R	0.99920265	0.98797547	0.99989447	0.99926488
24	MSE	0.00601247	0.00590136	0.0055388	0.00509002
	R	0.99903284	0.99900709	0.99935896	0.9991831
**The Scaled Conjugate Gradient Training Algorithm**					
n	***d***	**8**	**16**	**24**	**48**
6	MSE	0.00583249	0.00598589	0.006061	0.00639407

	R	0.9991055	0.96872312	0.98194432	0.98441711
12	MSE	0.00686822	0.00614565	0.00668772	0.00627553
	R	0.95803977	0.98384291	0.97191203	0.99900121
24	MSE	0.00664904	0.00665234	0.0065456	0.00624103
	R	0.95705274	0.97192389	0.95851038	0.97487979

S28. The comparison of the best experimental results recorded for Smart Homes 1-8 when developing the artificial neural networks forecasting solution for the total electricity consumption of all the individual appliances, based on the NARX model. The forecasting results of the Smart Home 3, that are presented in detail in the paper, are highlighted in red.

	**The Best Forecasting Results**							
**The Smart Home number**	**1**	**2**	**3**	**4**	**5**	**6**	**7**	**8**
**The training algorithm**	BR	BR	BR	LM	BR	BR	LM	BR
**The number of neurons in the hidden layer n**	24	6	24	24	24	24	24	24
**The delay parameter d**	48	48	48	48	48	24	48	48
**MSE**	0.00498614	0.00556679	0.005194	0.00503808	0.00529778	0.005053	0.00540166	0.00509002
**R**	0.99853660	0.97249789	0.98865	0.99698882	0.9688771	0.99935896	0.9787636	0.9991831

Below are presented the experimental results registered for the Smart Homes 1-8**,** when developing the FITNET ANNs forecasting solution using the total electricity consumption of all the individual appliances. In all the tables, the best obtained forecasting results are highlighted in red.

S29. The experimental results for the Smart Home 1, when developing the FITNET ANNs forecasting solution using the total electricity consumption of all the individual appliances.

n	**The Training Algorithm**	**The Levenberg-Marquardt Training Algorithm**	**The Bayesian Regularization Training Algorithm**	**The Scaled Conjugate Gradient Training Algorithm**
1	MSE	0.02777049	0.02649846	0.02749248
	R	0.99865168	0.99090563	0.97943868
2	MSE	0.0255037	0.02526559	0.02685251
	R	0.99091382	0.98996699	0.99877544
3	MSE	0.02534895	0.02486652	0.02759823
	R	0.98070756	0.990981965	0.99872344
4	MSE	0.02460249	0.0241717	0.02480674
	R	0.97197653	0.990105408	0.9815562
5	MSE	0.02372972	0.02372931	0.02501472
	R	0.9824793	0.963569	0.961085
6	MSE	0.02337885	0.0228683	0.02524231
	R	0.99031206	0.98550054	0.90966256

7	MSE	0.02229216	0.02238857	0.0235837
	R	0.99559697	0.98721618	0.98271492
8	MSE	0.0214529	0.02128608	0.02480968
	R	0.991705	0.990048947	0.99003292
9	MSE	0.02132736	0.02131776	0.02252352
	R	0.99194282	0.991014264	0.99034263
10	MSE	0.02110817	0.02036146	0.02318106
	R	0.99127578	0.98312289	0.9732461
11	MSE	0.02147904	0.02083257	0.0243542
	R	0.98168263	0.99254976	0.99094165
12	MSE	0.02103178	0.02070838	0.02260779
	R	0.99014917	0.99120084	0.99050196
13	MSE	0.02014292	0.02047518	0.02312965
	R	0.99322296	0.990291718	0.99276653
14	MSE	0.01949472	0.01975696	0.02209175
	R	0.99226811	0.990311288	0.98771394
15	MSE	0.01857535	0.01916978	0.02201027
	R	0.9913609	0.99402876	0.99765079
16	MSE	0.01858865	0.01928346	0.02267569
	R	0.9913271	0.99372684	0.99493056
17	MSE	0.0179741	0.018552	0.02142528
	R	0.99862182	0.99237563	0.9909399
18	MSE	0.01846201	0.0183958	0.02248032
	R	0.99244094	0.990391834	0.99429505
19	MSE	0.01854737	0.01822368	0.0210273
	R	0.99237752	0.99540576	0.99102976
20	MSE	0.01786285	0.01859517	0.02425698
	R	0.9959739	0.990592272	0.98219064
21	MSE	0.01845162	0.01842687	0.02267181
	R	0.9907648	0.992771375	0.99144208
22	MSE	0.01885752	0.01771608	0.0229516
	R	0.99242362	0.990065468	0.984402
23	MSE	0.01807013	0.01768896	0.02212947
	R	0.99274859	0.991969816	0.99088354
24	MSE	0.01847439	0.01852983	0.02114115
	R	0.99261566	0.99257093	0.99006728
25	MSE	0.01782957	0.017712	0.02182888
	R	0.99300689	0.98921622	0.99838415
26	MSE	0.01822392	0.01763883	0.02217006

	R	0.99034742	0.993095405	0.99182039
27	MSE	0.0179136	0.01729898	0.02144864
	R	0.9971724	0.99150589	0.99093204
28	MSE	0.01755572	0.01759626	0.02189952
	R	0.99303671	0.992164504	0.99068614
29	MSE	0.01759527	0.01714281	0.0213807
	R	0.99015319	0.992271832	0.991724
30	MSE	0.01710401	0.01697184	0.02298681
	R	0.99333399	0.99275728	0.97644578
31	MSE	0.01700405	0.01691583	0.02334556
	R	0.99108338	0.984164	0.964518
32	MSE	0.01660608	0.0167904	0.02180948
	R	0.9933998	0.99342691	0.98873802
33	MSE	0.01718452	0.01672855	0.02229794
	R	0.99122315	0.99327275	0.97819712
34	MSE	0.01788039	0.01676257	0.02247005
	R	0.99105454	0.990472754	0.99579988
35	MSE	0.01676763	0.01667333	0.0215616
	R	0.99690737	0.991468184	0.97920308
36	MSE	0.01732248	0.01678585	0.02244384
	R	0.99321279	0.992404536	0.9752055
37	MSE	0.0167504	0.01692405	0.02124342
	R	0.99334914	0.992487736	0.98219773
38	MSE	0.01647657	0.01646592	0.02229933
	R	0.99036369	0.990495806	0.99068011
39	MSE	0.01662528	0.01689336	0.02167045
	R	0.99139402	0.991590445	0.98758134
40	MSE	0.0168021	0.01628824	0.02268896
	R	0.99521764	0.993847625	0.99055302
41	MSE	0.01621056	0.0168102	0.0212758
	R	0.99072826	0.992590488	0.99127578
42	MSE	0.0161082	0.0161328	0.02270268
	R	0.99360791	0.990859844	0.97646093
43	MSE	0.01598185	0.01614662	0.02262134
	R	0.99175071	0.99388574	0.9869265
44	MSE	0.01612786	0.0158479	0.02220384
	R	0.99138902	0.990888506	0.99597601
45	MSE	0.01609056	0.01547735	0.02089248
	R	0.99286162	0.99385414	0.99009118

S30. The experimental results for the Smart Home 2, when developing the FITNET ANNs forecasting solution using the total electricity consumption of all the individual appliances.

n	The Training Algorithm	The Levenberg-Marquardt Training Algorithm	The Bayesian Regularization Training Algorithm	The Scaled Conjugate Gradient Training Algorithm
1	MSE	0.02833151	0.02841072	0.02921076
	R	0.95063958	0.93217582	0.9122223
2	MSE	0.02738292	0.02682841	0.02906715
	R	0.93225148	0.95152167	0.93155017
3	MSE	0.02637315	0.02638896	0.02815577
	R	0.92301888	0.91364635	0.91229545
4	MSE	0.02609355	0.02491165	0.02556613
	R	0.95272947	0.95292648	0.9334407
5	MSE	0.02469828	0.02468807	0.02631757
	R	0.9439507	0.95393331	0.9226416
6	MSE	0.02479575	0.0238017	0.02628323
	R	0.92595744	0.91786815	0.9131533
7	MSE	0.02438205	0.02400424	0.02430565
	R	0.95693301	0.91946605	0.9152737
8	MSE	0.02280782	0.02283819	0.02556916
	R	0.92987424	0.95192202	0.9426179
9	MSE	0.02310464	0.02287218	0.02416586
	R	0.9223398	0.94215227	0.92623968
10	MSE	0.02284905	0.02140031	0.0250929
	R	0.95240222	0.94418733	0.9154295
11	MSE	0.02212992	0.02167429	0.0269178
	R	0.92336485	0.96335712	0.95173551
12	MSE	0.02167561	0.02218755	0.02354007
	R	0.94315234	0.9341616	0.9277104
13	MSE	0.02075954	0.02088882	0.02408345
	R	0.93479808	0.93475776	0.91565845
14	MSE	0.02111928	0.02118272	0.02391375
	R	0.9252829	0.94467912	0.91992965
15	MSE	0.02053065	0.02014783	0.02291791
	R	0.93563904	0.95504724	0.93953521
16	MSE	0.02015401	0.02007054	0.02361077
	R	0.95481302	0.93527232	0.95629248
17	MSE	0.0190859	0.01951825	0.02254118
	R	0.93987936	0.94575582	0.93175296

18	MSE	0.01922333	0.01955764	0.02365117
	R	0.93660288	0.9259441	0.9267216
19	MSE	0.02007705	0.01917283	0.02257668
	R	0.95552352	0.94661136	0.922868
20	MSE	0.01917906	0.01953432	0.02474702
	R	0.9373872	0.93755904	0.95330268
21	MSE	0.01938352	0.01879913	0.02384046
	R	0.96851601	0.9591995	0.9274704
22	MSE	0.01923848	0.01844664	0.0243568
	R	0.95595374	0.9319823	0.955449
23	MSE	0.01900158	0.01879452	0.02347065
	R	0.96877242	0.96086942	0.95063332
24	MSE	0.01940744	0.01909134	0.0222309
	R	0.95774616	0.95732868	0.92295065
25	MSE	0.01856481	0.0190035	0.02340416
	R	0.97120782	0.9304509	0.9305328
26	MSE	0.01877616	0.01799517	0.0235137
	R	0.96093116	0.95240517	0.95032364
27	MSE	0.0188466	0.01801234	0.02255424
	R	0.9482914	0.94242144	0.93264192
28	MSE	0.0188097	0.01848496	0.02326824
	R	0.9322369	0.94305696	0.92941056
29	MSE	0.01866165	0.01837992	0.02273106
	R	0.9327993	0.93421385	0.930048
30	MSE	0.01780933	0.01856295	0.02414776
	R	0.95399403	0.94361088	0.95711022
31	MSE	0.01825698	0.01761339	0.02406022
	R	0.9323224	0.94479744	0.95487282
32	MSE	0.01764396	0.0181896	0.0236082
	R	0.935522	0.93441145	0.95965749
33	MSE	0.01789316	0.01813727	0.02298053
	R	0.97292151	0.9737145	0.92977152
34	MSE	0.01860283	0.01762662	0.02385995
	R	0.97130088	0.94562592	0.93779212
35	MSE	0.01778385	0.01736089	0.023583
	R	0.94755552	0.96542544	0.9210326
36	MSE	0.01802952	0.0179972	0.02384658
	R	0.93421005	0.94527264	0.9558945
37	MSE	0.0185136	0.0174369	0.02167258

	R	0.9343383	0.93618605	0.94329881
38	MSE	0.0178308	0.0180096	0.02390856
	R	0.95978493	0.97540047	0.94871644
39	MSE	0.0181839	0.0179172	0.02326722
	R	0.97456392	0.93699925	0.93917049
40	MSE	0.0168094	0.01712784	0.02361504
	R	0.9755104	0.93957375	0.94751888
41	MSE	0.01756144	0.0171498	0.0227955
	R	0.94803072	0.96671806	0.92324705
42	MSE	0.01746468	0.0174772	0.02339766
	R	0.96700716	0.9393809	0.92812128
43	MSE	0.01749592	0.01731184	0.02423715
	R	0.95845894	0.95970636	0.93854775
44	MSE	0.01711528	0.01684882	0.02382287
	R	0.9741298	0.96932094	0.92828832
45	MSE	0.01743144	0.01738152	0.02263352
	R	0.95938238	0.95941439	0.92317105

S31. The experimental results for the Smart Home 3, when developing the FITNET ANNs forecasting solution using the total electricity consumption of all the individual appliances.

n	The Training Algorithm	The Levenberg-Marquardt Training Algorithm	The Bayesian Regularization Training Algorithm	The Scaled Conjugate Gradient Training Algorithm
1	MSE	0.028051	0.027318	0.028638
	R	0.960242	0.961006	0.960234
2	MSE	0.026846	0.026047	0.027683
	R	0.961084	0.961133	0.960361
3	MSE	0.025605	0.025374	0.027877
	R	0.961478	0.961733	0.960311
4	MSE	0.024851	0.024665	0.025313
	R	0.962353	0.962552	0.962310
5	MSE	0.024214	0.023969	0.026057
	R	0.963215	0.963569	0.961085
6	MSE	0.023615	0.023335	0.026023
	R	0.964539	0.966177	0.961214
7	MSE	0.023221	0.023081	0.024065
	R	0.966599	0.967859	0.963446
8	MSE	0.022582	0.022173	0.025316

	R	0.968619	0.971349	0.961855
9	MSE	0.022216	0.022206	0.023462
	R	0.970884	0.971291	0.964833
10	MSE	0.021761	0.020777	0.023898
	R	0.971839	0.973389	0.963610
11	MSE	0.021696	0.021043	0.025636
	R	0.971963	0.973088	0.961349
12	MSE	0.021461	0.021131	0.023307
	R	0.972322	0.973085	0.966365
13	MSE	0.020554	0.020682	0.023845
	R	0.973748	0.973706	0.963851
14	MSE	0.020307	0.020368	0.022775
	R	0.973982	0.973896	0.968347
15	MSE	0.019553	0.019561	0.022691
	R	0.974624	0.974538	0.968593
16	MSE	0.019567	0.019677	0.023377
	R	0.974299	0.974242	0.965952
17	MSE	0.018530	0.019325	0.022318
	R	0.979041	0.975006	0.970576
18	MSE	0.019033	0.019364	0.023417
	R	0.975628	0.974678	0.965335
19	MSE	0.019121	0.018983	0.022134
	R	0.975024	0.975888	0.971440
20	MSE	0.018803	0.018783	0.024502
	R	0.976445	0.976624	0.962932
21	MSE	0.018638	0.018613	0.023373
	R	0.978299	0.978775	0.966115
22	MSE	0.019048	0.018264	0.023420
	R	0.975463	0.981034	0.965100
23	MSE	0.018629	0.018426	0.022353
	R	0.978558	0.980479	0.970034
24	MSE	0.018661	0.018717	0.021795
	R	0.977292	0.976866	0.971527
25	MSE	0.018381	0.01845	0.022504
	R	0.981018	0.979422	0.969305
26	MSE	0.018408	0.017817	0.022394
	R	0.980542	0.981861	0.969718
27	MSE	0.018660	0.017834	0.022112
	R	0.977620	0.981689	0.971502
28	MSE	0.017914	0.017774	0.022812

	R	0.981302	0.982351	0.968136
29	MSE	0.017773	0.017673	0.022506
	R	0.981894	0.983383	0.968800
30	MSE	0.017633	0.017679	0.023219
	R	0.983499	0.982928	0.966778
31	MSE	0.017899	0.017439	0.023822
	R	0.981392	0.984164	0.964518
32	MSE	0.017298	0.017490	0.022484
	R	0.984760	0.983591	0.969351
33	MSE	0.017716	0.017609	0.022753
	R	0.982749	0.983550	0.968512
34	MSE	0.018061	0.017281	0.023165
	R	0.981112	0.985027	0.966796
35	MSE	0.016937	0.017189	0.022460
	R	0.987037	0.985128	0.969508
36	MSE	0.017676	0.017305	0.023379
	R	0.983379	0.984659	0.965550
37	MSE	0.017632	0.017095	0.021458
	R	0.983514	0.985459	0.972473
38	MSE	0.016643	0.017152	0.022989
	R	0.989469	0.985253	0.968078
39	MSE	0.017318	0.017064	0.022811
	R	0.984408	0.986315	0.968217
40	MSE	0.017145	0.016792	0.023152
	R	0.985364	0.989025	0.966856
41	MSE	0.016886	0.016980	0.021710
	R	0.987532	0.986447	0.971839
42	MSE	0.016956	0.016805	0.023166
	R	0.986742	0.988822	0.966793
43	MSE	0.016823	0.016646	0.023083
	R	0.988102	0.989388	0.967575
44	MSE	0.016457	0.016682	0.023129
	R	0.994010	0.989103	0.966967
45	MSE	0.016761	0.016713	0.021763
	R	0.989054	0.989087	0.971759

S32. The experimental results for the Smart Home 4, when developing the FITNET ANNs forecasting solution using the total electricity consumption of all the individual appliances.

n	The Training Algorithm	The Levenberg-Marquardt Training Algorithm	The Bayesian Regularization Training Algorithm	The Scaled Conjugate Gradient Training Algorithm
1	MSE	0.02692896	0.02649846	0.0272061
	R	0.99865168	0.99090563	0.96983634
2	MSE	0.02630908	0.02474465	0.02629885
	R	0.99052736	0.97074433	0.96996461
3	MSE	0.0250929	0.02435904	0.02731946
	R	0.97109278	0.990020232	0.99083266
4	MSE	0.02435398	0.02441835	0.02404735
	R	0.99122359	0.990105408	0.99104255
5	MSE	0.02372972	0.02324993	0.02475415
	R	0.99113758	0.963569	0.961085
6	MSE	0.02243425	0.0228683	0.02472185
	R	0.99347517	0.990482408	0.99092747
7	MSE	0.02298879	0.02192695	0.02334305
	R	0.98593098	0.99689477	0.99019838
8	MSE	0.02213036	0.02150781	0.02506284
	R	0.99073638	0.991991645	0.99071065
9	MSE	0.02154952	0.02176188	0.02322738
	R	0.99000105	0.991014264	0.99034263
10	MSE	0.02110817	0.02056923	0.0227031
	R	0.98155739	0.99285678	0.9732461
11	MSE	0.02126208	0.01999085	0.02537964
	R	0.99108415	0.98281888	0.99018947
12	MSE	0.02060256	0.02070838	0.02260779
	R	0.98204522	0.992173925	0.99535595
13	MSE	0.02014292	0.01985472	0.02312965
	R	0.99224354	0.991265424	0.9902405
14	MSE	0.01969779	0.01955328	0.021864
	R	0.98372182	0.99225908	0.99070809
15	MSE	0.01857535	0.01897417	0.02155645
	R	0.99411648	0.99402876	0.97827893
16	MSE	0.01937133	0.01948023	0.02314323
	R	0.9923014	0.99229541	0.99493056
17	MSE	0.0176035	0.01913175	0.02187164
	R	0.99182026	0.98475606	0.99191048

18	MSE	0.01846201	0.01858944	0.02294866
	R	0.98538428	0.990391834	0.99136018
19	MSE	0.01873858	0.01822368	0.0210273
	R	0.98477424	0.99540576	0.9920012
20	MSE	0.01823891	0.01840734	0.02376694
	R	0.99155028	0.991568896	0.99110786
21	MSE	0.01789248	0.01824074	0.02290554
	R	0.9917431	0.9983505	0.99144208
22	MSE	0.01885752	0.01753344	0.022249
	R	0.98521763	0.99084434	0.9913355
23	MSE	0.01788384	0.0175047	0.02168241
	R	0.98834358	0.990008858	0.99088354
24	MSE	0.01772795	0.01852983	0.0209232
	R	0.99163837	0.990617198	0.99095754
25	MSE	0.01746195	0.0182655	0.02205392
	R	0.99082818	0.90901044	0.97899805
26	MSE	0.01785576	0.01728249	0.02217006
	R	0.99034742	0.993095405	0.99182039
27	MSE	0.0181002	0.01765566	0.02122752
	R	0.9926501	0.991113967	0.99006471
28	MSE	0.01755572	0.01706304	0.02235576
	R	0.99111502	0.991182153	0.99068614
29	MSE	0.01706208	0.01749627	0.02228094
	R	0.99015319	0.990305066	0.9907552
30	MSE	0.01692768	0.01714863	0.02275462
	R	0.9913004	0.990258656	0.99578134
31	MSE	0.01754102	0.01656705	0.02310734
	R	0.99120592	0.984164	0.964518
32	MSE	0.01695204	0.0167904	0.02158464
	R	0.99143028	0.990326282	0.9908125
33	MSE	0.01700736	0.01725682	0.02161535
	R	0.99318865	0.99130565	0.99072525
34	MSE	0.01769978	0.01676257	0.02293335
	R	0.99092312	0.990472754	0.99054678
35	MSE	0.01642889	0.01667333	0.021337
	R	0.99067777	0.990483056	0.99859324
36	MSE	0.01714572	0.0169589	0.02267763
	R	0.99128804	0.991419877	0.9904172
37	MSE	0.0167504	0.0164112	0.02124342

	R	0.99031843	0.992487736	0.99016472
38	MSE	0.01614371	0.0162944	0.02275911
	R	0.99092584	0.992466312	0.99712034
39	MSE	0.01714482	0.01655208	0.02167045
	R	0.99425208	0.993563075	0.97789917
40	MSE	0.01628775	0.01628824	0.02292048
	R	0.99521764	0.99891525	0.99055302
41	MSE	0.0160417	0.0166404	0.0210587
	R	0.99369086	0.99631147	0.99009942
42	MSE	0.01627776	0.01663695	0.02293434
	R	0.99262117	0.992837488	0.97646093
43	MSE	0.01665477	0.01614662	0.02192885
	R	0.9907864	0.99028188	0.99159538
44	MSE	0.01629243	0.01634836	0.02197255
	R	0.99238303	0.993855815	0.99056457
45	MSE	0.01642578	0.01587735	0.02067485
	R	0.99187256	0.99586505	0.99106294

S33. The experimental results for the Smart Home 5, when developing the FITNET ANNs forecasting solution using the total electricity consumption of all the individual appliances.

n	The Training Algorithm	The Levenberg-Marquardt Training Algorithm	The Bayesian Regularization Training Algorithm	The Scaled Conjugate Gradient Training Algorithm
1	MSE	0.02833151	0.02759118	0.02949714
	R	0.9122299	0.92256576	0.95063166
2	MSE	0.02738292	0.02682841	0.02906715
	R	0.92264064	0.91307635	0.95075739
3	MSE	0.0266292	0.02638896	0.02815577
	R	0.93263366	0.92326368	0.91229545
4	MSE	0.02609355	0.0256516	0.02556613
	R	0.93348241	0.93367544	0.9334407
5	MSE	0.02445614	0.02516745	0.02657814
	R	0.91505425	0.94429762	0.9226416
6	MSE	0.0240873	0.0238017	0.02654346
	R	0.94524822	0.92752992	0.95160186
7	MSE	0.02438205	0.02377343	0.02478695
	R	0.92793504	0.94850182	0.93454262
8	MSE	0.02348528	0.02305992	0.02632864

	R	0.94924662	0.95192202	0.93299935
9	MSE	0.02288248	0.02309424	0.02393124
	R	0.96117516	0.96157809	0.92623968
10	MSE	0.02263144	0.02119254	0.02437596
	R	0.93296544	0.93445344	0.9443378
11	MSE	0.02234688	0.02125343	0.02640508
	R	0.93308448	0.95362624	0.94212202
12	MSE	0.02231944	0.02134231	0.02354007
	R	0.93342912	0.9536233	0.91804675
13	MSE	0.02096508	0.02150928	0.02503725
	R	0.9250606	0.93475776	0.92529696
14	MSE	0.02051007	0.0213864	0.02391375
	R	0.93502272	0.96415704	0.91992965
15	MSE	0.02013959	0.02014783	0.02314482
	R	0.93563904	0.94530186	0.92016335
16	MSE	0.02015401	0.01987377	0.02384454
	R	0.95481302	0.93527232	0.95629248
17	MSE	0.0194565	0.02029125	0.0234339
	R	0.93987936	0.94575582	0.9220472
18	MSE	0.01922333	0.01994492	0.02458785
	R	0.95611544	0.93569088	0.9460283
19	MSE	0.01931221	0.01974232	0.02257668
	R	0.96527376	0.9270936	0.9325824
20	MSE	0.01899103	0.01972215	0.02499204
	R	0.9373872	0.95709152	0.92441472
21	MSE	0.0195699	0.01935752	0.02384046
	R	0.93916704	0.96898725	0.91780925
22	MSE	0.01980992	0.01881192	0.0238884
	R	0.96570837	0.96141332	0.955449
23	MSE	0.01881529	0.01916304	0.02257653
	R	0.96877242	0.93145505	0.9215323
24	MSE	0.01884761	0.01909134	0.02288475
	R	0.93820032	0.9280227	0.93266592
25	MSE	0.01856481	0.019188	0.02295408
	R	0.9319671	0.96962778	0.95961195
26	MSE	0.01859208	0.01817334	0.02261794
	R	0.97073658	0.93276795	0.95032364
27	MSE	0.0190332	0.01801234	0.0232176
	R	0.9580676	0.97187211	0.96178698
28	MSE	0.0188097	0.01795174	0.02304012

	R	0.97148898	0.97252749	0.95845464
29	MSE	0.01812846	0.01802646	0.02273106
	R	0.9327993	0.96371534	0.959112
30	MSE	0.01816199	0.01785579	0.02345119
	R	0.94415904	0.96326944	0.93777466
31	MSE	0.01879395	0.01813656	0.02406022
	R	0.96176416	0.9349558	0.94522764
32	MSE	0.01747098	0.0180147	0.02293368
	R	0.9552172	0.96391918	0.95965749
33	MSE	0.01807032	0.01831336	0.02320806
	R	0.95326653	0.9343725	0.95882688
34	MSE	0.01860283	0.01797224	0.02432325
	R	0.95167864	0.95547619	0.95712804
35	MSE	0.01778385	0.01736089	0.023583
	R	0.9443095	0.97527672	0.95981292
36	MSE	0.01820628	0.0179972	0.02431416
	R	0.97354521	0.95511923	0.926928
37	MSE	0.01780832	0.01794975	0.0225309
	R	0.94417344	0.93618605	0.95302354
38	MSE	0.01697586	0.01732352	0.02367867
	R	0.94989024	0.95569541	0.9196741
39	MSE	0.01749118	0.01740528	0.02372344
	R	0.97456392	0.93699925	0.94885266
40	MSE	0.01800225	0.01712784	0.02407808
	R	0.94594944	0.93957375	0.9185132
41	MSE	0.0177303	0.0171498	0.0221442
	R	0.94803072	0.96671806	0.95240222
42	MSE	0.01746468	0.0171411	0.02386098
	R	0.9374049	0.9393809	0.93778921
43	MSE	0.01715946	0.01714538	0.02423715
	R	0.94857792	0.9399186	0.91919625
44	MSE	0.0166216	0.01734928	0.02382287
	R	0.9771666	0.96932094	0.93795799
45	MSE	0.01726383	0.01688013	0.02241589
	R	0.95938238	0.96930526	0.95232382

S34. The experimental results for the Smart Home 6, when developing the FITNET ANNs forecasting solution using the total electricity consumption of all the individual appliances.

n	The Training Algorithm	The Levenberg-Marquardt Training Algorithm	The Bayesian Regularization Training Algorithm	The Scaled Conjugate Gradient Training Algorithm
1	MSE	0.02720947	0.02649846	0.02806524
	R	0.97944684	0.98983618	0.98904102
2	MSE	0.02604062	0.02552606	0.02712934
	R	0.99091382	0.98996699	0.99877544
3	MSE	0.02534895	0.02512026	0.02759823
	R	0.99193712	0.99002023	0.97951722
4	MSE	0.02435398	0.02343175	0.02480674
	R	0.990084712	0.99142856	0.99104255
5	MSE	0.02348758	0.02301024	0.02527529
	R	0.99211145	0.963569	0.961085
6	MSE	0.0231427	0.0224016	0.02550254
	R	0.991276595	0.98550054	0.97082614
7	MSE	0.02252437	0.02215776	0.0231024
	R	0.990526296	0.99689477	0.99234938
8	MSE	0.0214529	0.02150781	0.02455652
	R	0.99767757	0.98106249	0.97147355
9	MSE	0.02154952	0.02198394	0.02299276
	R	0.990971936	0.99198556	0.99034263
10	MSE	0.02132578	0.02015369	0.0227031
	R	0.990099417	0.98312289	0.99117905
11	MSE	0.02126208	0.01999085	0.0243542
	R	0.991084152	0.98281888	0.98057598
12	MSE	0.02060256	0.02007445	0.02214165
	R	0.99176844	0.9925467	0.99146833
13	MSE	0.02034846	0.02026836	0.02265275
	R	0.99224354	0.99029172	0.9902405
14	MSE	0.01990086	0.01996064	0.02209175
	R	0.99346164	0.99031129	0.97803047
15	MSE	0.01857535	0.01877856	0.02178336
	R	0.990386272	0.99232649	0.98796486
16	MSE	0.01937133	0.01869315	0.02220815
	R	0.992301395	0.99132117	0.99142496
17	MSE	0.0183447	0.0189385	0.02187164
	R	0.990841223	0.99450612	0.99969328

18	MSE	0.01808135	0.01878308	0.02271449
	R	0.99514056	0.99039183	0.99136018
19	MSE	0.01816495	0.01822368	0.02169132
	R	0.98477424	0.99540576	0.99102976
20	MSE	0.01805088	0.01840734	0.0232769
	R	0.990573835	0.99254552	0.97256132
21	MSE	0.0177061	0.01824074	0.02243808
	R	0.990764797	0.9983505	0.99144208
22	MSE	0.0180956	0.01808136	0.0224832
	R	0.991448152	0.99006547	0.9913355
23	MSE	0.01844271	0.01768896	0.02145888
	R	0.99812916	0.9929503	0.98943468
24	MSE	0.01828778	0.01815549	0.0213591
	R	0.98706492	0.9906172	0.99103881
25	MSE	0.01782957	0.0182655	0.02205392
	R	0.99082818	0.99185989	0.99080772
26	MSE	0.0174876	0.01746066	0.02217006
	R	0.99034742	0.99309541	0.99182039
27	MSE	0.0181002	0.0169423	0.02189088
	R	0.9971724	0.99013228	0.98121702
28	MSE	0.01773486	0.01706304	0.02189952
	R	0.99111502	0.99314686	0.99165428
29	MSE	0.01759527	0.01678935	0.02228094
	R	0.992116976	0.99227183	0.997864
30	MSE	0.01728034	0.01732542	0.02275462
	R	0.99333399	0.99222451	0.98611356
31	MSE	0.01754102	0.01656705	0.02358378
	R	0.991083376	0.984164	0.964518
32	MSE	0.01712502	0.0167904	0.02203432
	R	0.99044552	0.99229346	0.98873802
33	MSE	0.0168302	0.01690464	0.02229794
	R	0.991223147	0.99327275	0.97819712
34	MSE	0.01788039	0.01641695	0.0222384
	R	0.99092312	0.99244281	0.98613192
35	MSE	0.01609015	0.01650144	0.0220108
	R	0.993638885	0.99048306	0.99179834
36	MSE	0.01696896	0.0169589	0.02244384
	R	0.99321279	0.99240454	0.99138275
37	MSE	0.01692672	0.0167531	0.02124342

	R	0.990318428	0.99531359	0.99016472
38	MSE	0.01597728	0.01646592	0.02229933
	R	0.991915307	0.99345157	0.99068011
39	MSE	0.0164521	0.01689336	0.02212667
	R	0.991394024	0.99159045	0.98758134
40	MSE	0.0164592	0.01628824	0.02222592
	R	0.99346322	0.99384763	0.99055302
41	MSE	0.01637942	0.0163008	0.0212758
	R	0.99369086	0.99061759	0.99127578
42	MSE	0.01627776	0.01630085	0.0220077
	R	0.99660942	0.99283749	0.98612886
43	MSE	0.01631831	0.01631308	0.02192885
	R	0.990786404	0.99091758	0.97725075
44	MSE	0.01596329	0.01651518	0.02220384
	R	0.99337704	0.99385582	0.97663667
45	MSE	0.01592295	0.01654587	0.02111011
	R	0.99586162	0.99897787	0.99009118

S35. The experimental results for the Smart Home 7, when developing the FITNET ANNs forecasting solution using the total electricity consumption of all the individual appliances.

n	The Training Algorithm	The Levenberg-Marquardt Training Algorithm	The Bayesian Regularization Training Algorithm	The Scaled Conjugate Gradient Training Algorithm
1	MSE	0.02861202	0.02813754	0.0300699
	R	0.94103716	0.9129557	0.9122223
2	MSE	0.02765138	0.02682841	0.02879032
	R	0.92264064	0.92268768	0.92194656
3	MSE	0.02637315	0.0266427	0.02815577
	R	0.93263366	0.93288101	0.91229545
4	MSE	0.02584504	0.0251583	0.02607239
	R	0.93348241	0.93367544	0.9334407
5	MSE	0.02518256	0.02420869	0.02735985
	R	0.91505425	0.95393331	0.95147415
6	MSE	0.0240873	0.02403505	0.02732415
	R	0.91631205	0.93719169	0.94198972
7	MSE	0.02345321	0.02354262	0.0245463
	R	0.92793504	0.95818041	0.92490816
8	MSE	0.02325946	0.02283819	0.02556916

	R	0.92987424	0.96163551	0.95223645
9	MSE	0.02310464	0.0233163	0.02440048
	R	0.95146632	0.94215227	0.91659135
10	MSE	0.02241383	0.02140031	0.02413698
	R	0.95240222	0.94418733	0.9539739
11	MSE	0.0227808	0.02188472	0.02640508
	R	0.92336485	0.9244336	0.92289504
12	MSE	0.02189022	0.02197624	0.02447235
	R	0.95287556	0.9536233	0.93737405
13	MSE	0.02096508	0.02150928	0.0247988
	R	0.96401052	0.93475776	0.92529696
14	MSE	0.02132235	0.0213864	0.023686
	R	0.96424218	0.93494016	0.93929659
15	MSE	0.02013959	0.01995222	0.02382555
	R	0.93563904	0.93555648	0.93953521
16	MSE	0.02034968	0.02026731	0.02454585
	R	0.96455601	0.95475716	0.94663296
17	MSE	0.0194565	0.02029125	0.02321072
	R	0.93987936	0.93600576	0.93175296
18	MSE	0.01979432	0.0203322	0.02411951
	R	0.95611544	0.9259441	0.91706825
19	MSE	0.01950342	0.01936266	0.02279802
	R	0.95552352	0.96612912	0.9422968
20	MSE	0.01955512	0.01953432	0.0257271
	R	0.9569161	0.93755904	0.92441472
21	MSE	0.01938352	0.01935752	0.02407419
	R	0.93916704	0.94941175	0.95645385
22	MSE	0.01980992	0.01881192	0.0243568
	R	0.95595374	0.9319823	0.936147
23	MSE	0.01956045	0.01861026	0.02302359
	R	0.94920126	0.97067421	0.93123264
24	MSE	0.01959405	0.01927851	0.02288475
	R	0.96751908	0.96709734	0.92295065
25	MSE	0.01874862	0.019188	0.02272904
	R	0.96139764	0.94024512	0.94022585
26	MSE	0.01877616	0.01835151	0.02261794
	R	0.9315149	0.93276795	0.9212321
27	MSE	0.0190332	0.01854736	0.02277536
	R	0.9678438	0.93260455	0.96178698
28	MSE	0.01827228	0.0186627	0.02304012

	R	0.95186294	0.95288047	0.95845464
29	MSE	0.01866165	0.01837992	0.02318118
	R	0.9327993	0.94404768	0.92036
30	MSE	0.01798566	0.01785579	0.02437995
	R	0.95399403	0.94361088	0.95711022
31	MSE	0.01825698	0.01761339	0.0250131
	R	0.96176416	0.97432236	0.95487282
32	MSE	0.01764396	0.0180147	0.02315852
	R	0.9552172	0.93441145	0.94996398
33	MSE	0.01824748	0.01813727	0.02298053
	R	0.96309402	0.963879	0.9200864
34	MSE	0.01878344	0.01814505	0.0240916
	R	0.9320564	0.94562592	0.94746008
35	MSE	0.01727574	0.01804845	0.0226846
	R	0.95742589	0.94572288	0.95981292
36	MSE	0.01820628	0.01747805	0.02431416
	R	0.94404384	0.95511923	0.946239
37	MSE	0.0185136	0.01760785	0.02188716
	R	0.95400858	0.93618605	0.95302354
38	MSE	0.01730872	0.01732352	0.02321889
	R	0.96967962	0.93599035	0.95839722
39	MSE	0.01749118	0.01757592	0.02303911
	R	0.9351876	0.93699925	0.91980615
40	MSE	0.0178308	0.01712784	0.02361504
	R	0.9360958	0.949464	0.93785032
41	MSE	0.01722372	0.0174894	0.0221442
	R	0.95790604	0.94698912	0.93296544
42	MSE	0.01729512	0.0174772	0.02362932
	R	0.96700716	0.94926912	0.95712507
43	MSE	0.01749592	0.01714538	0.02377549
	R	0.95845894	0.94981248	0.928872
44	MSE	0.01759905	0.01684882	0.02428545
	R	0.9641897	0.94953888	0.92828832
45	MSE	0.0166214	0.01721439	0.02263352
	R	0.9701635	0.97919613	0.94260623

S36. The experimental results for the Smart Home 8, when developing the FITNET ANNs forecasting solution using the total electricity consumption of all the individual appliances.

n	The Training Algorithm	The Levenberg-Marquardt Training Algorithm	The Bayesian Regularization Training Algorithm	The Scaled Conjugate Gradient Training Algorithm
1	MSE	0.02692896	0.0259521	0.02749248
	R	0.96984442	0.98983618	0.99082457
2	MSE	0.02657754	0.02526559	0.02685251
	R	0.97069484	0.990918965	0.98917183
3	MSE	0.02483685	0.02435904	0.02704069
	R	0.99095519	0.98096766	0.99872344
4	MSE	0.02385696	0.0241717	0.02480674
	R	0.99122359	0.99106796	0.99008024
5	MSE	0.0230033	0.02372931	0.02579643
	R	0.99211145	0.963569	0.961085
6	MSE	0.0231427	0.0228683	0.02472185
	R	0.99031206	0.990482408	0.99005042
7	MSE	0.02252437	0.02261938	0.02382435
	R	0.99559697	0.98721618	0.99019838
8	MSE	0.02167872	0.02172954	0.0240502
	R	0.99073638	0.991991645	0.97147355
9	MSE	0.02199384	0.02176188	0.02275814
	R	0.98059284	0.991985555	0.97448133
10	MSE	0.02110817	0.01973815	0.02342004
	R	0.99127578	0.99285678	0.9732461
11	MSE	0.02104512	0.02083257	0.02537964
	R	0.99205612	0.990228064	0.98057598
12	MSE	0.02060256	0.02049707	0.02214165
	R	0.99176844	0.990227755	0.99535595
13	MSE	0.0195263	0.0196479	0.0233681
	R	0.98348548	0.990291718	0.97348951
14	MSE	0.01949472	0.02016432	0.02209175
	R	0.99032015	0.98363496	0.97803047
15	MSE	0.01935747	0.01936539	0.02178336
	R	0.98437024	0.98428338	0.98796486
16	MSE	0.01917566	0.01948023	0.02290946
	R	0.9913271	0.990346926	0.98527104
17	MSE	0.0176035	0.01835875	0.02142528
	R	0.99862182	0.98475606	0.9909399

18	MSE	0.01846201	0.01897672	0.02224615
	R	0.99514056	0.990391834	0.99429505
19	MSE	0.01835616	0.01879317	0.02124864
	R	0.9914025	0.991492352	0.9908688
20	MSE	0.01842694	0.01821951	0.0232769
	R	0.99155028	0.99254552	0.99181996
21	MSE	0.01826524	0.01805461	0.02267181
	R	0.98808199	0.992771375	0.9854373
22	MSE	0.01885752	0.01753344	0.0231858
	R	0.99242362	0.990065468	0.9903704
23	MSE	0.01844271	0.0175047	0.02168241
	R	0.98834358	0.990008858	0.99088354
24	MSE	0.01772795	0.01852983	0.02070525
	R	0.99163837	0.990617198	0.99006728
25	MSE	0.01782957	0.0178965	0.02227896
	R	0.9900689	0.990880466	0.99177703
26	MSE	0.01785576	0.01746066	0.02194612
	R	0.99099583	0.99167961	0.99880954
27	MSE	0.0182868	0.01712064	0.0210064
	R	0.9926501	0.99077345	0.99006471
28	MSE	0.01755572	0.01741852	0.0216714
	R	0.99205541	0.99146855	0.97781736
29	MSE	0.01723981	0.01696608	0.02183082
	R	0.99171294	0.990305066	0.978488
30	MSE	0.01728034	0.01750221	0.02275462
	R	0.9913004	0.990258656	0.99578134
31	MSE	0.01772001	0.01674144	0.02286912
	R	0.99010198	0.984164	0.964518
32	MSE	0.01695204	0.0169653	0.0213598
	R	0.99044552	0.99342691	0.99178186
33	MSE	0.01736168	0.01725682	0.02184288
	R	0.99257649	0.9922892	0.97819712
34	MSE	0.01715795	0.01641695	0.02293335
	R	0.9901676	0.992442808	0.99151358
35	MSE	0.01625952	0.01684522	0.0220108
	R	0.99690737	0.990483056	0.99859324
36	MSE	0.01749924	0.0166128	0.02244384
	R	0.99128804	0.990435218	0.984861
37	MSE	0.0167504	0.01692405	0.02102884

	R	0.99334914	0.991502277	0.99210967
38	MSE	0.01581085	0.0162944	0.02183955
	R	0.99290478	0.99451565	0.99068011
39	MSE	0.01662528	0.0162108	0.02235478
	R	0.99040962	0.99060413	0.99726351
40	MSE	0.0164592	0.0159524	0.0219944
	R	0.99050713	0.9928586	0.99055302
41	MSE	0.01637942	0.0163008	0.0212758
	R	0.99740732	0.991604041	0.99127578
42	MSE	0.01627776	0.01630085	0.02223936
	R	0.99660942	0.991848666	0.99151327
43	MSE	0.01598185	0.01631308	0.02239051
	R	0.9907864	0.990917576	0.9906278
44	MSE	0.01579872	0.01618154	0.02197255
	R	0.99039501	0.99899403	0.99056457
45	MSE	0.01625817	0.01654587	0.02132774
	R	0.9985067	0.991875961	0.99119418

S37. The comparison of the best experimental results recorded for Smart Homes 1-8 when developing the FITNET ANNs forecasting solution using the total electricity consumption of all the individual appliances. The forecasting results of the Smart Home 3, that are presented in detail in the paper, are highlighted in red.

The Best Forecasting Results								
**The Smart Home number**	**1**	**2**	**3**	**4**	**5**	**6**	**7**	**8**
**The training algorithm**	BR	LM	LM	BR	LM	LM	LM	BR
**The number of neurons in the hidden layer n**	45	40	44	45	44	45	45	44
**MSE**	0.01547735	0.0168094	0.016457	0.01587735	0.0166216	0.01592295	0.0166214	0.01618154
**R**	0.99385414	0.9755104	0.994010	0.99586505	0.9771666	0.99586162	0.9701635	0.99899403
